# The Photochemical Activity of a Halogen-Bonded Complex Enables the Microfluidic Light-Driven Alkylation of Phenols

**DOI:** 10.1021/acs.orglett.2c00604

**Published:** 2022-04-19

**Authors:** Sara Cuadros, Cristian Rosso, Giorgia Barison, Paolo Costa, Marina Kurbasic, Marcella Bonchio, Maurizio Prato, Giacomo Filippini, Luca Dell’Amico

**Affiliations:** † Department of Chemical Sciences, University of Padova, Via Marzolo 1, 35131 Padova, Italy; ‡ Department of Chemical and Pharmaceutical Sciences, CENMAT, Center of Excellence for Nanostructured Materials, INSTM UdR, Trieste, University of Trieste, Via Licio Giorgieri 1, 34127 Trieste, Italy; § INSTM UdR, Instituto per la Tecnologia delle Membrane, ITM-CNR, UoS di Padova, via Marzolo 1, 35131 Padova, Italy; ∥ Center for Cooperative Research in Biomaterials (CIC biomaGUNE), Basque Research and Technology Alliance (BRTA), Paseo de Miramón 194, 20014 Donostia, San Sebastián, Spain; ⊥ Basque Fdn Sci, Ikerbasque, 48013 Bilbao, Spain

## Abstract

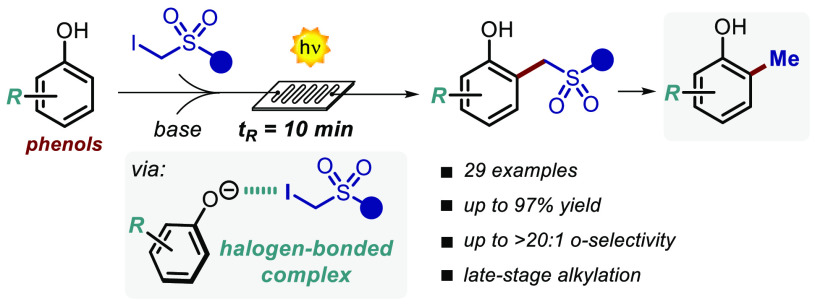

A mild light-driven protocol for the direct alkylation of phenols is reported. The process is driven by the photochemical activity of a halogen-bonded complex formed upon complexation of the *in situ* generated electron-rich phenolate anion with the α-iodosulfone. The reaction proceeds rapidly (10 min) under microfluidic conditions, delivering a wide variety of ortho-alkylated products (27 examples, up to 97% yield, >20:1 regioselectivity, on a gram scale), including densely functionalized bioactive phenol derivatives

Phenols are highly relevant functional groups in synthetic and industrial chemistry. As a matter of fact, these aromatic systems are present in a wide number of biologically relevant compounds, such as tyramine, dopamine, paracetamol, among others (Figure 1a).^1^ Furthermore, phenol derivatives are key components of functional materials, including biopolymers, melanin,^2^ and lignin.^3^ Given their ubiquitous presence in different research areas, the development of methods allowing the chemical diversification of phenolic substrates, through a direct site-selective C–H functionalization process, is of great importance.^5^ Progress in this field has been spurred through the identification of transition-metal or organic catalysts capable to induce ortho (*o*)- or para (*p*)- selectivity in either electrophilic aromatic substitution (E_Ar_S) or cross-coupling reactions (Figure 1b).^5,6^ However, these methods are limited to tailored substituted phenol derivatives, while requiring high temperatures or potentially toxic catalysts. On the other hand, the classical homolytic aromatic substitution (HAS) provides a sustainable alternative method that enables the direct C–H functionalization of aromatic substrates through a radical pathway.

**Figure 1:**
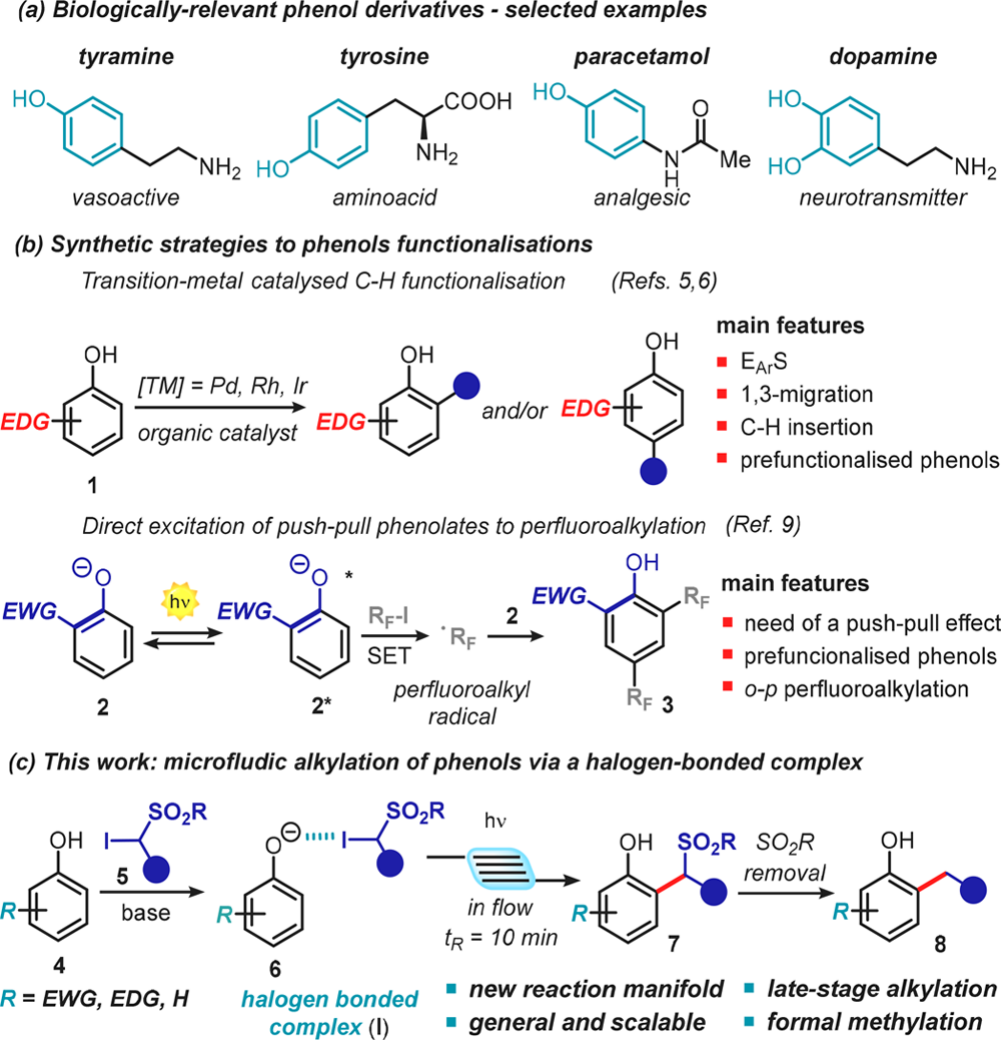
(a) Biologically relevant phenols. (b) Reported approaches for the functionalization of phenols. (c) This work.

Nevertheless, the generation of open-shell intermediates engaging in HAS conventionally requires harsh reaction conditions, thus limiting a widespread use. Interestingly, the deprotonation of phenols **1** gives access to phenolate anions **2**, which are photochemically active electron-rich intermediates that can be used to generate reactive open-shell species (Figure 1b).^8^

In fact, an alternative approach to the use of transition-metal catalysts is the direct excitation of the phenolate, which has been used toward the perfluoroalkylation of electron-poor derivatives.^9^ Nevertheless, also this approach requires prefunctionalized substrates,^10^ while leading to polyperfluoroalkylated products. We thus questioned if general phenolates **6** (R = H, EDG, or EWG) can be involved in the formation of photoactive halogen-bonded complexes with suitable partners.^8,11^ One important feature of this ground-state molecular aggregate is that, typically, its absorption profile exhibits a bathochromic shift.^8,12^ The excitation of the halogen-bonded complex can then occur at red-shifted wavelengths, triggering a single-electron-transfer (SET) event within the aggregate, while leading to reactive radical species.

Herein, we report a general microfluidic light-driven method for the C–H alkylation of phenols **4** using α-iodosulfones **5** (Figure 1c).^13,14^ The chemistry exploits the ability of the phenolate anion **6** to form a halogen-bonded complex with **5**. Upon light irradiation of the resulting complex **I**, an electrophilic (phenylsulfonyl)alkyl radical is produced, which subsequently engages in a HAS process leading to the alkylated phenols **7**. It is noteworthy that this unprecedented microfluidic halogen-complex-based strategy does not require the use of external photoredox or metal catalysts. Importantly, the implementation of this photochemical process in flow allows very short reaction times (10 min) with a high productivity rate (up to 0.6 mmol·h^–1^) and good to excellent *o*-selectivity (up to >20:1). Finally, the sulfonyl group removal under simple reductive treatment led access to important methylated bioactive phenol derivatives, extremely challenging to obtain under conventional synthetic methods.

Our exploratory studies began by testing the feasibility of a halogen-bonded association between the phenolate **6a** and the α-iodosulfone **5a** (Figure 2).

**Figure 2:**
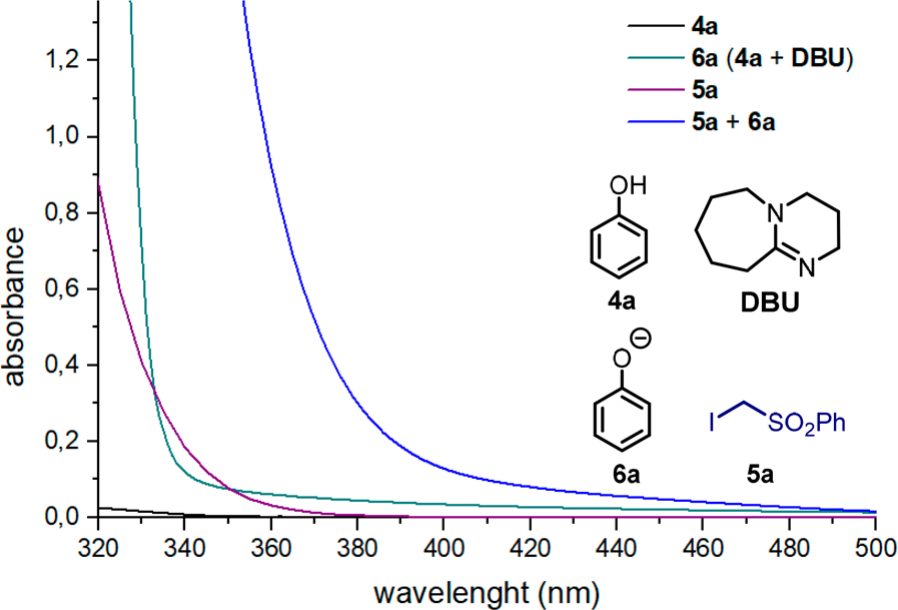
Optical absorption spectra acquired in acetonitrile in 1 mm path quartz cuvettes: [**4a**] = 1.5 M; [**5a**] = 0.5 M; [DBU] = 1.5 M. DBU: 1,8-diazabiciclo[5.4.0]undec-7-ene.

The optical absorption spectra showed a clear, red-shifted charge-transfer (CT) band, confirming the formation of a halogen-bonded complex and the viability of our approach (blue line in Figure 2). Interestingly, the isolated chemical species (**4a**, **5a**, and **6a**) showed a negligible absorption at λ > 370 nm.

Capitalizing on these observations, we next evaluated the feasibility of the photochemical alkylation process between **4a** and **5a**, using a microfluidic photoreactor (MFP) equipped with a modular light source (370–456 nm, Table 1). The optimization process was directly performed under microfluidic conditions, since this provides a more efficient irradiation of the reaction mixture, allowing an easy scaling-up, and shorter reaction times that reduces the generation of overalkylation products.^15^ Specifically, the use of DBU as base, a Kessil lamp at 405 nm, and setting a residence time (*t*_R_) of 10 min led the formation of the alkylated product **7a** as a mixture of the *ortho*- (*o*-) and *ortho*,*ortho*′- (*o*,*o*′-) adducts in a 3.1:1 ratio with a total yield of 10% (entry 1, Table 1). Changing the wavelength to 370 nm, where the absorption of the halogen-bonded complex is higher (blue line in Figure 2), resulted in 26% yield and a 3.3:1 ratio (entry 2, Table 1). Interestingly, increasing the initial concentration to 0.5 M, thus favoring the halogen-bonded complex formation, resulted in further improvements with a yield of 33% (entry 3, Table 1). Finally, using 1,1,3,3-tetramethylguanidine (TMG) as base,^16^ we obtained product **7a** in a promising 10:3:1 ratio for the *o*:*o*,*o*′:*p* adducts with an overall yield of 63% (entry 4, Table 1).^17^ It is worth noting that the observed regioselectivity value is highly promising, also in light of the radical nature of this transformation.^7,9^

**Table 1:**
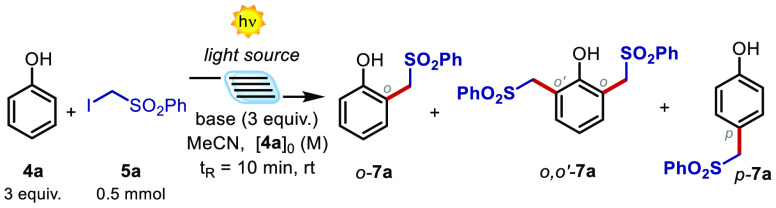
Optimization of the Reaction Conditions and Control Experiments (Selected Results)^a^

entry	light source (nm)	[**4a**]_0_ (M)	base	yield (%)^b^	*o*:*o*,*o*′:*p* ratio^b^
1	405	0.25	DBU	10	3.1:1:n.d.
2	370	0.25	DBU	26	3.3:1:n.d.
3	370	0.5	DBU	33	3.3:1:n.d.
4	370	0.5	TMG	63	10:3:1
5^c^	370	0.5	TMG	47	10:3:1
6^d^	370	0.5	TMG	43	10:3:1
7	light off	0.5	TMG	0	

a Reactions were performed at ambient temperature in a microfluidic photoreactor (internal diameter: 0.8 mm).

b The yield and the regioisomeric ratio were determined by ^1^H NMR analysis, using trichloroethylene as internal standard.

c 2 equiv of TMG were used.

d Reaction under batch-setup in 18h.

Additionally, all the products (*o*-**7a**, *o*,*o*′-**7a**, and *p*-**7a**) were easily separated by flash chromatography, thus increasing the synthetic utility of this photochemical process. Control experiments confirmed that an inferior amount of base and a normal batch setup highly reduce the productivity of the system (entries 5 and 6).^18^

In order to gain insights on the nature of the complex between the phenolate **6a** and the α-iodosulfone **5a**, we performed DFT calculations at the M06-2x/Def2TZVP level of theory including polarizable continuum solvation model (MeCN). As shown in Figure 3a, natural charge distribution and the related plot of electrostatic potential indicate that the electron density in **6a** is mostly localized at the oxygen atom while a positive area is present at the iodine atom (σ-hole) in **5a**. Upon searching for minima structures between **6a** and **5a** (see the SI), we found that the two most stable complexes are characterized by (i) a hydrogen bond between a methylene proton of the α-iodosulfone **5a** and the oxygen of **6a** (O···H = 1.93 Å) in addition to a π–π interaction between the aromatic rings of **5a** and **6a** (C···C = 3.46 Å, Figure 3b) and (ii) a halogen-bonding interaction involving the oxygen and the iodine atom of **5a** and **6a** (O···I = 2.60 Å, Figure 3b). Although both complexes display very similar binding energy (Δ*E*), −7.8 and −7.2 kcal mol^–1^, for (i) and (ii), respectively, the computed UV–vis absorption spectrum of the halogen-bonded complex better resembles the experimental one, indicating the possible activity of this type of complex under the reaction conditions (Figure 3b).^19,20^ This hypothesis is also corroborated by the experiments performed using α-iodosulfones lacking the aromatic ring (*vide infra*Scheme 1).

**Figure 3:**
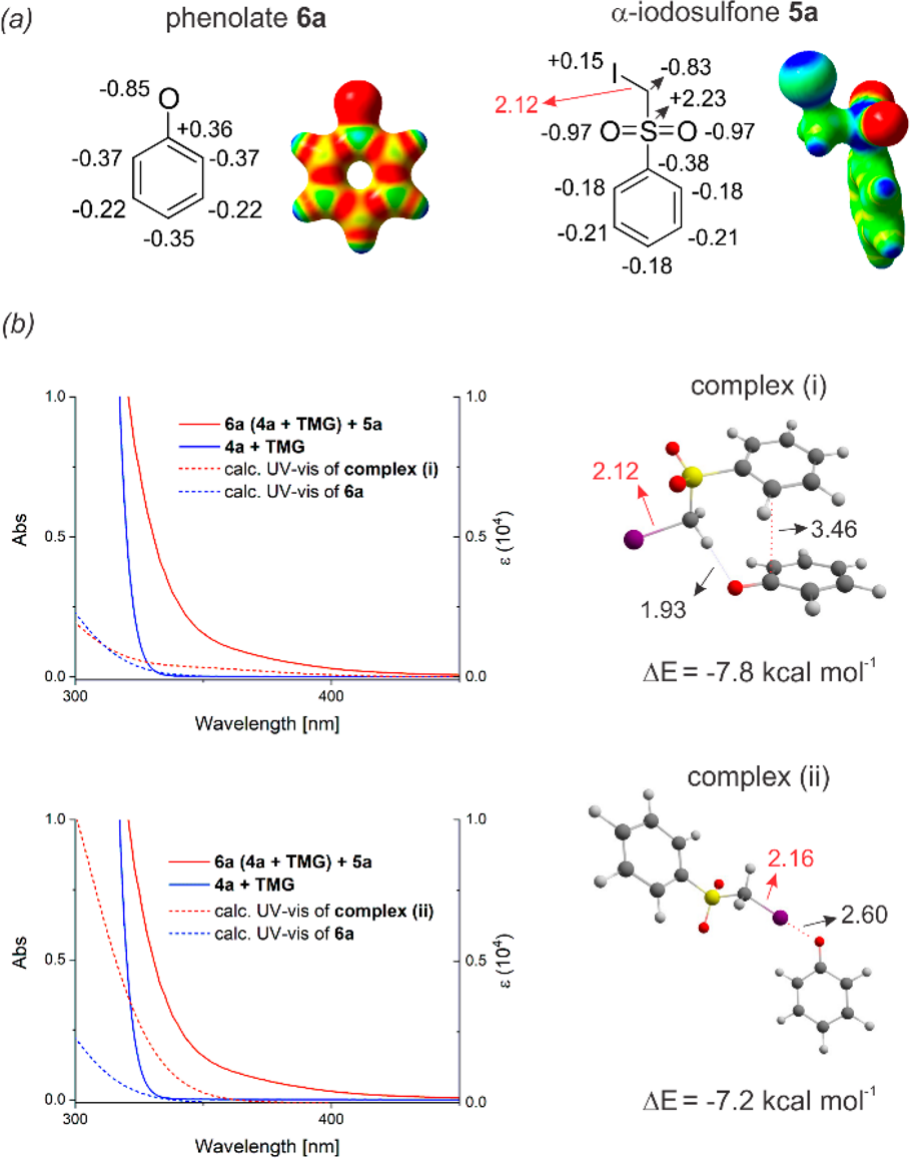
(a) Natural charge analysis and electrostatic potential (blue, positive potential; red, negative). (b) Absorption spectra for the reaction mixture (0.5 M) in the absence of **5a** (blue solid line) and in the presence of **5a** (red solid line). Dashed lines represent the theoretical absorption spectra computed at the M06-2X/Def2TZVP, SCRF = (IEFPCM, MeCN) level of theory of complexes (i) and (ii). Δ*E* represents the binding energies corrected with zero-point vibrational energy.

**Scheme 1:**
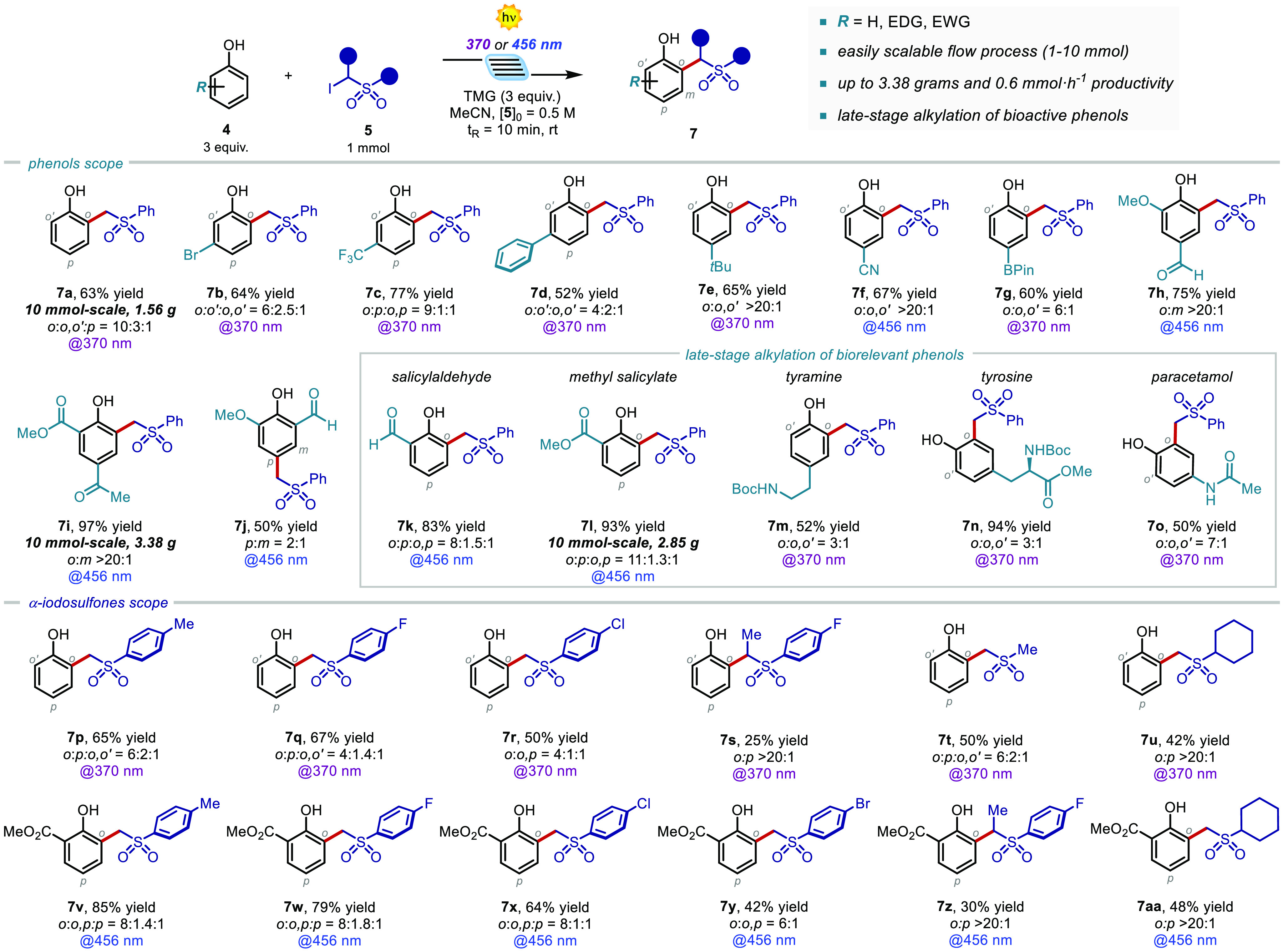
Scope of Phenols 4 and α-Iodosulfones 5 That Can Participate in the Microfluidic Photoalkylation Process Reactions performed on a 1 mmol scale (see the SI for details). The reported yields refer to the total yield of the diverse regioisomers.

These substrates, lacking the phenyl ring, lose the ability to form relevant π–π interactions, while maintaining a strong ability in generating halogen-bonded complexes.^21^ In this regard, the calculations also indicate that the formation of a halogen-bonded adduct between **5a** and **6a** results in a sensible weakening of the C–I bond (C–I = 2.16 Å) of the α-iodosulfone. On the other hand, within complex (i) the length of this chemical bond remains virtually unvaried. This suggests that the halogen-bonding interaction is a key factor to favor the mesolytic cleavage of **5a** and consequently the radical initiation step.^22^ We next performed quantum yield (QY) measurements to get information on the mechanism of this radical transformation.

For the reaction of phenol **4a** and **5a**, we determined a QY value of 2 mol of **7a** per mole of photons, indicating the activity of a radical chain process.^23^ On these grounds, we propose the mechanism depicted in Figure 4. After the formation of the phenolate **6a**, the halogen-bonding complex **Ia** is formed with the α-iodosulfone **5a**. The direct excitation promotes a SET event within **Ia**, leading to the radicals **IIa** and **IIIa** along with the release of I^–^. The open-shell intermediate **IIa** is rapidly trapped in a reversible fashion by the ground state phenolate **6a**, forming the radical anion **Va**. Inspection of the natural charge distribution of the phenolate **6a** (Figure 3a) suggests that the sulfonyl radical **IIa**, behaving as a highly electrophilic species,^24^ should almost equally add to the electron-richer *ortho* and *para* positions of **6a**. Thus, the high *o*-selectivity observed can be rather ascribed to the relative stability of the transient radical anion **Va** versus the *para*-adduct,^25^ which entails that the reaction is under thermodynamic control.^26^ In addition to this, the *o*-selectivity can also be rationalized by considering the statistically favored *ortho* attack (2:1 versus the *para* position). Considering the relatively low redox potential of the α-iodosulfone **5a** [*E*_red_ (**5a**/**5a**^**•–**^) = −1.4 V vs SCE in MeCN)^14^ the propagation of the chain could occur by another SET event from **Va** to **5a**, generating another molecule of the reactive radical intermediate **IIa** and **IVa**. By keto–enolic tautomerism of **IVa** the final alkylated phenol *o*-**7a** is formed. The chain process is terminated by a radical–radical coupling between the intermediates **IIIa** and **IIa**, ultimately leading to the formation of a molecule of product *o*-**7a**. Also, the product *o*-**7a** can potentially take part in the alkylation process, leading to the formation of bis-alkylated products.

**Figure 4:**
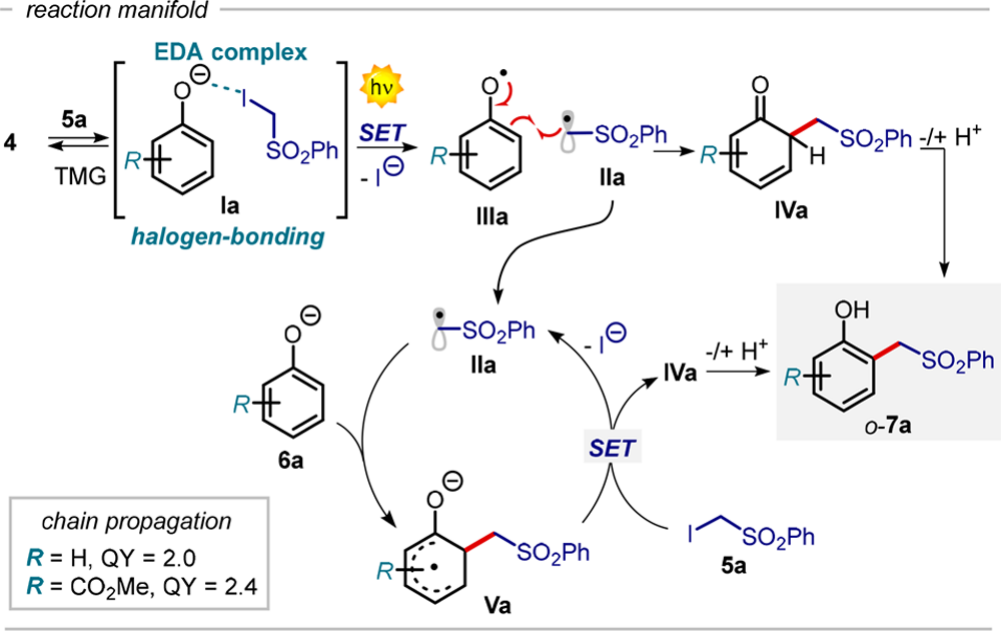
Proposed mechanism of the photoalkylation of phenols **4** with the α-iodosulfone **5a**.

With a clear mechanistic picture of the developed light-driven process, we evaluated the generality of the microfluidic photochemical alkylation process for a variety of structurally diverse phenols (Scheme 1). Phenols bearing different types of *m-*substituents were competent substrates, delivering the alkylated products **7b**–**d** in yields up to 77% and selectivity up to 9:1:1 for *o*:*p*:*o*,*p*. In addition, the *p*-substitution was well tolerated, accessing **7e**–**g**, bearing a *t-*butyl, cyano, and boronic acid pinacol ester moiety, in yields up to 67%, and up to >20:1 regioselectivity. When using phenols bearing EWGs, the corresponding complexes **I** showed a strong, red-shifted absorption (see the SI), thus allowing the use of a visible-light source (465 nm).^8^ Disubstituted precursors, derived from natural lignin biopolymer, effectively participated in the developed light-driven alkylation, furnishing the alkylated products **7h**–**j** in up to 97% yield and >20:1 regioselectivity. Remarkably, the *o*-substituted products **7k** and **7l**, deriving from biologically relevant salicylates, were obtained in excellent yield (up to 93%) and regioselectivity. Furthermore, other bioactive electron-rich phenols, such as tyramine, tyrosine, and paracetamol were also successfully alkylated (up to 94% yield and 7:1 *o*:*o*,*o*′), thus demonstrating the synthetic potential of this method for the late-stage alkylation of bioactive phenols.

Importantly, the reactions giving products **7a**, **7i**, and **7l** were easily scaled up to 10 mmol, with a high productivity (up to 0.6 mmol·h^–1^) and delivering up to 3.38 g of the alkylated product. We next assessed the scope of the α-iodosulfone. As shown in Scheme 1, various derivatives efficiently reacted with up to 85% yield and >20:1 regioselectivity (**7p**–**r** and **7v**–**y**). Noteworthy, also secondary carbons were readily installed onto the phenol scaffolds (**7s** and **7z**, up to 30% yield and >20:1 regioselectivity). In addition, this photochemical method allows the use of α-iodoalkyl sulfones as radical precursors. Remarkably, products **7t**–**u** and **7aa** were isolated in up to 50% yield and excellent regioselectivity (up to >20:1). To further demonstrate the synthetic potential of the developed photochemical alkylation process, we performed a reductive desulfonylation of the alkylated products *o*-**7a** and *o*,*o*′-**7a**, which are easily separated by flash column chromatography after the microfluidic photochemical alkylation process. As shown in Figure 5a, we accessed the corresponding methylated targets **8a** and **8b** in high isolated yields.^27^ While classical methylation reactions often lead to complex mixture of regioisomers,^4^ this two-step methylation process allows the selective generation of the single methylated regioisomers (**8a**–**d**), without the need of HPLC separation. Likewise, the reductive desulfonylation of the phenol **7d**, and the paracetamol derivative **7o**, afforded the methylated products **8c** and **8d** in 75% and 81% yields, respectively (Figure 5b). It is worth noting that there are no other existing methodologies enabling the simple methylation of **4o** to access the paracetamol derivative **8d**.

**Figure 5:**
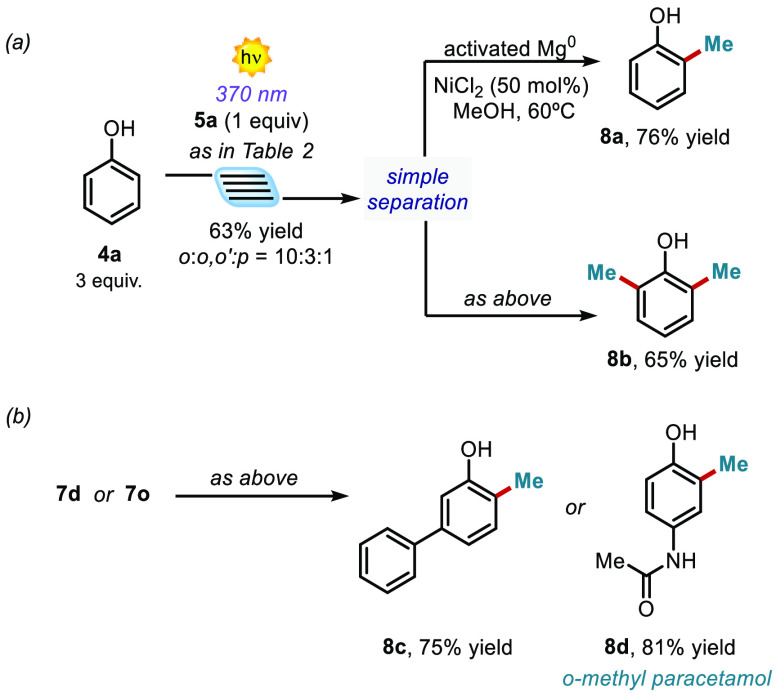
(a) Two-step methylation process of the unsubstituted phenol **4a**. (b) Two step *ortho*-methylation process of the substituted phenol **4d** and paracetamol **4n**.

In conclusion, we have developed a new photochemical strategy that enables the microfluidic C–H alkylation of phenols without the use of any external photoredox or metal catalyst. The developed method relies on the formation of a photoactive halogen-bonded complex between the phenolate anion and the α-iodosulfone. The excitation of the halogen-bonded complexes enables the formation of reactive alkyl radicals, engaging in a HAS processes with the ground state phenolates. Remarkably, this halogen-complex-based strategy enables the alkylation of a wide variety of substrates, including biologically relevant tyrosine, paracetamol, and tyramine. Furthermore, the sulfonyl moiety can be easily removed upon reductive cleavage, establishing a new straightforward strategy to access elusive methylated phenols.

## Supporting Information Available

Experimental procedures, characterization data, and optical absorption, UV, and NMR spectra, CV, coordinates (PDF)
