# Supplementary material for: The Photochemical Activity of a Halogen-Bonded Complex Enables the Microfluidic Light-Driven Alkylation of Phenols
Source: Org Lett. 2022 Apr 19;24(16):2961–6. doi: 10.1021/acs.orglett.2c00604 (PMC9062880; doi:10.1021/acs.orglett.2c00604)
Supplement: Supplementary file 1 — ol2c00604_si_001.pdf [file ol2c00604_si_001.pdf]

Supporting Information

The Photochemical Activity of a Halogen-Bonded Complex Enables the Microfluidic Light-Driven Alkylation of Phenols

Sara Cuadros,[⁋,‡] Cristian Rosso,[§,‡] Giorgia Barison,[⁋] Paolo Costa,[⁋] Marina Kurbasic,[§] Marcella Bonchio,[⁋,ǂ] Maurizio Prato,[§,‖,⊥] Giacomo Filippini[*§] and Luca Dell'Amico,[*⁋]

[⁋]*Department of Chemical Sciences, University of Padova, Via Marzolo 1, 35131, Padova, Italy.*

[§]*Department of Chemical and Pharmaceutical Sciences, CENMAT, Center of Excellence for Nanostructured Materials, INSTM UdR, Trieste, University of Trieste, Via Licio Giorgieri 1, 34127 Trieste, Italy.*

[ǂ]*Department of Chemical Sciences, INSTM UdR, University of Padova, and Instituto per la Tecnologia delle Membrane, ITM-CNR, UoS di Padova, via Marzolo 1, 35131, Padova, Italy*

[‖]*Center for Cooperative Research in Biomaterials (CIC bio-maGUNE), Basque Research and Technology Alliance (BRTA), Paseo de Miramon 194, 20014 Donostia San Sebastián, Spain.*

[⊥]*Basque Fdn Sci, Ikerbasque, 48013 Bilbao, Spain*

*Correspondence to: luca.dellamico@unipd.it; gfilippini@units.it;

Table of contents

A. GENERAL INFORMATION S3

A.1. LIGHT SOURCES EMISSION SPECTRA S4

A.2. MICROFLUIDIC PHOTOREACTOR SETUP S4

B. GENERAL PROCEDURES FOR THE SYNTHESIS OF STARTING MATERIALS S6

B.1. PREPARATION OF SUBSTITUTED PHENOLS (4m and 4n). S6

B.2. PREPARATION OF α-IODOSULFONES S7

B.2.1. Synthesis of 5a-d and 5f-h S7

B.2.2. Synthesis of the secondary α-iodo sulfone 5e S9

C. GENERAL PROCEDURE FOR THE MICROFLUIDIC PHOTOALKYLATION OF PHENOLS S10

D. OPTIMIZATION OF THE REACTION CONDITIONS S25

E. UNSUCCESFULL SUBSTRATES S27

F. PRODUCT MANIPULATIONS S27

F.1. DESULFONYLATION OF THE PRODUCTS 7a, 7d AND 7o S27

G. MECHANISTIC INSIGHTS S29

G.1. ABSORPTION SPECTRA S29

G.2. CYCLIC VOLTAMMETRY MEASUREMENTS S31

G.3. DFT STUDIES S32

G.4. QUANTUM YIELD MEASUREMENT S41

H. REFERENCES S46

I. NMR SPECTRA S48

A. GENERAL INFORMATION

NMR spectra were recorded on Bruker 400 Avance III HD equipped with a BBI-z grad probe head 5mm, Bruker 500 Avance III equipped with a BBI-ATM-z grad probe head 5mm or Bruker 200 equipped with a QNP probehead (^{1}H: 400 MHz, ^{13}C: 100.5 MHz and 376 MHz for ^{1}H decoupled ^{19}F). The chemical shifts (δ) for ^{1}H and ^{13}C are given in ppm relative to residual signals of the solvents ($CHCl_3$ @ 7.26 ppm for ^{1}H NMR, and @ 77.16 ppm for ^{13}C NMR; $CFCl_3$ @ 0.0 ppm for ^{19}F NMR spectra). Coupling constants are given in Hz. The following abbreviations are used to indicate the multiplicity: s, singlet; d, doublet; t, triplet; q, quartet; m, multiplet; br, broad signal. NMR yields were calculated by using trichloroethylene as internal standard. Structural assignments were made with additional information from gCOSY, gHSQC, and gHMBC experiments.

High-Resolution Mass Spectra (HRMS) were obtained using Waters GCT gas chromatograph coupled with a time-of-flight mass spectrometer (GC/MS-TOF) with electron ionization (EI).

Absorption spectroscopy studies have been performed on a Varian Cary 50 UV-Vis double beam spectrophotometer (more info at: www.varianinc.com). All the spectra were recorded at room temperature using a 1 mm or 10 mm path length Hellma Analytics quartz cuvettes.

All the cyclic voltammograms were recorded with a scan rate of 0.1 V/s. A typical three-electrode cell was employed, which was composed of a glassy carbon (GC) working electrode (3 mm diameter), a platinum wire as counter electrode and a saturated aqueous calomel electrode (SCE) as reference electrode. The glass electrochemical cell was kept closed with a stopper annexed to the potentiostat. Oxygen was removed by purging the solvent with high-purity Nitrogen (N_2), introduced from a line into the cell by means of a glass pipe. The potential of ferrocenium/ferrocene (Fc^+/Fc) couple was used as internal reference system to calibrate the potentiostat. All the results are subsequently converted in V vs SCE, in agreement with the value reported in literature [$E_{1/2}$(Fc^+/Fc) = +0.38 V vs SCE].[20]

Light sources: The light sources used in this work are the following and were purchased from Kessil, Amazon or eBay webpages:

- **370 nm:** Kessil lamp PR160L-370 (43W). (https://www.kessil.com/science/PR160L.php)

- **405 nm:** JUNERAIN lamp 2nn5lm2af5sx9vw4D02 (6W). (https://www.amazon.it/JUNERAIN-Lampada-fotopolimerizzante-Stampante-fotosensibile/dp/B07KJH2ZPK).

- **456 nm:** Kessil lamp PR160L-456 (50W). (https://www.kessil.com/science/PR160L.php).

The quantum yield measurement was performed with a ferrioxalate actinometer, following a procedure previously described in literature.[1]

General Procedures. The continuous flow reactions were carried out using capillary reactors made with FEP tubing (0.8 mm I.D., 1.58 mm O.D.) and fitting connections purchased from

BGB® (www.bgb-info.com). Reagents were pumped using Syrris Atlas pump (www.syrris.com). Chromatographic purification of products was accomplished using flash chromatography on silica gel (SiO_2, 0.04-0.063 mm) purchased from Machery-Nagel, with the indicated solvent system according to the standard techniques. Thin-layer chromatography (TLC) analysis was performed on pre-coated Merck TLC plates (silica gel 60 GF254, 0.25 mm). Visualization of the developed chromatography was performed by checking UV absorbance (254nm) as well as with aqueous ceric ammonium molybdate and potassium permanganate solutions. Organic solutions were concentrated under reduced pressure on a Büchi rotary evaporator.

Materials. Commercial grade reagents and solvents were purchased at the highest commercial quality from Sigma Aldrich or FluoroChem and used as received, unless otherwise stated. Phenols **4a**, **4b**, **4c**, **4d**, **4e**, **4f**, **4g**, **4h**, **4i**, **4j**, **4k, 4l** and **4o** were purchase from Sigma Aldrich or Fluorochem and used as received, unless otherwise stated. The procedures for the preparation of phenols **4m** and **4n** and the α-iodosulfones **5a-h** are detailed in Section B.1 of this Supplementary Information (SI).

Computational details. All geometry optimizations were performed using M06-2X functional,[3] including the integral equation formalism of the polarizable continuum model (IEFPCM) to model solvation effects.[4] The def2TZVP basis set was employed.[5] Frequencies were calculated in order to ensure that the structures are actual relative minima of energy. TD-DFT calculations were performed at the same level of theory on the optimised structures to compute the corresponding UV-vis absorption spectra. All the calculations were carried out using Gaussian 09 software package.[6]

A.1. LIGHT SOURCES EMISSION SPECTRA

370 nm and 456 nm Kessil PR160L lights

The following spectrum is reported in the Kessil website (www.kessil.com/science/PR160L.php). The wavelengths used in the current study were set at 370 and 456 nm.

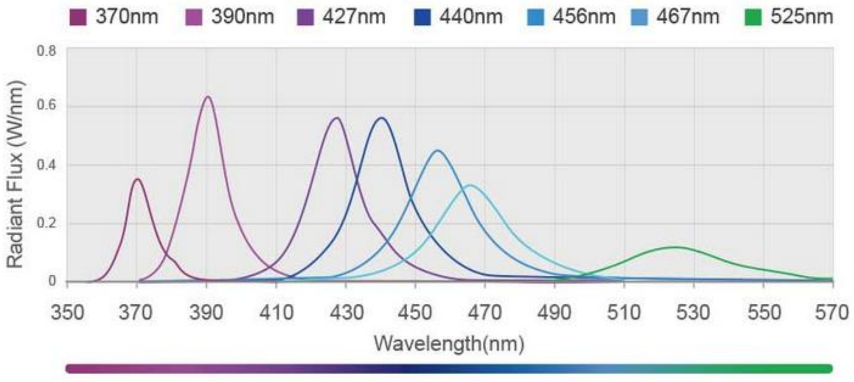

Figure S1. Emission spectra of the Kessil lights used in this work.

A.2. MICROFLUIDIC PHOTOREACTOR SETUP

Figure S2 shows a schematic representation of the microfluidic circuit employed in the present study. Reaction mixtures are introduced in continuous flow into the micro-photoreactor via a

double syringe pump (Syrris Atlas, see general information). The microfluidic reactor consists of a transparent TFE capillary (BGB®; internal diameter: 800 µm; inner volume: 210 µL; microreactor tubing length: 42 cm). The microreactor is then irradiated by the selected light source. The crude is collected in a vial, connected to the exit of the microreactor.

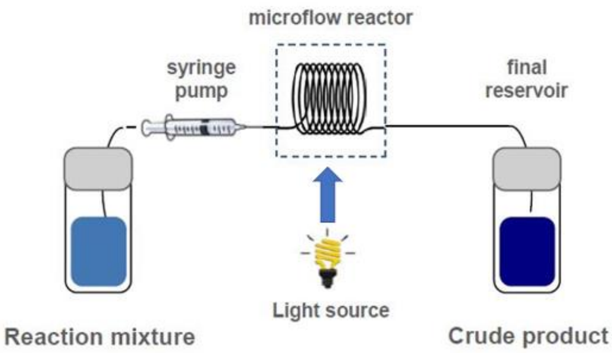

Figure S2. Schematic representation of the micro-photoreactor setup.

Figure S3 shows the assembled microfluidic reactor (left side) and the general setup of a reaction using a Kessil lamp (right side). The lamp is placed at a fixed distance of 1 cm. Aluminium foil is used to avoid undesired irradiation of the tubing. To maintain a stable reaction temperature, a fan is placed at 3 cm from the reactor.

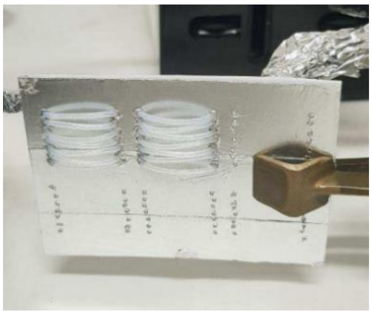

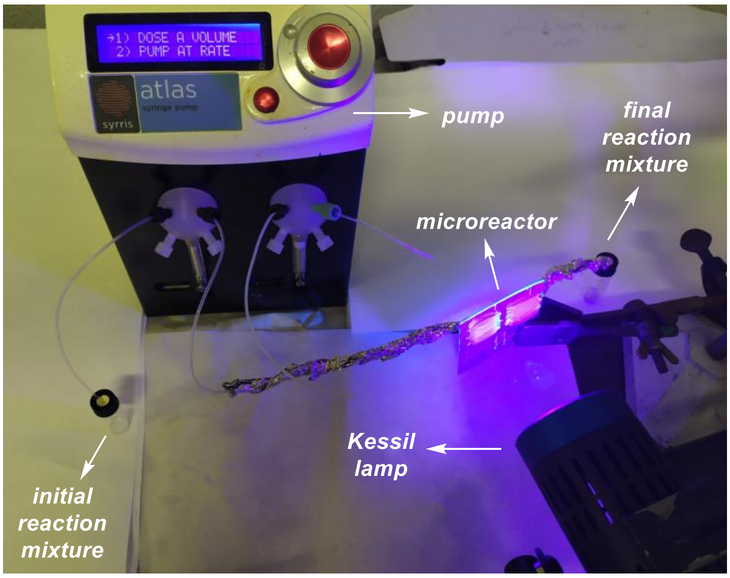

Figure S3. *left:* Assembled microfluidic photoreactor. *right:* Microfluidic photoreactor setup.

B. GENERAL PROCEDURES FOR THE SYNTHESIS OF STARTING MATERIALS

B.1. PREPARATION OF SUBSTITUTED PHENOLS (4m and 4n).

- **N-Boc-tyramine (4m)**

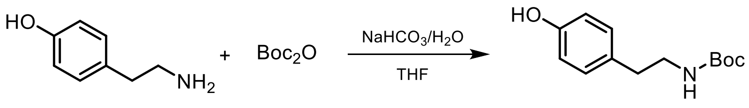

S1 *tyramine* **4m**

Figure S4. N-Boc protection of tyramine.

4m was synthesized following a method previously reported in the literature.[7] The characterization data matched with the reported one.

- **Methyl-(tert-butoxycarbonyl)-L-tyrosinate (4n)**

Boc-Tyr-OH (**S3**)

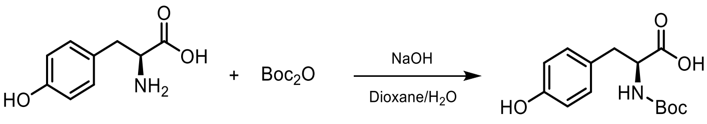

S2 *tyrosine* **S3** ***Boc-Tyr-OH***

Figure S5. N-Boc protection of tyrosine.

Boc-Tyr-OH (**S3**) was synthesized following a method previously reported in the literature.[8] The characterization data matched with the reported one.

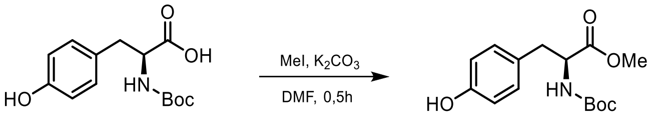

S3 **4n**

Figure S6. Formation of Boc-Tyr-OMe.

4n was synthesized from **S3**, following a method previously reported in the literature.[9] The characterization data matched with the reported one.

B.2. PREPARATION OF α-IODOSULFONES

B.2.1. Synthesis of 5a-d and 5f-h

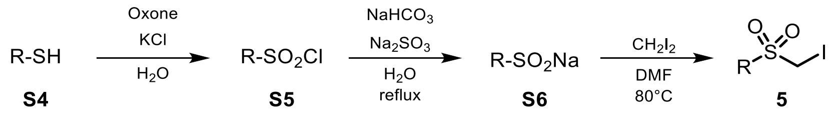

STEP 1, according to a modified literature procedure.[10] A mixture of thiol **S4** (10 mmol, 1 equiv.), Oxone (25 mmol, 2.5 equiv.), KCl (10 mmol, 1 equiv.) and water (30 mL) was vigorously stirred at room temperature for 2 hours. The aqueous phase was extracted with ethyl acetate (4 × 50 mL). The combined organic layer was dried over anhydrous sodium sulfate and concentrated under reduced pressure. The crude product was purified by flash column chromatography (cyclohexane) affording the desired product (R = Cl: 90% yield, R = cyclohexyl: 83% yield). The characterization data matched with the reported one.

STEP 2, according to a literature procedure.[2] The corresponding sulfonyl chloride **S5** (7 mmol, 1 equiv.) was dissolved in water (25 mL). Sodium sulfite (11.2 mmol, 1.6 equiv.) and sodium bicarbonate (11.2 mmol, 1.6 equiv.) were added and the reaction mixture was refluxed for 3 hours in an oil bath. Water was evaporated and ethanol was added to the residue. The suspension was heated for 10 minutes, cooled and filtered. This procedure was repeated twice using the residue of the filtration. The ethanol fractions were combined and the solvent was evaporated under reduced pressure. Sodium sulfinate was used without any further purification (R = F: 93%, R = Cl: 92% yield, R = Br: 88% yield, R = cyclohexyl: 75% yield, R = naphthalenyl: 72% yield). The characterization data matched with the reported one.

STEP 3, according to a literature procedure.[2] A solution of sodium sulfinate **S6** (5 mmol, 1 equiv.) in DMF (20 mL, 0.25 M) was stirred at room temperature for 15 minutes. Diiodomethane (6 mmol, 1.2 equiv.) was added dropwise and the solution was heated up to 80 ºC (heat source: oil bath) and stirring was continued over 17 hours. The reaction was quenched by the addition of water (100 mL). The solution was then transferred to a separatory funnel and extracted with ethyl acetate (3 x 50 mL). The organic phases were combined and washed with brine (50 mL), saturated solution of sodium thiosulfate (50 mL) and then dried over sodium sulfate before concentration in vacuo. The residue was purified by flash column chromatography (cyclohexane/ethyl acetate) to afford the desired α-iodo sulfones **5a-d** and **5f-h**.

Characterization Data

(Iodomethyl)sulfonyl)benzene (5a)

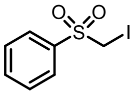

5a

5a was synthesized according to the general procedure **B.2.1** from sodium benzenesulfinate (821 mg, 5 mmol). The final α-iodo sulfone **5a** was obtained as a white solid (1.24 g, 88% yield). **^{1}H-NMR (400 MHz, $CDCl_3$)** δ 7.98 (dt, *J*= 8.5, 1.6 Hz, 2H), 7.74-7.68 (m, 1H), 7.60 (dt, *J*= 8.0, 1.2 Hz, 2H), 4.46 (s, 2H); **^{13}C-NMR (101 MHz, $CDCl_3$)** δ 136.1 (s), 134.7 (s), 129.5 (s), 129.1 (s), 16.8 (s). The characterization data matched with the reported one.[2]

1-((Iodomethyl)sulfonyl)-4-methylbenzene (5b)

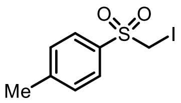

5b

5b was synthesized according to the general procedure **B.2.1** from sodium 4-methylbenzenesulfinate (891 mg, 5 mmol). The final α-iodo sulfone **5b** was obtained as a white solid (1.20 g, 81% yield). **^{1}H NMR (400 MHz, $CDCl_3$)** δ 7.84 (d, *J* = 8.3 Hz, 2H), 7.38 (d, *J* = 7.9 Hz, 2H), 4.44 (s, 2H), 2.46 (s, 3H). The characterization data matched with the reported one.[2]

1-fluoro-4-((iodomethyl)sulfonyl)benzene (5c)

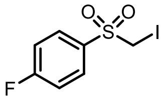

5c

5c was synthesized according to the general procedure **B.2.1** from 4-fluorobenzenesulfonyl chloride (1.36 g, 7 mmol). The final α-iodo sulfone **5c** was obtained as a white solid (1.12 g, 75% yield). **^{1}H-NMR (400 MHz, $CDCl_3$)** δ 8.04-7.95 (m, 2H), 7.32-7.22 (m, 2H), 4.46 (s, 2H); **^{13}C-NMR (101 MHz, $CDCl_3$)** δ 167.6 (s), 165.0 (s), 132.1 (d), 116.9 (d), 16.6 (s); **^{19}F-NMR (376 MHz, $CDCl_3$)** δ -101.70 (m, 1F); **HRMS** (ESI, positive mode) calculated for $C_7H_6FIO_2S$ $[M+Na]^+$: 322.9009, found: 332.9010.

1-chloro-4-((iodomethyl)sulfonyl)benzene (5d)

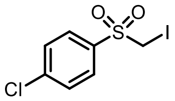

5d

5d was synthesized according to the general procedure **B.2.1** from 4-chlorobenzenesulfonyl chloride (1.48 g, 7 mmol). The final α-iodo sulfone **5d** was obtained as a white solid (807 mg, 51% yield). **^{1}H-NMR (400 MHz, $CDCl_3$)** δ 7.89 (d, *J* = 8.6 Hz, 2H), 7.56 (d, *J* = 8.6 Hz, 2H), 4.46 (s, 2H); **^{13}C-NMR (101 MHz, $CDCl_3$)** δ 141.6 (s), 134.5 (s), 130.6 (s), 129.8 (s), 16.5 (s); **HRMS** (ESI, positive mode) calculated for $C_7H_6ClIO_2S$ $[M+Na]^+$: 338.8714, found: 338.8713.

Iodo(methylsulfonyl)methane (5f)

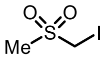

5f

5f was synthesized according to the general procedure **B.2.1** from sodium methanesulfinate (510 mg, 5 mmol). The final α-iodosulfone **5f** was obtained as a white solid (746 mg, 68% yield). **^{1}H NMR (400 MHz, $CDCl_3$)** δ 4.39 (s, 2H), 3.17 (s, 3H). The characterization data matched with the reported one.[2]

((Iodomethyl)sulfonyl)cyclohexane (5g)

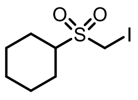

5g

5g was synthesized according to the general procedure **B.2.1** from cyclohexanethiol (1.22 mL, 10 mmol). The final α-iodo sulfone **5g** was obtained as a white solid (504 mg, 35% yield**). ^{1}H-NMR (400 MHz, $CDCl_3$)** δ 4.30 (s, 2H), 3.35 (tt, *J* = 12.1, 3.5 Hz, 1H), 2.13 (dd, *J* = 19.3, 14.6 Hz, 2H), 1.91 (d, *J* = 13.4 Hz, 2H), 1.71 (d, *J* = 12.4 Hz, 1H), 1.55 (qd, *J* = 12.4, 3.2 Hz, 2H), 1.40-1.14 (m, 3H); **^{13}C-NMR (101 MHz, $CDCl_3$)** δ 58.6 (s), 25.3 (s), 25.0 (s), 10.6 (s); **HRMS** (ESI, positive mode) calculated for $C_7H_{13}IO_2S$ $[M+Na]^+$: 310.9573, found: 310.9574. The characterization data matched with the reported one.[11]

1-bromo-4-((iodomethyl)sulfonyl)benzene (5h)

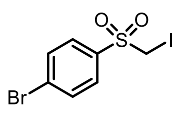

5h

5h was synthesized according to the general procedure **B.2.1** from 4-bromobenzenesulfonyl chloride (1.79 g, 7 mmol). The final α-iodo sulfone **5h** was obtained as a white solid (902 mg, 50% yield**).** **^{1}H-NMR (400 MHz, CDCl$_3$)** δ 7.86–7.81 (m, 2H), 7.77-7.52 (m, 2H), 4.45 (s, 2H); **^{13}C-NMR (101 MHz, CDCl$_3$)** δ 135.1 (s), 132.8 (s), 130.7 (s), 130.3 (s), 15.4 (s); **HRMS** (ESI, positive mode) calculated for $C_7H_6BrIO_2S$ [M+Na]$^+$: 382.8209, found: 382.8215. The characterization data matched with the reported one.[12]

B.2.2. Synthesis of the secondary α-iodo sulfone 5e

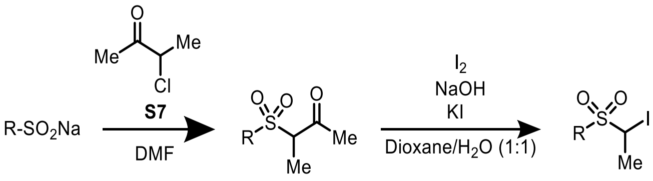

STEP 1, according to a modified literature procedure.[2] To a solution of commercially available 3-chloro butanone **S7** (5 mmol, 1 equiv.) in DMF (10 mL, 0.5 M) was added the sodium sulfinate **S6** (5 mmol, 1 equiv.) in one portion. The reaction mixture was stirred at room temperature for 24 hours. The reaction was quenched by the addition of water (50 mL), the mixture was extracted with ethyl acetate (3 x 35 mL), dried over sodium sulfate and the solvent was removed under reduced pressure. The corresponding adduct was used without any further purification (R = phenyl: 85% yield, R = 4-fluorophenyl: 82% yield).

STEP 2, according to a modified literature procedure.[2] To a dioxane-water (1:1, 0.5 M) solution of the starting material **S8** (2.5 mmol, 1 equiv.) and iodine (10 mmol, 4 equiv.) in the presence of potassium iodide (20 mmol, 8 equiv.), 1 M solution of NaOH is added under stirring at room temperature until decoloration of the excess of iodine occurred. After 20 minutes stirring, the reaction mixture was diluted with water and extracted with DCM (3 x 20 mL). The final α-iodo sulfone **5e**.

Characterization Data

1-fluoro-4-((1-iodoethyl)sulfonyl)benzene (5e)

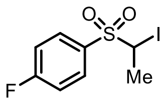

5e

5e was synthesized according to the general procedure **B.2.2** from sodium 4-fluorobenzenesulfinate (911 mg, 5 mmol). The final α-iodo sulfone **5e** was obtained as a white solid (385 mg, 49% yield). **^{1}H NMR (400 MHz, CDCl$_3$)** δ 8.02 – 7.96 (m, 2H), 7.31 – 7.23 (m, 2H), 5.00 (q, J = 7.1 Hz, 1H), 2.10 (d, J = 7.1 Hz, 3H). **^{19}F-NMR (376 MHz, CDCl$_3$)** δ -101.96 (m, 1F). The characterization data matched with the reported one.[2]

C. GENERAL PROCEDURE FOR THE MICROFLUIDIC PHOTOALKYLATION OF PHENOLS

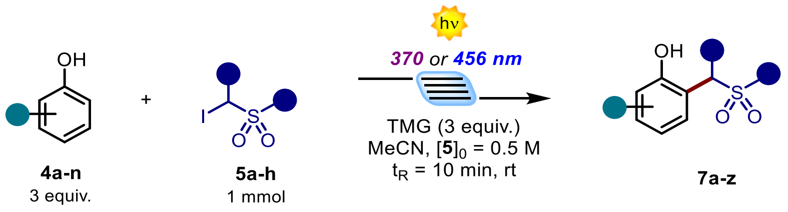

Figure S7. Photochemical alkylation of phenols.

The phenol derivative (**4a-o**) (3 equiv, 3 mmol) and the α-iodosulfone (**5a-h**) (1 equiv, 1 mmol) were introduced into a vial and dissolved in 2 mL of acetonitrile (MeCN), unless otherwise stated. The mixture was degassed with Argon (Ar) for 1 minute. Then, 1,1,3,3-tetramethylguanidine (TMG, 3 equiv, 3 mmol) was added to the vial under Ar atmosphere. The reaction mixture was connected to the flow setup (Figure S3 in Section A.2) and introduced in continuous-flow with a flow rate of 21.3 µL/min (residence time, t_R = 10 min). The crude was collected in a vial connected to the exit of the microreactor (collected volume = 1.5 mL *ca.*). To a 300 µL of the crude were added 2 mL of a saturated solution of ammonium chloride (NH_4Cl), and the organic layer was extracted with dichloromethane (DCM) three times. The organic layer was then separated, dried over magnesium sulfate ($MgSO_4$) and filtered. The solvent was removed under reduced pressure. 300 µL of an internal standard solution (trichloroethylene 0.5 M in $CDCl_3$) were added to the dried crude and the NMR yield was determined.

Another 1 mL of the crude (0.5 mmol, unless otherwise stated) was subsequently extracted as described. The final residue was purified by flash column chromatography on silica gel to afford the *ortho*-alkylated phenols **7a-7aa** as the major regioisomer, in the stated yield. The regioselective ratio was determined by ^{1}H-NMR-analysis of the reaction crude.

Characterization Data

2-((phenylsulfonyl)methyl)phenol (7a)

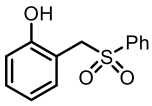

7a

Compound **7a** was synthesized according to the general procedure using phenol **4a** (282.33 mg, 3 mmol, 3 equiv) and ((iodomethyl)sulfonyl)benzene **5a** (282.10 mg, 1 mmol, 1 equiv). 1 mL of the collected crude was purified by flash column chromatography (gradient from 9:1 to 7:3 Hexane/EtOAc) to afford **7a** as a white solid (50% yield, 62.0 mg, 0.250 mmol). Regioselectivity *o:o,o':p* = 10:3:1; total yield = 63%.

Note: The process was scaled-up to 10 mmol scale, obtaining similar results: 1.56 gr of **7a** (63% yield, 6.28 mmol).

^{1}H NMR (400 MHz, $CDCl_3$) δ 7.74 – 7.70 (m, 2H), 7.66 – 7.60 (m, 1H), 7.48 (t, *J* = 7.8 Hz, 2H), 7.24 – 7.19 (m, 1H), 6.95 (d, 1H), 6.81 – 6.76 (m, 2H), 4.45 (s, 2H). **^{13}C NMR (101 MHz, $CDCl_3$)** δ 155.5, 137.3, 134.2, 132.6, 130.9, 129.2 (x2), 128.7 (x2), 121.5, 118.5, 115.8, 58.9. **HRMS (Q-TOF)** calculated for $[C_{13}H_{12}O_3S\text{-}H]^-$: 247.0434, found 247.0414.

5-bromo-2-((phenylsulfonyl)methyl)phenol (**7b**)

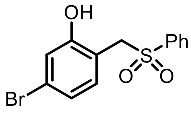

7b

Compound **7b** was synthesized according to the general procedure using 3-bromophenol **4b** (519.03 mg, 3 mmol, 3 equiv) and ((iodomethyl)sulfonyl)benzene **5a** (282.10 mg, 1 mmol, 1 equiv). 1 mL of the collected crude was purified by flash column chromatography (gradient from 9:1 to 7:3 Hexane/EtOAc) to afford **7b** as a white solid (47% yield, 76.8 mg, 0.235 mmol). Regioselectivity *o:o':o,o'* = 6:2.5:1; total yield = 64%.

^{1}H NMR (400 MHz, $CDCl_3$) δ 7.75 (d, *J* = 7.7 Hz, 2H), 7.67 (t, *J* = 7.5 Hz, 1H), 7.52 (t, *J* = 7.4 Hz, 2H), 7.12 (s, 1H), 6.94 (d, *J* = 8.1 Hz, 1H), 6.65 (d, *J* = 8.2 Hz, 1H), 4.39 (s, 2H). **^{13}C NMR (101 MHz, $CDCl_3$)** δ 156.4, 137.2, 134.4, 133.5, 129.4 (x2), 129.0, 128.6 (x2), 124.6, 124.2, 121.7, 114.8, 58.4. **HRMS (Q-TOF)** calculated for $[C_{13}H_{11}BrO_3S\text{-}H]^-$: 324.9540, found 324.9542.

2-((phenylsulfonyl)methyl)-5-(trifluoromethyl)phenol (7c)

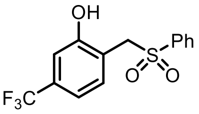

7c

Compound **7c** was synthesized according to the general procedure using 3-(trifluoromethyl)phenol **4c** (486.33 mg, 3 mmol, 3 equiv) and ((iodomethyl)sulfonyl)benzene **5a** (282.10 mg, 1 mmol, 1 equiv). 1 mL of the collected crude was purified by flash column chromatography (gradient from 9:1 to 7:3 Hexane/EtOAc) to afford 100.7 mg of a white solid. The isolated material consisted of a mixture containing **7c** and an inseparable byproduct in a proportion 1:0.1 (**7c** : byproduct; see Figure S8 below). The byproduct arises from the dehalogenation of the starting material **5a**. Corrected yield of product **7c**: 61%. Regioselectivity: *o:p:o,p* = 9:1:1; total yield = 77%.

^{1}H NMR (400 MHz, $CDCl_3$) δ 7.78 – 7.74 (m, 2H), 7.70 – 7.65 (m, 1H), 7.53 (t, 2H), 7.22 (s, 1H), 7.18 (s, 1H), 7.09 – 7.04 (m, 1H), 6.91 (d, *J* = 7.9 Hz, 1H), 4.48 (s, 2H*). Dehalogenated byproduct* CH_3 *signal 3.06 (s, 3H).* **^{13}C NMR (101 MHz, $CDCl_3$)** δ 156.0, 137.1, 134.6, 133.1, 133.0, 129.5 (x2), 128.6 (x2), 122.2, 119.7, 118.2, 115.8, 58.8. **^{19}F NMR decoupled ^{1}H (188 MHz, $CDCl_3$)** δ -63.57. **HRMS (Q-TOF)** calculated for $[C_{14}H_{11}F_3O_3S\text{-}H]^-$: 315.0308, found 315.0316.

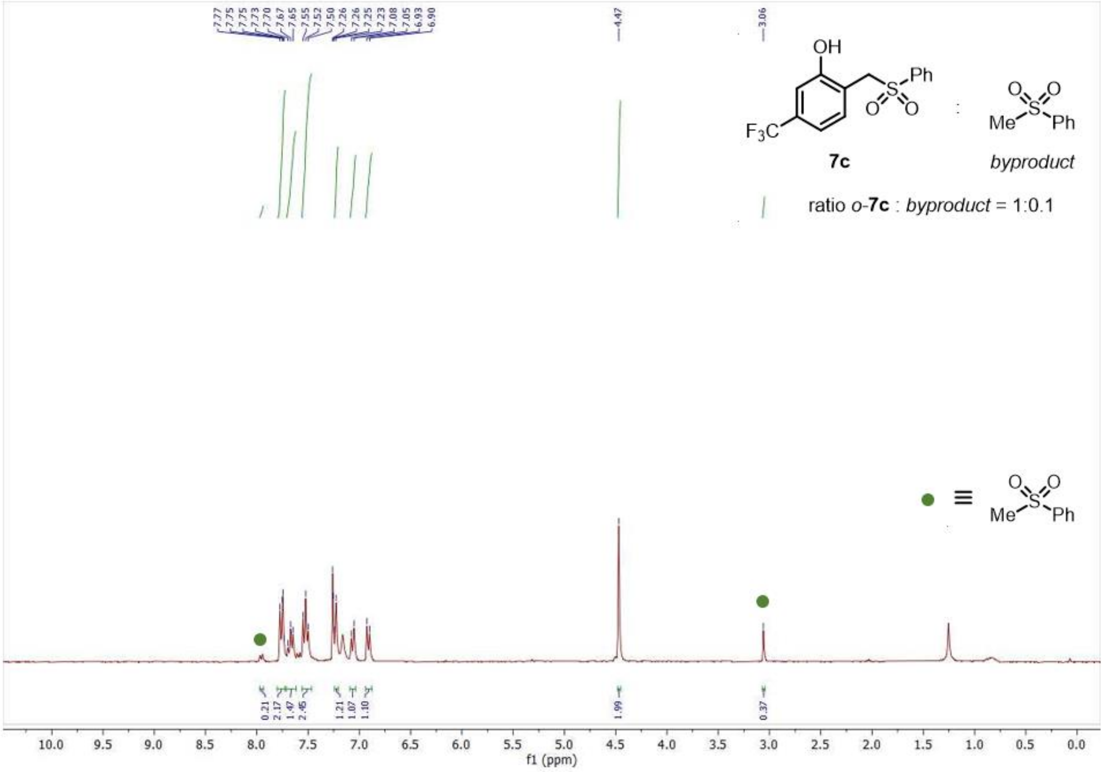

Figure S8. ^{1}H NMR spectrum of the mixture of product **7c** and byproduct (red dots) arising from the dehalogenation of starting material **5a**.

2-((phenylsulfonyl)methyl)-[1,1'-biphenyl]-3-ol (7d)

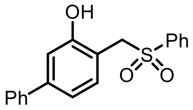

7d

Compound **7d** was synthesized according to the general procedure using [1,1'-biphenyl]-3-ol **4d** (510.63 mg, 3 mmol, 3 equiv) and ((iodomethyl)sulfonyl)benzene **5a** (282.10 mg, 1 mmol, 1 equiv). 1 mL of the collected crude was purified by flash column chromatography (gradient from 9:1 to 7:3 Hexane/EtOAc) to afford 84.3 mg of a white solid. The isolated material consisted of the regioisomer *o*-**7d** containing 15% of the *o'*-**7d** regioisomer. Corrected yield: 45% (72.3 mg of *o*-**7d**). Regioselectivity *o:o':o,o'* = 4:2:1; total yield= 52%.

^{1}H NMR (400 MHz, $CDCl_3$) δ 7.81 – 7.74 (m, 2H), 7.68 – 7.61 (m, 1H), 7.57 – 7.52 (m, 2H), 7.52 – 7.50 (m, 1H), 7.45 – 7.40 (m, 2H), 7.38 – 7.35 (m, 1H), 7.22 (d, *J* = 1.8 Hz, 1H), 7.05 (m, 1H), 6.89 (s, 1H), 6.84 (d, *J* = 7.9 Hz, 1H), 4.48 (s, 2H). **^{13}C NMR (101 MHz, $CDCl_3$)** δ 155.9, 144.1, 140.0, 137.4, 134.3, 132.9, 129.3 (x2), 129.0 (x2), 128.7 (x2), 128.0, 127.1 (x2), 120.3, 117.2, 114.7, 59.0. **HRMS (Q-TOF)** calculated for $[C_{19}H_{16}O_3S\text{-}H]^-$: 323.0747, found 323.0751.

4-(tert-butyl)-2-((phenylsulfonyl)methyl)phenol (7e)

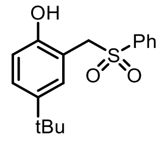

7e

Compound **7e** was synthesized according to the general procedure using 4-(*tert*-butyl)phenol **4e** (496 μL, 3 mmol, 3 equiv) and ((iodomethyl)sulfonyl)benzene **5a** (282.10 mg, 1 mmol, 1 equiv). 1 mL of the collected crude was purified by flash column chromatography (gradient from 9.5:0.5 to 8:2 hexane/EtOAc) to afford **7e** as a white solid (65% yield, 98.8 mg, 0.325 mmol). Regioselectivity *o:o,o'* > 20:1.

^{1}H NMR (400 MHz, CDCl$_3$) δ 7.70 – 7.65 (m, 2H), 7.61 (ddt, *J* = 7.9, 7.1, 1.3 Hz, 1H), 7.50 – 7.42 (m, 2H), 7.22 (dd, *J* = 8.5, 2.5 Hz, 1H), 6.92 (d, *J* = 8.5 Hz, 1H), 6.63 (s, 1H), 6.54 (d, *J* = 2.5 Hz, 1H), 4.43 (s, 2H), 1.09 (s, 9H). **^{13}C NMR (101 MHz, CDCl$_3$)** δ 153.5, 144.7, 137.2, 134.3, 129.6, 129.3 (x2), 129.0 (x2), 128.0, 118.8, 115.7, 59.7, 31.6. **HRMS (Q-TOF)** calculated for $[C_{17}H_{20}O_3S\text{-}H]^-$: 303.1060, found 303.1042.

4-hydroxy-3-((phenylsulfonyl)methyl)benzonitrile (7f)

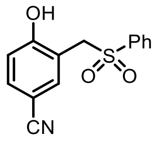

7f

Compound **7f** was synthesized according to the general procedure using 4-hydroxybenzonitrile **4f** (357.36 mg, 3 mmol, 3 equiv) and ((iodomethyl)sulfonyl)benzene **5a** (282.10 mg, 1 mmol, 1 equiv). 0.9 mL of the collected crude was purified by flash column chromatography (gradient from 9:1 to 7:3 Hexane/EtOAc) to afford **7f** as a white solid (67% yield, 82.3 mg, 0.301 mmol). Regioselectivity *o:o,o'* > 20:1; total yield= 67%.

^{1}H NMR (400 MHz, CDCl$_3$) δ 7.78 – 7.75 (m, 2H), 7.74 – 7.68 (m, 1H), 7.59 – 7.51 (m, 3H), 7.11 (d, *J* = 2.0 Hz, 1H), 7.04 (d, *J* = 8.5 Hz, 1H), 4.43 (s, 2H). **^{13}C NMR (101 MHz, CD$_3$OD)** δ 159.6, 136.7, 136.7, 135.0, 134.9, 129.6 (x2), 128.6 (x2), 119.5, 105.0, 104.2, 58.5, 29.9. **HRMS (Q-TOF)** calculated for $[C_{14}H_{11}NO_3S\text{-}H]^-$: 272.0387, found 272.0369.

2-((phenylsulfonyl)methyl)-4-(4,4,6,6-tetramethyl-1,3,2-dioxaborinan-2-yl)phenol (7g)

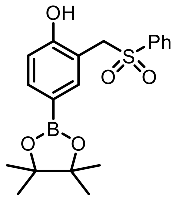

7g

Compound **7g** was synthesized according to the general procedure using 4-(4,4,6,6-tetramethyl-1,3,2-dioxaborinan-2-yl)phenol **4g** (702.30 mg, 3 mmol, 3 equiv) and ((iodomethyl)sulfonyl)benzene **5a** (282.10 mg, 1 mmol, 1 equiv). 1 mL of the collected crude was purified by flash column chromatography (gradient from 9:1 to 7:3 Hexane/EtOAc) to afford **7g** as a white solid (47% yield, 58.1 mg, 0.235 mmol). Regioselectivity *o:o,o'* = 6:1; total yield= 60%.

^{1}H NMR (300 MHz, CDCl$_3$) δ 7.75 – 7.72 (m, 2H), 7.70 – 7.58 (m, 2H), 7.48 (t, *J* = 7.6 Hz, 2H), 7.02 (s, 1H), 6.95 (d, *J* = 8.1 Hz, 1H), 4.43 (s, 2H), 1.29 (s, 12H). **^{13}C NMR (101 MHz, CDCl$_3$)** δ 158.3, 139.9, 137.8, 137.4, 134.2, 129.2 (x2), 128.7 (x2), 118.1, 115.12, 83.9, 59.2, 29.8 (x2), 24.9 (x4). **HRMS (Q-TOF)** calculated for $[C_{19}H_{23}BO_5S\text{-}H]^-$: 373.1286, found 373.1257.

4-hydroxy-3-methoxy-5-((phenylsulfonyl)methyl)benzaldehyde (7h)

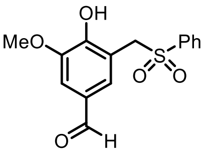

7h

Compound **7h** was synthesized according to the general procedure using 4-hydroxy-3-methoxybenzaldehyde **4h** (456.45 mg, 3 mmol, 3 equiv) and ((iodomethyl)sulfonyl)benzene **5a** (282.10 mg, 1 mmol, 1 equiv). 1 mL of the collected crude was purified by flash column chromatography (gradient from 9:1 to 7:3 Hexane/EtOAc) to afford **7h** as a white solid (75% yield, 114.9 mg, 0.375 mmol).

^{1}H NMR (400 MHz, $CDCl_3$) δ 9.78 (s, 1H), 7.75 – 7.70 (m, 2H), 7.64 – 7.59 (m, 1H), 7.49 – 7.44 (m, 2H), 7.39 – 7.34 (m, 2H), 6.17 (s, 1H), 4.50 (s, 2H), 3.92 (s, 3H). **^{13}C NMR (101 MHz, $CDCl_3$)** δ 190.6, 150.6, 147.2, 138.4, 134.0, 130.2, 129.4, 129.0 (x2), 128.8 (x2), 114.5, 108.5, 56.5, 55.9. **HRMS (Q-TOF)** calculated for $[C_{15}H_{14}O_5S\text{-}H]^-$: 305.0489, found 305.0482.

methyl 5-acetyl-2-hydroxy-3-((phenylsulfonyl)methyl)benzoate (7i)

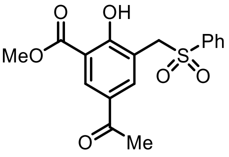

7i

Compound **7i** was synthesized according to the general procedure using methyl 5-acetyl-2-hydroxybenzoate **4i** (582.57 mg, 3 mmol, 3 equiv) and ((iodomethyl)sulfonyl)benzene **5a** (282.10 mg, 1 mmol, 1 equiv). 1.5 mL of the collected crude was purified by flash column chromatography (gradient from 9:1 to 7:3 Hexane/EtOAc) to afford **7i** as a white solid (97% yield, 253.4 mg, 0.727 mmol).

Note: The process was scaled-up to 10 mmol scale, obtaining similar results: 3.38 gr of **7i** (97% yield, 9.7 mmol).

^{1}H-NMR (400 MHz, $CDCl_3$): δ 11.39 (s, 1H), 8.47 (d, *J* = 2.3 Hz, 1H), 8.01 (d, *J* = 2.3 Hz, 1H), 7.75 – 7.69 (m, 2H), 7.65 – 7.59 (m, 1H), 7.51 – 7.44 (m, 2H), 4.51 (s, 2H), 3.96 (s, 3H), 2.55 (s, 3H). **^{13}C-NMR (101 MHz, $CDCl_3$)**: δ 195.5, 170.1, 163.6, 138.4, 138.3, 134.1, 131.9, 129.1 (x2), 128.7 (x2), 128.6, 117.5, 112.4, 55.6, 53.1, 26.4. **HRMS (Q-TOF)** calculated for $[C_{17}H_{16}O_6S+H]^+$: 349.0740, found 349.0721.

2-hydroxy-3-methoxy-5-((phenylsulfonyl)methyl)benzaldehyde (7j)

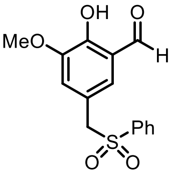

7j

Compound **7j** was synthesized according to the general procedure using 4-hydroxy-3-methoxybenzaldehyde **4j** (456.45 mg, 3 mmol, 3 equiv) and ((iodomethyl)sulfonyl)benzene **5a** (282.10 mg, 1 mmol, 1 equiv). 1 mL of the collected crude was purified by flash column chromatography (gradient from 9:1 to 7:3 Hexane/EtOAc) to afford 116.4 mg of a white solid. The isolated material consisted of a unseparable mixture of the regioisomers *p*-**7j** and *m*-**7j** in a proportion 1.3:1 (*p*-**7j** : *m*-**7j**; see Figure S9 below). Regioselectivity *p*:*m* = 2:1; total yield = 50%. Corrected yield (*p*-**7j**) = 42%.

^{1}H NMR (400 MHz, $CDCl_3$) *major regioisomer (p-7***i***):* δ 11.06 (s, 1H), 9.74 (s, 1H), 7.72 – 7.60 (m, 3H), 7.47 (m, J = 7.6 Hz, 2H), 6.88 (s, 1H), 6.75 (s, J = 2.0 Hz, 1H), 4.25 (s, 2H), 3.73 (s, 3H). *minor regioisomer (m-*7**i**): δ 11.92 (s, 1H), 10.10 (s, 1H), 7.63 – 7.56 (m, 3H), 7.43 (m, 2H), 6.85 (d, 1H), 6.45 (d, J = 8.2 Hz, 1H), 4.56 (s, 2H), 3.84 (s, 3H). **^{13}C NMR (101 MHz, $CDCl_3$)** *major regioisomer (p-*7**i**)*:* δ 196.1, 152.0, 148.4, 137.7, 134.0, 129.2 (x2), 128.6 (x2), 126.7,

124.2, 119.6, 116.5, 62.0, 56.3. *minor regioisomer (m-***7i**): δ 194.7, 153.7, 149.3, 137.5, 134.2, 129.2, 128.5, 121.5, 120.4, 119.6, 118.4, 58.0, 56.2. **HRMS (Q-TOF)** calculated for [$C_{15}H_{14}O_5S$-H]$^-$: 305.0489, found 305.0482.

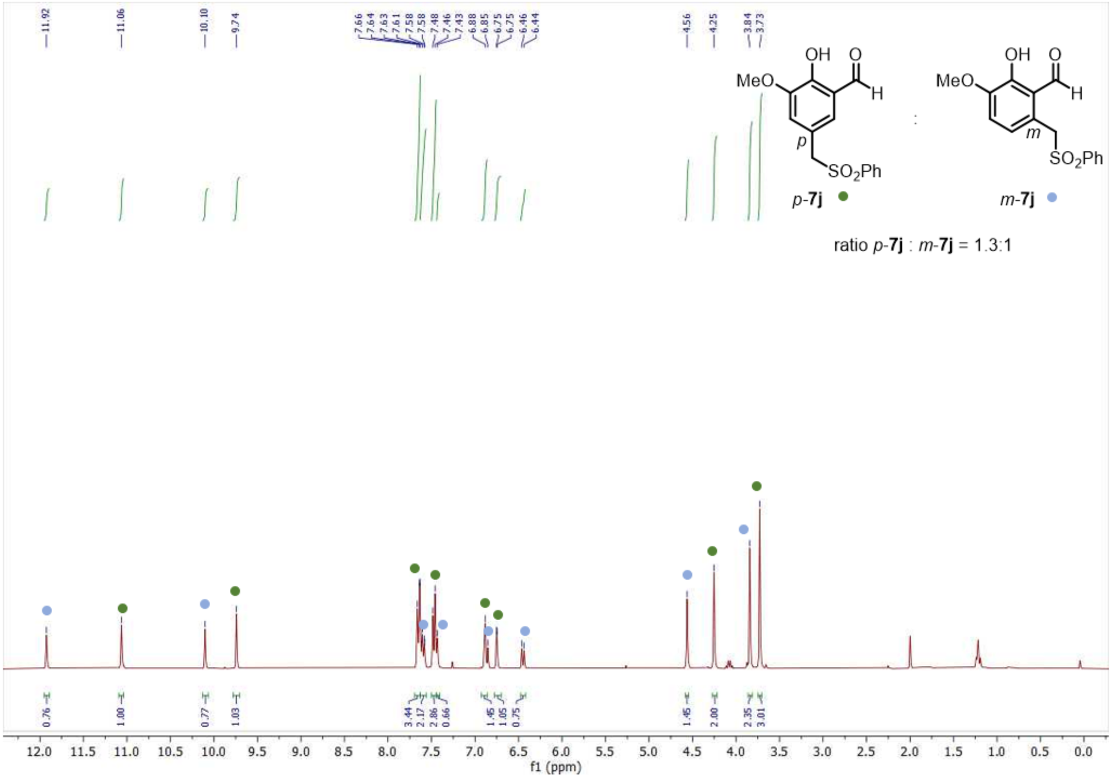

Figure S9. ^{1}H NMR spectrum of the mixture of products *p*-**7j** (green dots) and *m*-**7j** (blue dots).

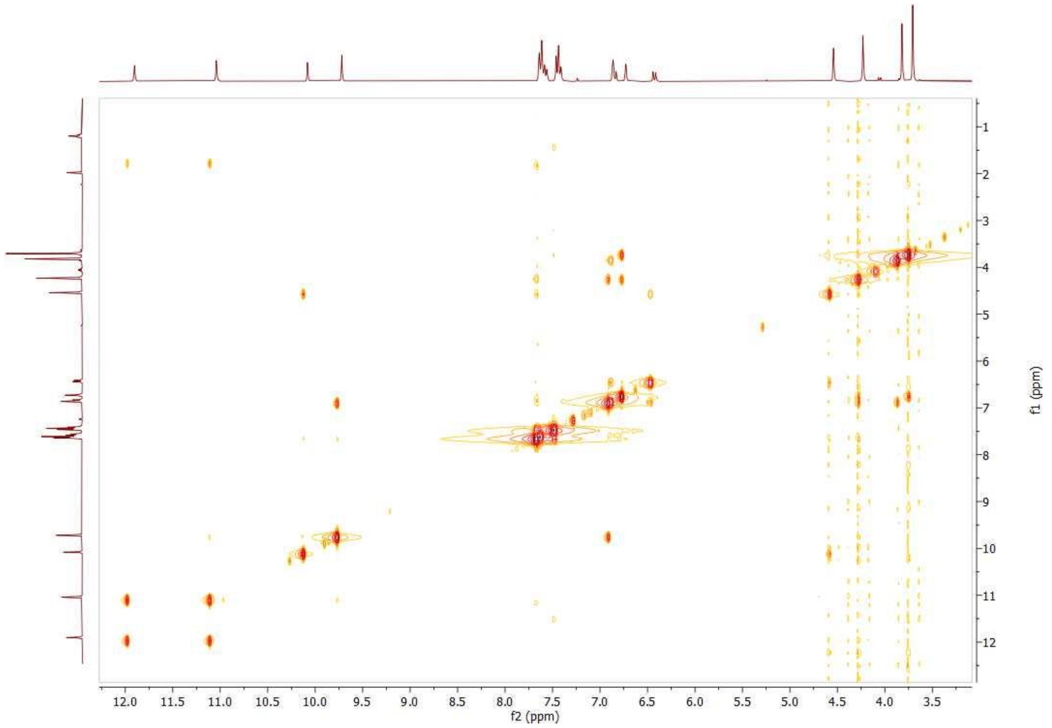

Figure S10. ^{1}H-NOESY NMR spectra of the isolated mixture of regioisomers *p*-**7j** and *m*-**7j**.

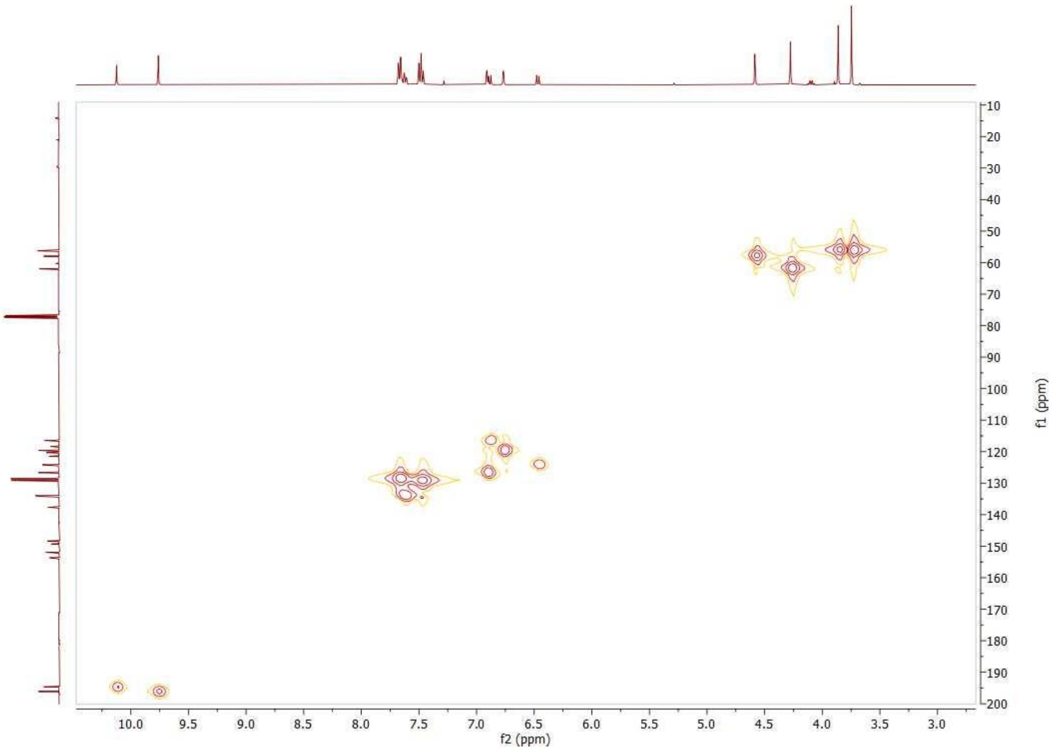

Figure S11. ^{1}H-HMQC NMR spectra of the isolated mixture of regioisomers *p*-**7j** and *m*-**7j**.

2-hydroxy-3-((phenylsulfonyl)methyl)benzaldehyde (7k)

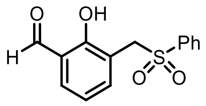

7k

Compound **7k** was synthesized according to the general procedure using 2-hydroxybenzaldehyde **4k** (366.36 mg, 3 mmol, 3 equiv) and ((iodomethyl)sulfonyl)benzene **5a** (282.10 mg, 1 mmol, 1 equiv). 0.9 mL of the collected crude was purified by flash column chromatography (gradient from 9:1 to 7:3 Hexane/EtOAc) to obtain 103.2 mg of a white solid. The isolated material consisted of a mixture containing **7k** and an inseparable byproduct in a proportion 1:0.2 (**7k**:byproduct; see Figure S12 below). The byproduct arises from the dehalogenation of the starting material **5a**. Corrected yield of product **7k**: 75%. Regioselectivity *o:p:o,p* = 8:1.5:1; total yield = 83%.

^{1}H NMR (400 MHz, $CDCl_3$) δ 11.01 (s, 1H), 9.80 (s, 1H), 7.71 – 7.66 (m, 2H), 7.66 – 7.62 (m, 1H), 7.59 – 7.54 (m, 2H), 7.43 (t, *J* = 7.9 Hz, 2H), 7.05 (t, *J* = 7.6 Hz, 1H), 4.48 (s, 2H). *Dehalogenated byproduct -CH_3 signal: 3.06* (s, 3H). **^{13}C NMR (101 MHz, $CDCl_3$)** δ 196.4, 160.0, 139.7, 138.4, 134.8, 133.9, 128.9 (x2), 128.7 (x2), 120.4, 120.0, 117.2, 55.0. *Dehalogenated byproduct -CH_3 signal: 44.6*. **HRMS (Q-TOF)** calculated for $[C_{14}H_{12}O_4S\text{-}H]^-$: 275.0384, found 275.0364.

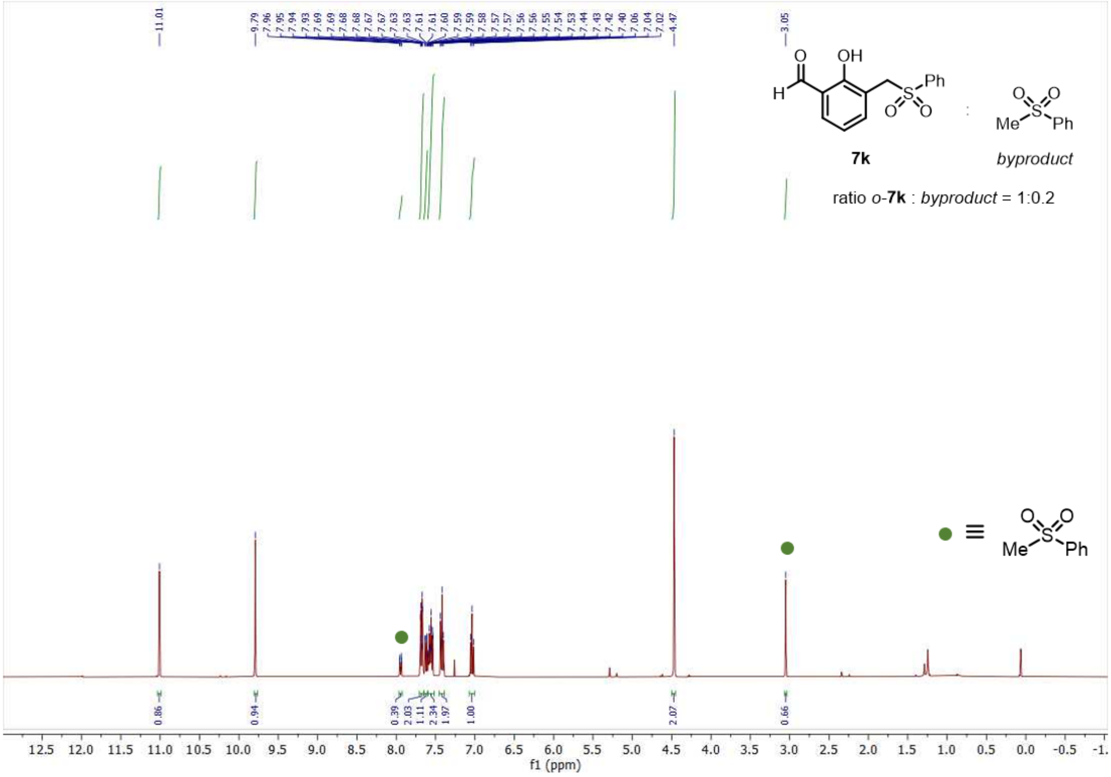

Figure S12. ^{1}H NMR spectrum of the mixture of product **7k** and byproduct (red dots) arising from the dehalogenation of starting material **5a**.

methyl 2-hydroxy-3-((phenylsulfonyl)methyl)benzoate (7l)

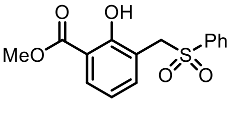

7l

Compound **7l** was synthesized according to the general procedure using methyl 2-hydroxybenzoate **4l** (390.13 μL, 3 mmol, 3 equiv) and ((iodomethyl)sulfonyl)benzene **5a** (282.10 mg, 1 mmol, 1 equiv). 1 mL of the collected crude was purified by flash column chromatography (gradient from 9:1 to 7:3 Hexane/EtOAc) to afford **7l** as a white solid (88% yield, 134.8 mg, 0.440 mmol). Regioselectivity *o:p:o,p* = 11:1.3:1; total yield= 93%.

Note: The process was scaled-up to 10 mmol scale, obtaining similar results: 2.85 gr of **7l** (93% yield, 9.3 mmol).

^{1}H NMR (400 MHz, $CDCl_3$) δ 10.80 (s, 1H), 7.81 (m, 1H), 7.68 (d, *J* = 7.3 Hz, 2H), 7.57 (t, 1H), 7.51 (m, 1H), 7.42 (t, 2H), 6.87 (t, *J* = 7.8 Hz, 1H), 4.48 (s, 2H), 3.90 (s, 3H). **^{13}C NMR (101 MHz, $CDCl_3$)** δ 170.4, 160.0, 138.5, 138.5, 133.8, 131.0, 128.8 (x2), 128.7 (x2), 119.1, 116.9, 112.4, 55.6, 52.6. **HRMS (Q-TOF)** calculated for $[C_{15}H_{14}O_5S\text{-}H]^-$: 305.0489, found 305.0482.

tert-butyl (4-hydroxy-3-((phenylsulfonyl)methyl)phenethyl)carbamate (7m)

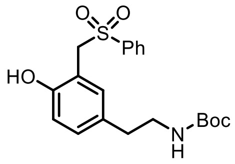

7m

Compound **7m** was synthesized according to the general procedure using tert-butyl (4-hydroxyphenethyl)carbamate **4m** (711.90 mg, 3 mmol, 3 equiv) and ((iodomethyl)sulfonyl)benzene **5a** (282.10 mg, 1 mmol, 1 equiv). 1 mL of the collected crude was purified by flash column chromatography (gradient from 9:1 to 6:4 Hexane/EtOAc) to afford **7m** as a white solid (42% yield, 82.21 mg, 0.210 mmol). Regioselectivity *o:o,o'* = 3:1; total yield = 52%.

^{1}H NMR (400 MHz, CDCl$_3$) δ 7.75 – 7.68 (m, 2H), 7.68 – 7.59 (m, 1H), 7.54 – 7.45 (m, 2H), 7.04 (m, 1H), 6.90 (d, *J* = 8.2 Hz, 1H), 6.55 (d, *J* = 2.2 Hz, 1H), 4.40 (s, 2H), 3.14 (t, *J* = 7.1 Hz, 2H), 2.57 (t, *J* = 7.1 Hz, 2H), 1.44 (s, 9H). **^{13}C NMR (101 MHz, CDCl$_3$)** δ 156.0, 154.2, 137.3, 134.3, 132.6, 132.1, 131.3, 129.2 (x2), 128.7 (x2), 118.9, 116.1, 79.5, 59.1, 42.1, 35.2, 28.6 (x3). **HRMS (Q-TOF)** calculated for $[C_{20}H_{25}NO_5S\text{-}H]^-$: 390.1381, found 390.1353.

tert-butyl (4-hydroxy-3,5-bis((phenylsulfonyl)methyl)phenethyl)carbamate (7ma)

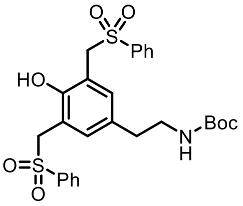

7ma

Compound **7ma** was isolated in a pure form, as a minor regioisomer, during the purification of the major regioisomer **7m** (white solid, 9% yield, 25 mg, 0.05 mmol).

^{1}H NMR (400 MHz, CDCl$_3$) δ 7.71 – 7.66 (m, 4H), 7.66 – 7.60 (m, 2H), 7.50 (t, *J* = 7.8 Hz, 4H), 7.18 (s, 1H), 6.76 (s, 2H), 4.39 (s, 4H), 3.08 (q, *J* = 6.3 Hz, 2H), 2.53 (t, *J* = 7.1 Hz, 2H), 1.44 (s, 9H). **^{13}C NMR (101 MHz, CDCl$_3$)** δ 155.9, 153.2, 137.6 (x2), 134.3 (x2), 133.8 (x2), 132.5, 129.2 (x4), 128.6 (x4), 118.2 (x2), 79.6, 58.1 (x2), 41.8, 35.0, 28.5 (x3). **HRMS (Q-TOF)** calculated for $[C_{27}H_{31}NO_7S_2\text{-}H]^-$: 544.1469, found 544.1494.

methyl (S)-2-((tert-butoxycarbonyl)amino)-3-(4-hydroxy-3-((phenylsulfonyl)methyl)phenyl)propanoate (7n)

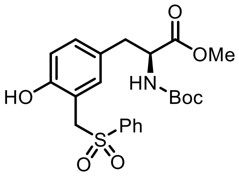

7n

Compound **7n** was synthesized according to the general procedure using methyl (tert-butoxycarbonyl)-L-tyrosinate **4n** (886.02 mg, 3 mmol, 3 equiv) and ((iodomethyl)sulfonyl)benzene **5a** (282.10 mg, 1 mmol, 1 equiv). 1 mL of the collected crude was purified by flash column chromatography (gradient from 9:1 to 7:3 Hexane/EtOAc) to afford **7n** as a white solid (50% yield, 112.4 mg, 0.250 mmol). Regioselectivity *o:o,o'* = 3:1; total yield = 94%.

^{1}H NMR (400 MHz, CDCl$_3$) δ 7.70 (d, *J* = 6.9 Hz, 2H), 7.61 (t, *J* = 7.5 Hz, 1H), 7.47 (t, *J* = 7.7 Hz, 2H), 6.94 (d, *J* = 6.2 Hz, 1H), 6.77 (d, *J* = 8.2 Hz, 1H), 6.68 (s, 1H), 4.94 (d, *J* = 8.2 Hz, 1H), 4.38 (s, 2H), 3.69 (s, 3H), 2.97 – 2.77 (m, 2H), 1.41 (s, 9H). **^{13}C NMR (101 MHz, CDCl$_3$)** 172.3, 155.2, 154.7, 137.6, 134.2, 133.3, 131.7, 129.2 (x2), 128.7 (x2), 118.3, 115.9, 58.6, 54.7, 52.5, 37.5, 28.4 (x3). **HRMS (Q-TOF)** calculated for $[C_{22}H_{27}NO_7S\text{-}H]^-$: 448.1435, found 448.1411.

methyl (S)-2-((tert-butoxycarbonyl)amino)-3-(4-hydroxy-3,5-bis((phenylsulfonyl)methyl)phenyl)propanoate (7na)

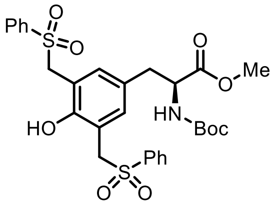

7na

Compound **7na** was isolated in a pure form, as a minor regioisomer, during the purification of the major regioisomer **7n** (white solid, 9% yield, 28 mg, 0.05 mmol).

^{1}H NMR (400 MHz, $CDCl_3$) δ 7.74 – 7.59 (m, 6H), 7.52 (t, J = 7.6 Hz, 4H), 7.21 (s, 1H), 6.80 (s, 2H), 4.85 (d, J = 8.0 Hz, 1H), 4.36 (s, 4H), 3.75 (s, 2H), 2.86 (m, 2H), 1.43 (s, 9H). **^{13}C NMR (101 MHz, $CDCl_3$)** δ 155.1, 153.6, 137.6 (x2), 134.4 (x2), 134.4 (x2), 129.3 (x4), 128.6 (x4), 118.2 (x2), 80.3, 58.0 (x2), 54.6, 52.7 (x2), 37.3, 28.5 (x3). **HRMS (Q-TOF)** calculated for $[C_{29}H_{33}NO_9S_2\text{-}H]^-$: 602.1524, found 602.1523.

N-(4-hydroxy-3-((phenylsulfonyl)methyl)phenyl)acetamide (7o)

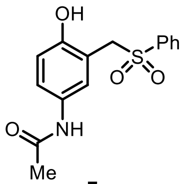

7o

Compound **7o** was synthesized according to the general procedure using N-(4-hydroxyphenyl)acetamide **4o** (226.76 mg, 1.5 mmol, 3 equiv), ((iodomethyl)sulfonyl)benzene **5a** (141.05 mg, 0.5 mmol, 1 equiv), and a 4:1 mixture of MeCN:DMSO as solvent system (V_{total}= 1 mL). 0.45 mL of the collected crude was purified by flash column chromatography (gradient from 95:5 to 90:10 DCM/MeOH) to afford **7o** as a white solid (43% yield, 29.5 mg, 0.097 mmol). Regioselectivity *o:o,o'* = 7:1; total yield = 50%.

^{1}H NMR (300 MHz, DMSO-d_6) δ 9.71 (s, 1H), 9.26 (s, 1H), 7.77 – 7.63 (m, 3H), 7.55 (dd, J = 8.5, 6.9 Hz, 2H), 7.42 – 7.35 (m, 2H), 6.68 – 6.58 (m, 1H), 4.48 (s, 2H), 1.98 (s, 3H). **^{13}C NMR (75 MHz, DMSO-d_6)** δ 167.6, 151.9, 139.1, 133.6, 130.9, 129.0 (x2), 127.9 (x2), 123.1, 121.3, 114.9, 114.4, 55.3, 23.7. **HRMS (Q-TOF)** calculated for $[C_{15}H_{15}NO_4S\text{-}H]^-$: 304.0649, found 304.0658.

2-(tosylmethyl)phenol (7p)

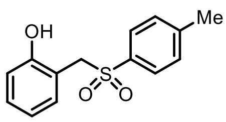

7p

Compound **7p** was synthesized according to the general procedure using phenol **4a** (282.33 mg, 3 mmol, 3 equiv) and 1-((iodomethyl)sulfonyl)-4-methylbenzene **5b** (296.12 mg, 1 mmol, 1 equiv). 1 mL of the collected crude was purified by flash column chromatography (gradient from 9:1 to 7:3 Hexane/EtOAc) to afford **7p** as a white solid (54% yield, 70.8 mg, 0.270 mmol). Regioselectivity *o:p:o,o'* = 6:2:1; total yield = 65%.

^{1}H NMR (400 MHz, $CDCl_3$) δ 7.60 (d, J = 8.3 Hz, 2H), 7.27 (d, J = 8.1 Hz, 2H), 7.24 – 7.17 (m, 1H), 6.95 (d, J = 8.1 Hz, 1H), 6.81 – 6.76 (m, 2H), 4.42 (s, 2H), 2.42 (s, 3H). **^{13}C NMR (101 MHz, $CDCl_3$)** δ 155.7, 145.4, 134.3, 132.6, 130.9, 129.8 (x2), 128.7 (x2), 121.5, 118.8, 116.0, 59.2, 21.8. **HRMS (Q-TOF)** calculated for $[C_{14}H_{14}O_3S\text{-}H]^-$: 261.0591, found 261.0594.

2-(((4-fluorophenyl)sulfonyl)methyl)phenol (7q)

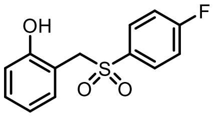

7q

Compound **7q** was synthesized according to the general procedure using phenol **4a** (282.33 mg, 3 mmol, 3 equiv) and 1-fluoro-4-((iodomethyl)sulfonyl)benzene **5c** (300.09 mg, 1 mmol, 1 equiv). 1 mL of the collected crude was purified by flash column chromatography (gradient from 9:1 to 7:3 Hexane/EtOAc) to afford **7q** as a white solid (53% yield, 70.6 mg, 0.265 mmol). Regioselectivity *o:p:o,o'* = 4:1.4:1; total yield = 67%.

^{1}H NMR (400 MHz, CDCl$_3$) δ 7.74 – 7.65 (m, 2H), 7.25 – 7.18 (m, 1H), 7.13 (t, *J* = 8.5 Hz, 2H), 6.89 (d, 1H), 6.86 – 6.78 (m, 2H), 6.58 (s, 1H), 4.45 (s, 2H). **^{13}C NMR (101 MHz, CDCl$_3$)** δ 167.4, 164.9, 155.4, 132.5, 131.7, 131.6, 131.0, 121.5, 118.1, 116.6, 116.3, 115.5, 58.7. **^{19}F NMR decoupled ^{1}H (188 MHz, CDCl$_3$)** δ -103.25. **HRMS (Q-TOF)** calculated for $[C_{13}H_{11}FO_3S\text{-}H]^-$: 265.0340, found 265.0333.

2-(((4-chlorophenyl)sulfonyl)methyl)phenol (7r)

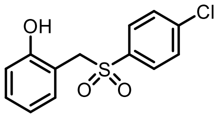

7r

Compound **7r** was synthesized according to the general procedure using phenol **4a** (390.13 μL, 3 mmol, 3 equiv) and 1-chloro-4-((1-iodoethyl)sulfonyl)benzene **5d** (314.11 mg, 1 mmol, 1 equiv). 1 mL of the collected crude was purified by flash column chromatography (gradient from 9:1 to 7:3 Hexane/EtOAc) to afford 70.6 mg of a white solid. The isolated material consisted of a mixture containing **7r** and an inseparable byproduct in a proportion 1:0.3 (**7r**:byproduct; see Figure S13 below). The byproduct arises from the dehalogenation of the starting material **5d**. Corrected yield of product **7r**: 42%. Regioselectivity: *o:p:o,p* = 4:1:1; total yield = 50%

^{1}H NMR (400 MHz, CDCl$_3$) δ 7.62 (d, *J* = 8.6 Hz, 2H), 7.44 (d, *J* = 8.6 Hz, 2H), 7.25 – 7.21 (m, 1H), 6.92 (d, *J* = 8.1 Hz, 1H), 6.87 – 6.80 (m, 2H), 4.44 (s, 2H). **^{13}C NMR (101 MHz, CDCl$_3$)** δ 155.3, 140.9, 132.5, 131.0, 130.1(x2), 129.8, 129.4 (x2), 129.0, 121.7, 118.3, 58.7. **HRMS (Q-TOF)** calculated for $[C_{13}H_{10}ClO_3S\text{-}H]^-$ 281.0045, found 281.0026

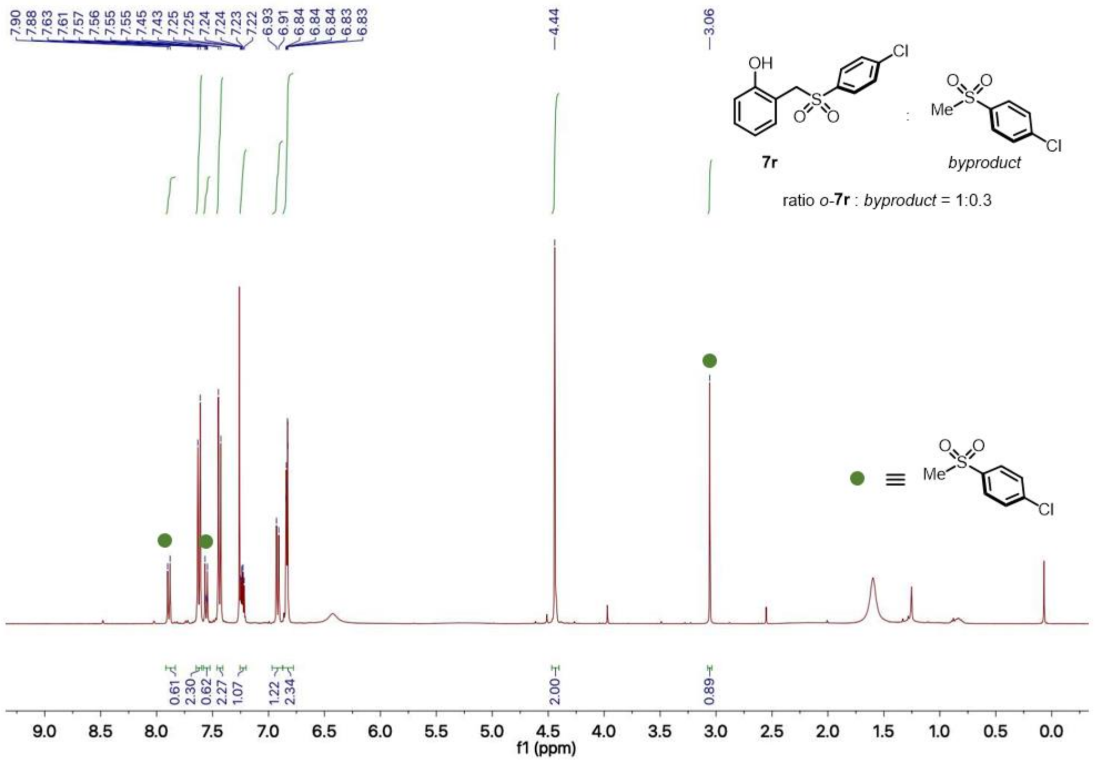

Figure S13. NMR spectrum of the mixture of product **7r** and byproduct (red dots) arising from the dehalogenation of starting material **5d**.

2-(1-((4-fluorophenyl)sulfonyl)ethyl)phenol (7s)

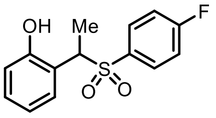

7s

Compound **7s** was synthesized according to the general procedure using phenol **4a** (282.33 mg, 3 mmol, 3 equiv) and 1-fluoro-4-((1-iodoethyl)sulfonyl)benzene **5e** (314.11 mg, 1 mmol, 1 equiv). 1 mL of the collected crude was purified by flash column chromatography (gradient from 9:1 to 7:3 Hexane/EtOAc) to afford **7s** as a white solid (25% yield, 35.0 mg, 0.125 mmol). Regioselectivity >20:1.

^{1}H NMR (400 MHz, $CDCl_3$) δ 7.64 – 7.58 (m, 2H), 7.23 – 7.17 (m, 1H), 7.14 – 7.05 (m, 2H), 6.96 – 6.93 (m, 1H), 6.90 – 6.83 (m, 2H), 6.49 (s, 1H), 4.75 (q, J = 7.3 Hz, 1H), 1.73 (d, J = 7.3 Hz, 3H). **^{13}C NMR (101 MHz, $CDCl_3$)** δ 167.4, 164.8, 155.0, 132.3, 132.2, 130.5, 129.4, 121.6, 121.1, 118.4, 116.3, 116.1, 60.7, 13.5. **^{19}F NMR decoupled ^{1}H (188 MHz, $CDCl_3$)** δ 103.12 **HRMS (Q-TOF)** calculated for $[C_{14}H_{13}FO_3S\text{-}H]^-$: 279.0479, found 279.0498.

2-((methylsulfonyl)methyl)phenol (7t)

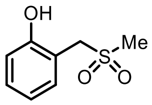

7t

Compound **7t** was synthesized according to the general procedure using phenol **4a** (282.33 mg, 3 mmol, 3 equiv) and iodo(methylsulfonyl)methane **5f** (220.02 mg, 1 mmol, 1 equiv). 0.55 mL of the collected crude was purified by flash column chromatography (gradient from 9:1 to 7:3 Hexane/EtOAc) to afford **7t** as a white solid (42% yield, 21.5 mg, 0.115 mmol). Regioselectivity *o:p:o,o'* = 6:2:1; total yield = 50%.

^{1}H NMR (400 MHz, CDCl$_3$) δ 7.33 – 7.28 (m, 1H), 7.26 – 7.22 (m, 1H), 7.03 – 6.96 (m, 2H), 6.57 (s, 1H), 4.37 (s, 2H), 2.86 (s, 3H). **^{13}C NMR (101 MHz, CDCl$_3$)** δ 155.1, 132.5, 131.3, 122.1, 118.5, 115.8, 57.2, 39.2. **HRMS (Q-TOF)** calculated for $[C_8H_{10}O_3S\text{-}H]^-$: 185.0278, found 185.0269.

2-((cyclohexylsulfonyl)methyl)phenol (7u)

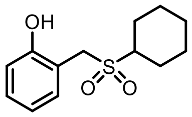

7u

Compound **7u** was synthesized according to the general procedure using phenol **4a** (282.33 mg, 3 mmol, 3 equiv) and ((iodomethyl)sulfonyl)cyclohexane **5g** (146.15 mg, 1 mmol, 1 equiv). 1 mL of the collected crude was purified by flash column chromatography (gradient from 9:1 to 7:3 Hexane/EtOAc) to afford **7u** as a white solid (42% yield, 53.4 mg, 0.21 mmol). Regioselectivity > 20:1.

^{1}H NMR (400 MHz, CDCl$_3$) δ 7.36 – 7.28 (m, 1H), 7.19 (dq, *J* = 7.0, 1.8 Hz, 1H), 7.00 (ddd, *J* = 19.8, 7.9, 3.4 Hz, 2H), 4.33 (s, 2H), 2.91 (tt, *J* = 12.2, 3.8 Hz, 1H), 2.18 (dd, *J* = 10.6, 6.0 Hz, 2H), 1.93 (d, *J* = 7.3 Hz, 2H), 1.77 – 1.69 (m, 2H), 1.68 – 1.52 (m, 4H).**^{13}C NMR (101 MHz, CDCl$_3$)** δ 155.8, 132.3, 131.1, 121.9, 119.2, 115.6, 59.4, 53.1, 25.1, 25.1 (x4). **HRMS (Q-TOF)** calculated for $[C_{13}H_{17}O_3S\text{-}H]^-$: 253.0904 found 253.0889

methyl 2-hydroxy-3-(tosylmethyl)benzoate (7v)

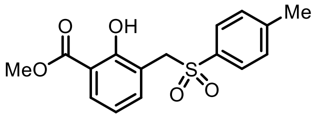

7v

Compound **7v** was synthesized according to the general procedure using 2-hydroxybenzoate **4l** (390.13 μL, 3 mmol, 3 equiv) and 1-((iodomethyl)sulfonyl)-4-methylbenzene **5b** (296.12 mg, 1 mmol, 1 equiv). 1 mL of the collected crude was purified by flash column chromatography (gradient from 9:1 to 7:3 Hexane/EtOAc) to afford **7v** as a white solid (73% yield, 116.9 mg, 0.365 mmol). Regioselectivity *o:o,p:p* = 8:1.4:1; total yield = 85%.

^{1}H NMR (400 MHz, CDCl$_3$) δ 10.83 (s, 1H), 7.81 (m, 1H), 7.56 (d, *J* = 8.3 Hz, 2H), 7.50 (m, 1H), 7.21 (d, *J* = 8.0 Hz, 2H), 6.86 (t, *J* = 7.7 Hz, 1H), 4.46 (s, 2H), 3.90 (s, 3H), 2.39 (s, 3H). **^{13}C NMR (101 MHz, CDCl$_3$)** δ 170.4, 160.1, 144.7, 138.5, 135.7, 130.9, 129.5 (x2), 128.7 (x2), 119.0, 117.0, 112.4, 55.6, 52.6, 21.7. **HRMS (Q-TOF)** calculated for $[C_{16}H_{16}O_5S\text{-}H]^-$: 319.0646, found 319.0626.

methyl 3-(((4-fluorophenyl)sulfonyl)methyl)-2-hydroxybenzoate (7w)

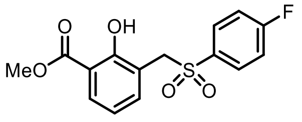

7w

Compound **7w** was synthesized according to the general procedure using 2-hydroxybenzoate **4l** (390.13 μL, 3 mmol, 3 equiv) and 1-fluoro-4-((iodomethyl)sulfonyl)benzene **5c** (300.09 mg, 1 mmol, 1 equiv). 1 mL of the collected crude was purified by flash column chromatography (gradient from 9:1 to 7:3 Hexane/EtOAc) to afford **7w** as a white solid (60% yield, 97.2 mg, 0.305 mmol). Regioselectivity *o:o,p:p* = 8:1.8:1; total yield = 79%.

^{1}H NMR (400 MHz, CDCl$_3$) δ 10.82 (s, 1H), 7.82 (m, 1H), 7.71 – 7.64 (m, 2H), 7.54 (m, 1H), 7.08 (t, *J* = 8.6 Hz, 2H), 6.89 (t, *J* = 7.8 Hz, 1H), 4.47 (s, 2H), 3.91 (s, 3H). **^{13}C NMR (101 MHz, CDCl$_3$)** δ 170.5, 167.2, 164.7, 159.9, 138.5, 134.5, 131.7, 131.1, 119.2, 116.8, 116.2, 116.0, 112.5, 55.7, 52.7. **^{19}F NMR (188 MHz, CDCl$_3$) decoupled ^{1}H** δ -104.21. **HRMS (Q-TOF)** calculated for $[C_{15}H_{13}FO_5S\text{-}H]^-$: 323.0395, found 323.0383.

methyl 3-(((4-chlorophenyl)sulfonyl)methyl)-2-hydroxybenzoate (7x)

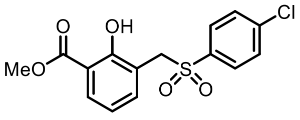

7x

Compound **7x** was synthesized according to the general procedure using 2-hydroxybenzoate **4l** (390.13 μL, 3 mmol, 3 equiv) and 1-chloro-4-((iodomethyl)sulfonyl)benzene **5d** (316.54 mg, 1 mmol, 1 equiv). 1 mL of the collected crude was purified by flash column chromatography (gradient from 9:1 to 7:3 Hexane/EtOAc) to afford **7x** as a white solid (52% yield, 88.6 mg, 0.261 mmol). Regioselectivity *o:o,p:p* = 8:1:1; total yield = 64%.

^{1}H NMR (500 MHz, CDCl$_3$) δ 10.8 (s, 1H), 7.8 (dd, *J* = 8.0, 1.7 Hz, 1H), 7.6 – 7.6 (m, 2H), 7.5 (dd, *J* = 7.6, 1.7 Hz, 1H), 7.4 – 7.3 (m, 2H), 6.9 (t, *J* = 7.7 Hz, 1H), 4.5 (s, 2H), 3.9 (s, 3H). **^{13}C NMR (101 MHz, CDCl$_3$)** δ 170.2, 159.8, 140.4, 138.3, 136.9, 131.0, 130.1 (x2), 129.0 (x2), 119.1, 116.5, 112.4, 55.5, 52.6. **HRMS (Q-TOF)** calculated for $[C_{15}H_{12}ClO_5S\text{-}H]^-$: 339.0099, found 339.0088

methyl 3-(((4-bromophenyl)sulfonyl)methyl)-2-hydroxybenzoate (7y)

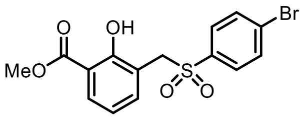

7y

Compound **7y** was synthesized according to the general procedure using 2-hydroxybenzoate **4l** (390.13 μL, 3 mmol, 3 equiv) and 1-bromo-4-((iodomethyl)sulfonyl)benzene **5h** (360.99 mg, 1 mmol, 1 equiv). 1 mL of the collected crude was purified by flash column chromatography (gradient from 9:1 to 7:3 Hexane/EtOAc) to afford **7y** as a white solid (42% yield, 80.9 mg, 0.213 mmol). Regioselectivity *o:o,p* = 6:1.

^{1}H NMR (**400 MHz, CDCl$_3$**) δ 10.85 (s, 1H), 7.83 (dd, *J* = 8.0, 1.7 Hz, 1H), 7.59 – 7.51 (m, 5H), 6.90 (t, *J* = 7.8 Hz, 1H), 4.49 (s, 2H), 3.93 (s, 2H). **^{13}C NMR** (**101 MHz, CDCl$_3$**) δ 170.2, 159.8, 138.3, 137.5, 132.0, 131.0, 130.2, 129.0, 119.1, 116.5, 112.4, 55.5, 52.6. **HRMS (Q-TOF)** calculated for $[C_{15}H_{13}BrO_5S\text{-}H]^-$: 382.9569, found 382.9587.

methyl (R)-3-(1-((4-fluorophenyl)sulfonyl)ethyl)-2-hydroxybenzoate (7z)

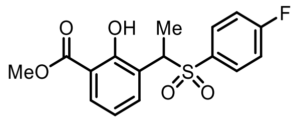

7z

Compound **7z** was synthesized according to the general procedure using 2-hydroxybenzoate **4l** (390.13 μL, 3 mmol, 3 equiv) and 1-fluoro-4-((1-iodoethyl)sulfonyl)benzene **5e** (314.11 mg, 1 mmol, 1 equiv). 1 mL of the collected crude was purified by flash column chromatography (gradient from 9:1 to 7:3 Hexane/EtOAc) to afford **7z** as a white solid (30% yield, 50.75 mg, 0.150 mmol). Regioselectivity > 20:1.

^{1}H NMR (400 MHz, CDCl$_3$) δ 10.91 (s, 1H), 7.79 (m, 1H), 7.71 (m, 1H), 7.65 – 7.59 (m, 2H), 7.03 (t, *J* = 8.6 Hz, 2H), 6.93 (t, *J* = 7.8 Hz, 1H), 5.01 (q, *J* = 7.2 Hz, 1H), 3.90 (s, 3H), 1.75 (d, *J* = 7.2 Hz, 3H). **^{13}C NMR (101 MHz, CDCl$_3$)** δ 170.5, 167.1, 164.5, 159.4, 135.3, 133.9, 132.0, 130.7, 122.6, 119.2, 116.0, 115.7, 112.1, 56.4, 52.7, 13.6. **^{19}F NMR decoupled ^{1}H (188 MHz, CDCl$_3$)** δ -104.73 **HRMS (Q-TOF)** calculated for [$C_{16}H_{15}FO_5S$-H]$^-$: 337.0551, found 337.0533.

methyl 3-((cyclohexylsulfonyl)methyl)-2-hydroxybenzoate (7aa)

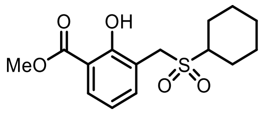

7aa

Compound **7aa** was synthesized according to the general procedure using 2-hydroxybenzoate **4l** (390.13 μL, 3 mmol, 3 equiv) and ((iodomethyl)sulfonyl)cyclohexane **5g** (288.14 mg, 1 mmol, 1 equiv). 1 mL of the collected crude was purified by flash column chromatography (gradient from 9:1 to 7:3 Hexane/EtOAc) to afford **7aa** as a white solid (48% yield, 74.9 mg, 0.24 mmol). Regioselectivity > 20:1.

^{1}H NMR (400 MHz, CDCl$_3$) δ 11.35 (s, 1H), 7.87 (dd, *J* = 8.0, 1.7 Hz, 1H), 7.66 (dd, *J* = 7.7, 1.7 Hz, 1H), 6.93 (t, *J* = 7.8 Hz, 1H), 4.32 (s, 2H), 3.96 (s, 3H), 2.76 (tt, *J* = 12.1, 3.4 Hz, 1H), 2.26 – 2.17 (m, 2H), 1.89 (q, *J* = 4.0 Hz, 2H), 1.73 – 1.64 (m, 1H), 1.54 (qd, *J* = 11.6, 11.2, 5.7 Hz, 2H), 1.30 – 1.14 (m, 3H). **^{13}C NMR (101 MHz, CDCl$_3$)** δ 170.7, 159.6, 139.1, 130.9, 119.5, 117.1, 112.7, 60.1, 52.7, 49.5, 25.2 (x2), 25.2, 25.1(x2). **HRMS(Q-TOF)** calculated for [$C_{15}H_{19}O_5S$-H]$^-$: 311.0959, found 311.0941

D. OPTIMIZATION OF THE REACTION CONDITIONS

Table S1: Comparison of the reaction in flow vs in-batch.

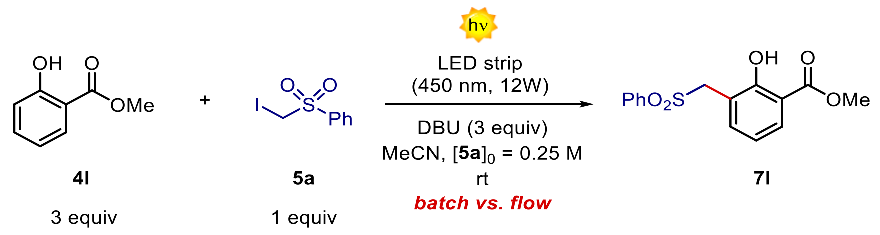

Entry	light source	t_R (min)	NMR yield 7l (%)
**1**	**Batch** - LED strip 450 nm (12 W)	1080 (18 h)	75 (66)[a]
**2**	LED strip 450 nm (12 W)	45	75 (67)[a]
**3**	LED strip 450 nm (12 W)	10	16

[a]isolated yield of **7l** after flash column chromatography.

Table S2: Effect of the light source power and retention time (t_R)

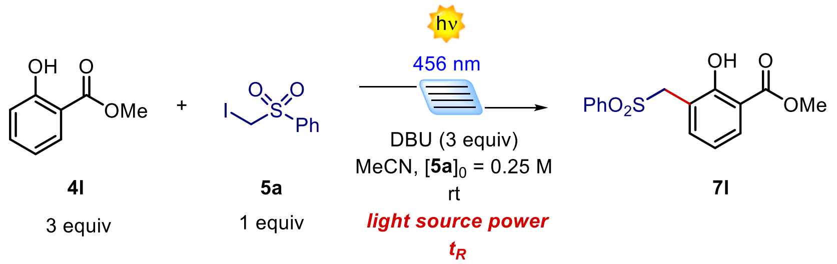

Entry	light source	t_R (min)	NMR yield 7l (%)	r.s.m. 5a (%)[b]
**1**	Kessil 456 nm (180 W) 100%	20	5	0
**2**	Kessil 456 nm (180 W) 75%	20	16	0
**3**	Kessil 456 nm (180 W) 50%	20	20	0
**4**	Kessil 456 nm (180 W) 100%	10	32	0
**5**	Kessil 456 nm (180 W) 75%	10	33	0
**6**	Kessil 456 nm (180 W) 50%	10	34	0
**7**	Kessil 456 nm (180 W) 25%	10	42	4

[b]r.s.m.: remaining starting material.

Table S3: Effect of the concentration and stoichiometry

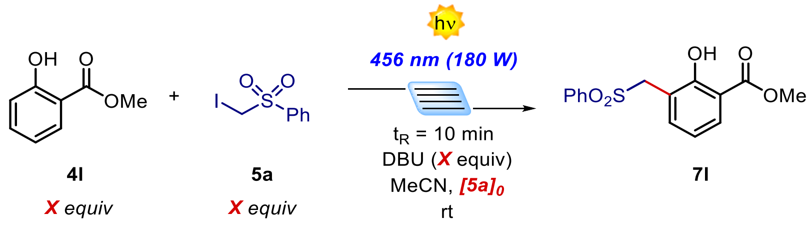

Entry	[5a]₀, M	ratio 4l:DBU:5a	NMR yield 7l (%)	r.s.m. 5a (%)[a]
**1**	0.250	3:3:1	42	4
**2**	0.500	3:3:1	48	0
**3**	0.500	3:1.5:1	40	20
**4**	0.500	2:2:1	26	40
**5**	0.500	1:1:2	18	>50

[a]r.s.m.: remaining starting material.

Table S4: Effect of the base

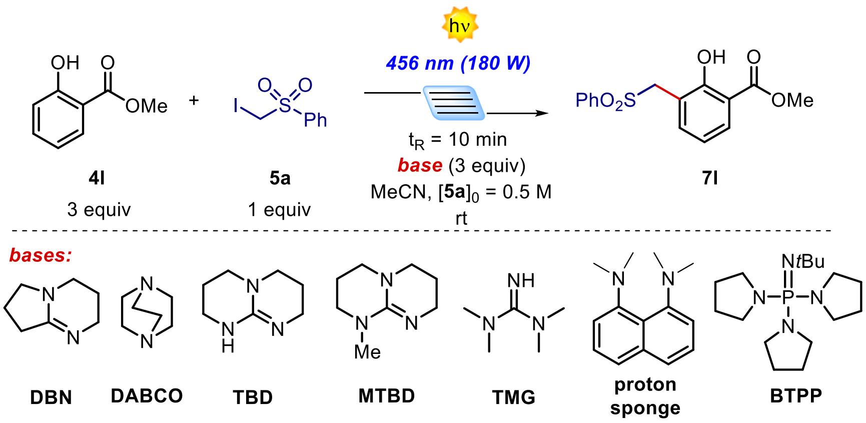

Entry	base	pKa (in MeCN)	ratio 4l:base:5a	NMR yield 7l (%)	r.s.m. 5a %[a]
**1**	DBN	23.4	3:3:1	0	>50
**2**	DABCO	2.97, 8.93[b]	3:3:1	0	>50
**3**	TBD	26.0	3:3:1	58	0
**4**	MTBD	25.4	3:3:1	26	0
**5**	TMG	23.3	3:3:1	93	0
**6**	TMG	23.3	2:2:1	40	0
**7**	proton sponge	7.5[b]	3:3:1	23	0
**8**	BTPP	26.9	3:3:1	0	0

[a]r.s.m.: remaining starting material.[b]pKa value obtained in DMSO

E. UNSUCCESFULL SUBSTRATES

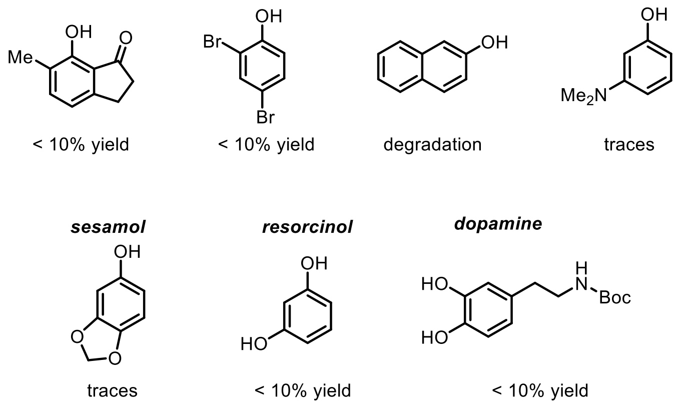

Figure S14. Unsuccessful substrates in the photochemical alkylation process.

F. PRODUCT MANIPULATIONS

F.1. DESULFONYLATION OF THE PRODUCTS **7a**, **7d** AND **7o**

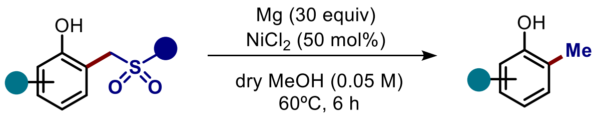

According to a modified literature procedure:[13] To a round-bottom flask containing freshly activated Mg (30 equiv) under N_2, it was added dry MeOH (0.05 M) followed by the sulfonylated phenol **7** (1 equiv) and anhydrous $NiCl_2$ (0.05 equiv, 50 mol%). The mixture was stirred vigorously at 60 ºC for 6 h, in an oil bath. The reaction was quenched by adding an aqueous solution of HCl (1 M). The crude mixture was then transferred to a separatory funnel and extracted with ethyl acetate (x3 times). The organic phases were combined and dried over Mg_2SO_4 before concentration *in vacuo*. The mixture was purified by flash column chromatography (hexane/ethyl acetate 9:1) to give the product **8** in the state yield.

Characterization Data

o-cresol (8a)

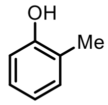

8a

8a was synthesized according to the general procedure **F.1.** from 2-((phenylsulfonyl)methyl)phenol (**7a**) (24.8 mg, 0.2 mmol, 1 equiv.). The final product **8a** was obtained as a white solid (16 mg, 76% yield). The characterization data matched with the reported one.[14]

^{1}H-NMR (300 MHz, $CDCl_3$) δ 7.11 (m, 1H), 6.84 (dd, *J* = 7.4 Hz, 1H), 6.77 (dd, *J* = 7.9 Hz, 1H), 4.65 (s, 1H), 2.26 (s, 3H).

2,6-dimethylphenol (8b)

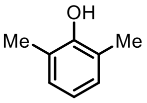

8b

8b was synthesized according to the general procedure **F.1.** from 2-((phenylsulfonyl)methyl)-[1,1'-biphenyl]-3-ol (*o,o'*-**7a**) (80 mg, 0.2 mmol, 1 equiv.). The final product **8b** was obtained as a white solid (15.9 mg, 65% yield). The characterization data matched with the reported one.[15]

^{1}H NMR (300 MHz, $CDCl_3$) δ 6.97 (d, J = 7.5 Hz, 2H), 6.75 (t, J = 7.5 Hz, 1H), 2.25 (s, 6H).

4-methyl-[1,1'-biphenyl]-3-ol (8c)

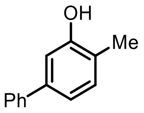

8c

8c was synthesized according to the general procedure **F.1.** from 2-((phenylsulfonyl)methyl)-[1,1'-biphenyl]-3-ol (**7d**) (64 mg, 0.2 mmol, 1 equiv.). The final product **8c** was obtained as a white solid (28 mg, 75% yield). The characterization data matched with the reported one.[16]

^{1}H NMR (300 MHz, $CDCl_3$) δ 7.59 – 7.51 (m, 2H), 7.46 – 7.38 (m, 2H), 7.36 – 7.29 (m, 1H), 7.19 (d, *J* = 7.8 Hz, 1H), 7.13 – 7.06 (m, 1H), 7.02 (d, *J* = 1.8 Hz, 1H), 4.76 (s, 1H), 2.29 (s, 3H).

N-(4-hydroxy-3-methylphenyl)acetamide (8d)

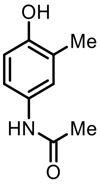

8d

8d was synthesized according to the general procedure **E.1.** from N-(4-hydroxy-3-((phenylsulfonyl)methyl)phenyl)acetamide **7n** (30.5 mg, 0.1 mmol, 1 equiv.). The final product **8d** was obtained as a white solid (13.4 mg, 81% yield). The characterization data matched with the reported one.[21]

^{1}H NMR (400 MHz, MeOD-d_4) δ 7.17 (d, *J* = 2.6 Hz, 1H), 7.16 – 7.09 (m, 1H), 6.67 (d, *J* = 8.4 Hz, 1H), 2.16 (s, 3H), 2.07 (s, 3H).

G. MECHANISTIC INSIGHTS

G.1. ABSORPTION SPECTRA

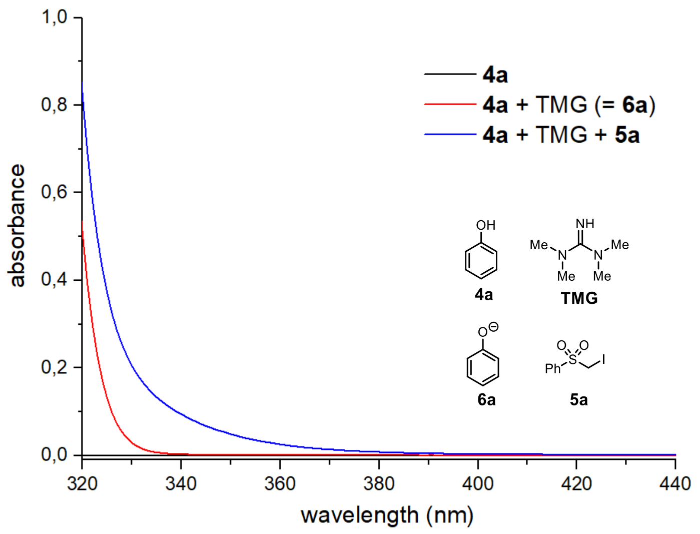

Figure S15. Optical absorption spectra of phenol **4a** (black line), phenolate (**6a**, red line) and the mixture between α-iodosulfone **5a** and phenolate **6a** (blue line). Recorded in CH_3CN in quartz cuvettes (1 mm path). [**4a**] = [TMG] = 0.015 M, [**5a**] = 0.005 M.

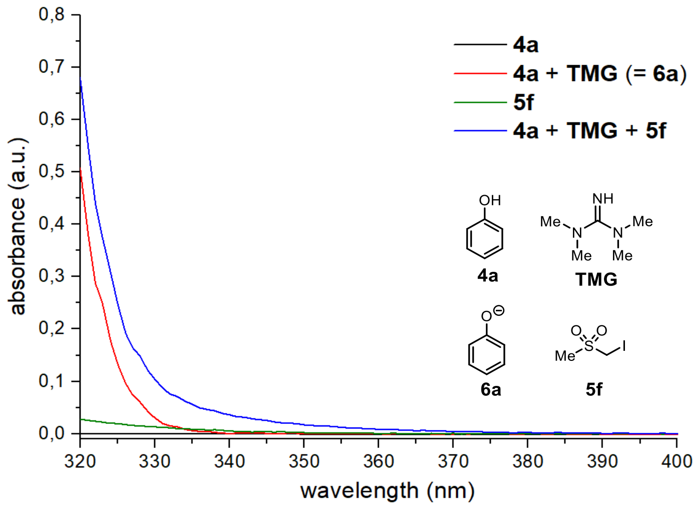

Figure S16. Optical absorption spectra of phenol **4a** (black line), phenolate (**6a**, red line), iodo(methylsulfonyl)methane (**5f**, green line) and the mixture between the α-iodosulfone **5f** and the phenolate **6a** (blue line). Recorded in CH_3CN in quartz cuvettes (1 mm path); [**4a**] = [TMG] = 0.015 M, [**5f**] = 0.005 M.

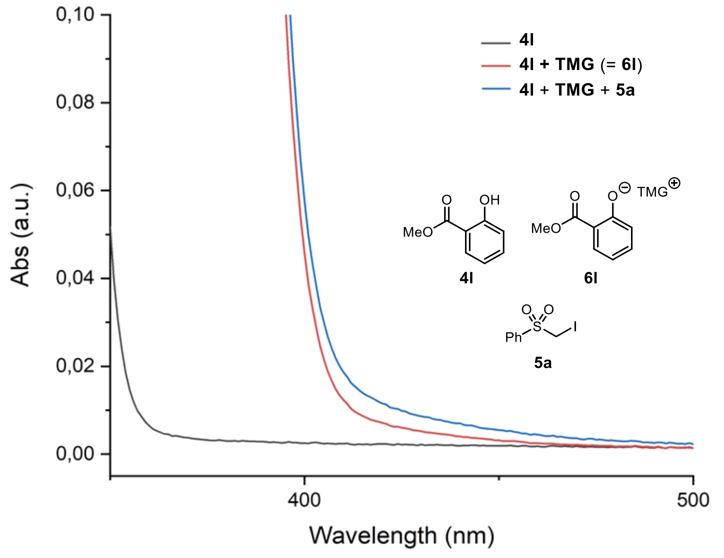

Figure S17. Optical absorption spectra of phenol **4l** (grey line), phenolate (**6l**, red line) and the mixture between α-iodosulfone **5a** and phenolate **6l** (blue line). Recorded in CH_3CN in quartz cuvettes (1 mm path); [**4l**] = [TMG] = 0.015 M, [**5a**] = 0.005 M.

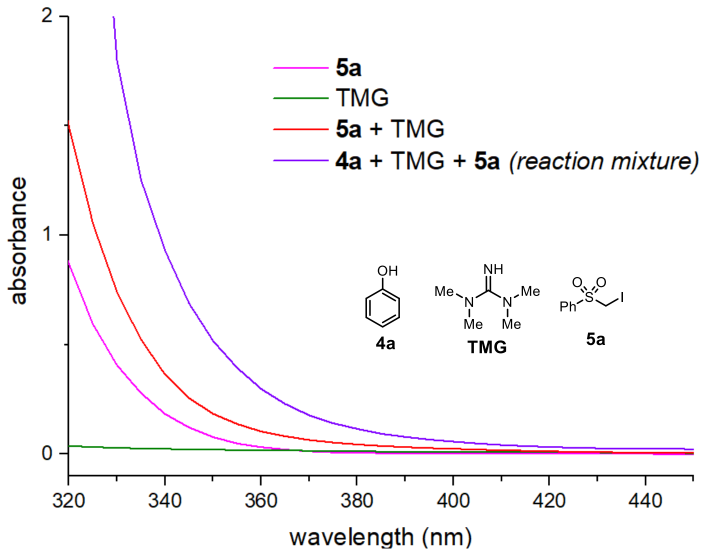

Figure S18. Optical absorption spectra of α-iodosulfone **5a** (pink line), TMG (gren line), the mixture between α-iodosulfone **5a** and TMG (red line), and the reaction mixture of the alkylation process (purple line). Recorded in CH_3CN in quartz cuvettes (1 mm path); [**5a**] = 0.5 M; [**4a**] = [TMG] = 1.5 M.

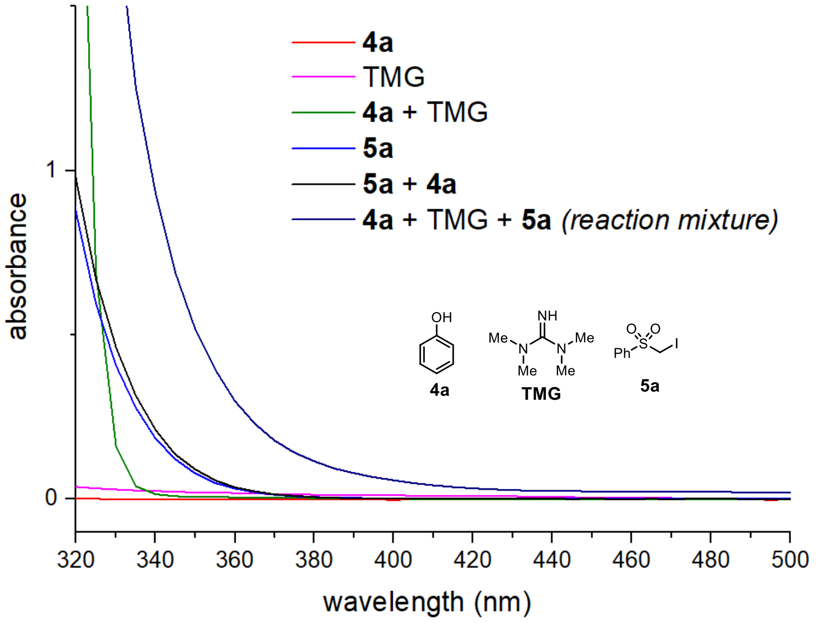

Figure S19. Optical absorption spectra of phenol **4a** (red line), TMG (pink line), phenolate **6a** (green line), α-iodosulfone **5a** (blue line), the mixture between phenol **4a** and iodosulfone **5a** (grey line) and the reaction mixture of the photoalkylation process (dark blue line). Recorded in CH_3CN in quartz cuvettes (1 mm path); [**5a**] = 0.5 M; [**4a**] = [TMG] = 1.5 M.

G.2.CYCLIC VOLTAMMETRY MEASUREMENTS

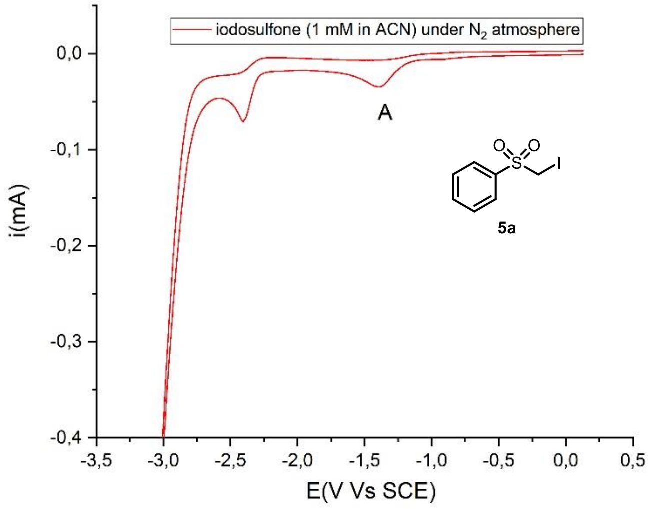

Figure S20. Cyclic voltammogram for α-iodosulfone **5a** (1mM) in $TBAPF_6$ in CH_3CN (0.1 M). Scan rate: 0.1 V/s. Glassy carbon working electrode; Ag/AgCl (3M, NaCl) reference electrode; Pt auxiliary electrode. Irreversible reduction, E_p^A = E^{red}(**5a**/**5a**$^{•-}$) = -1.38 vs SCE. E_p^C refers to the cathodic peak potential, while the E^{red} value describes the electrochemical properties of **5a.**

G.3. DFT STUDIES

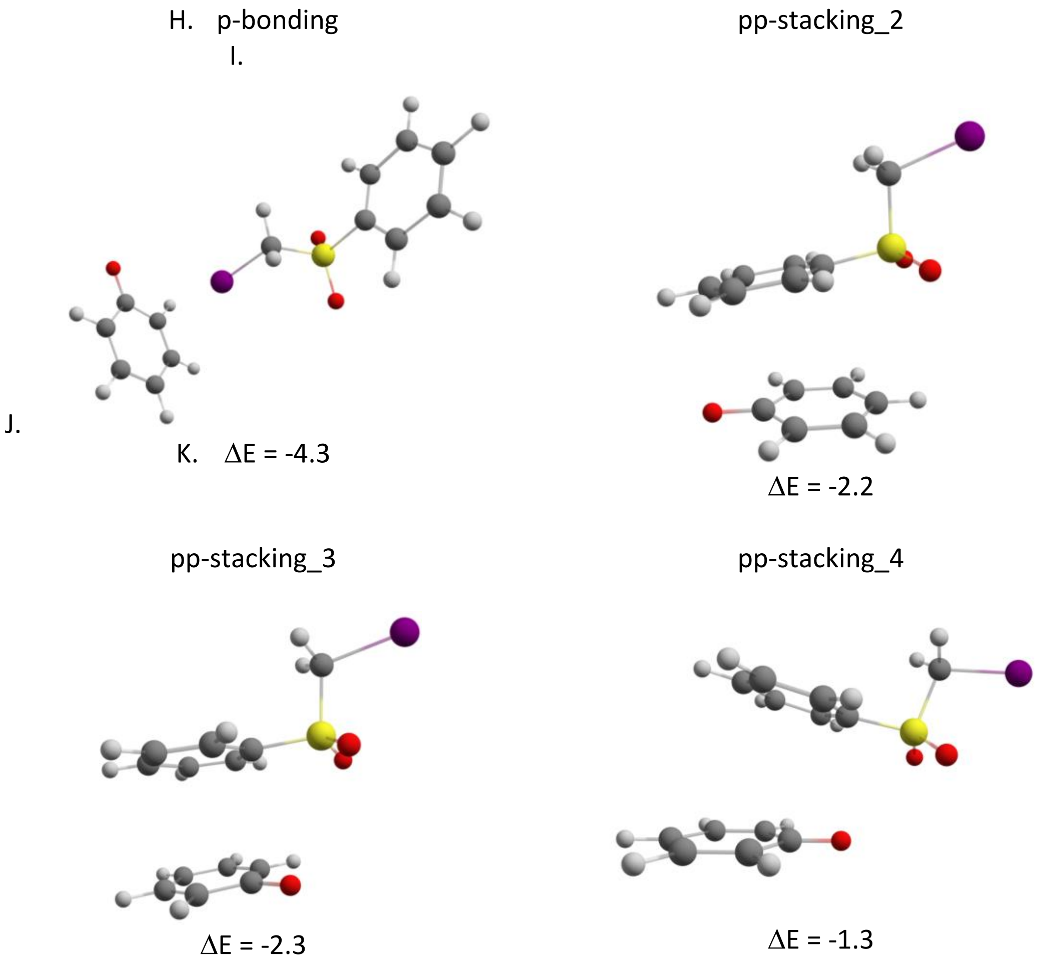

Figure S21. Optimized structures of the bimolecular complexes between phenolate **6a** and α-iodosulfone **5a**. Calculations performed at the M06-2x/Def2TZVP including continuum solvation model (IEFPCM, solvent = MeCN). ΔE represents the binding energies corrected with zero point vibrational energy reported in kcal mol^{-1}.

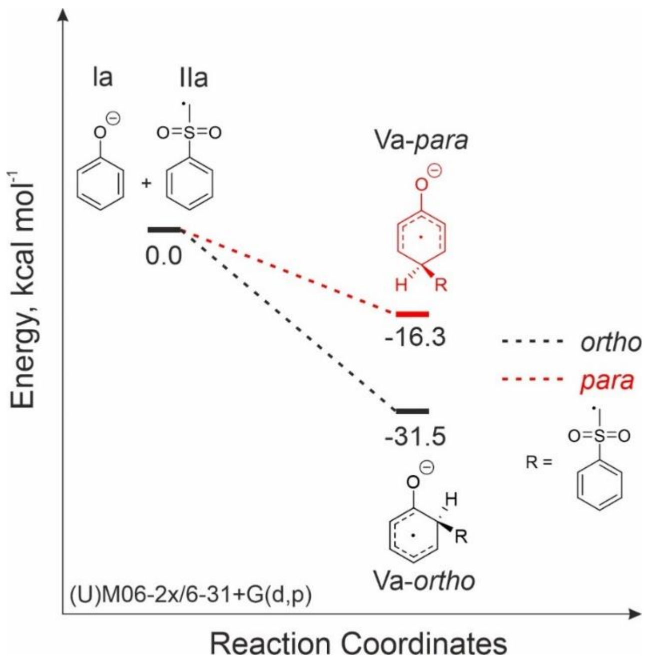

Figure S22. Computed pathways for the sulphonyl radical addition into the phenolate in *ortho* and *para* positions. Calculations performed at the M06-2x/6-31+G(d,p). The energies are corrected with zero point vibrational energy reported in kcal mol^{-1}.

Phenolate; 12 atoms; singlet

Energy= -192572.9367

C	0.00000000	1.19458700	-1.09595200
C	0.00000000	0.00000000	-1.81585200
C	0.00000000	-1.19458700	-1.09595200
C	0.00000000	-1.20375300	0.28833100
C	0.00000000	0.00000000	1.06167500
C	0.00000000	1.20375300	0.28833100
H	0.00000000	2.13995200	-1.62936800
H	0.00000000	0.00000000	-2.89776500
H	0.00000000	-2.13995200	-1.62936800
H	0.00000000	-2.14585700	0.82686400
H	0.00000000	2.14585700	0.82686400
O	0.00000000	0.00000000	2.33991200

α-iodosulfone; 18 atoms; singlet

Energy= -700964.8339

C	4.59400400	-0.00002100	-0.75834000
C	3.94872100	1.20725700	-0.51812000
C	2.64834500	1.21484500	-0.03682600
C	2.01717900	0.00000500	0.19837400
C	2.64833900	-1.21484800	-0.03677600
C	3.94871400	-1.20728700	-0.51807100
H	5.60928700	-0.00003200	-1.13254600
H	4.45878900	2.14303500	-0.70174800
H	2.13174000	2.14339000	0.16621500
H	2.13172900	-2.14338200	0.16630300
H	4.45877800	-2.14307500	-0.70166200
S	0.35738900	0.00002200	0.79342400
O	0.10619100	-1.24143100	1.47784900
O	0.10619100	1.24151200	1.47778100
C	-0.57630500	-0.00002000	-0.72617200
I	-2.65896600	-0.00000800	-0.32444100
H	-0.32819500	0.89907100	-1.28274400
H	-0.32819600	-0.89914200	-1.28269500

X_Bonded (complex ii from ms); 30 atoms; singlet

Energy= -893544.997

C	4.78587900	1.93463900	0.71177800
C	5.87415300	1.91820600	-0.15799400
C	6.10244300	0.76468700	-0.90550400

C	5.27081300	-0.33775400	-0.79569800
C	4.15726600	-0.35321400	0.08739100
C	3.94806600	0.83794200	0.83461400
H	4.58778000	2.82052700	1.30574700
H	6.52612300	2.77692200	-0.24834200
H	6.94317600	0.72696500	-1.59005500
H	5.46045500	-1.22792600	-1.38543300
H	3.10712900	0.86610400	1.51885400
O	3.38433600	-1.38575500	0.19902100
C	-6.28594500	0.66334800	-0.85501500
C	-5.91843300	-0.52285200	-0.23083000
C	-4.62974800	-0.67976600	0.25754100
C	-3.72569700	0.36436900	0.11185200
C	-4.07900000	1.55491000	-0.50952400
C	-5.37095400	1.70005300	-0.99337300
H	-7.29305400	0.78185100	-1.23268600
H	-6.63616700	-1.32450400	-0.12052600
H	-4.32798900	-1.59171500	0.75544600
H	-3.35525200	2.35397700	-0.59925200
H	-5.66369400	2.62328900	-1.47494400
S	-2.07358700	0.16235700	0.71183000
O	-1.52602200	1.47291600	0.96913000
O	-2.10173300	-0.79747100	1.78942900
C	-1.22388300	-0.55006000	-0.66384700
I	0.85580300	-0.90839200	-0.20625200
H	-1.71315800	-1.49113100	-0.90195800
H	-1.29792200	0.14813000	-1.49375700

ppstacking1 (complex i from ms); 30 atoms; singlet
Energy= -893545.5781

C	-3.26077100	-1.64743700	1.54076400
C	-4.38709100	-1.69793400	0.72140300
C	-4.19799100	-1.93089500	-0.64022200
C	-2.93061500	-2.08947700	-1.17313200
C	-1.75706900	-2.02607500	-0.36667600
C	-1.98646400	-1.81327400	1.02405000
H	-3.38101200	-1.47661800	2.60567100
H	-5.38004100	-1.56949100	1.13160300
H	-5.05879900	-1.98066700	-1.29930400
H	-2.79993100	-2.25569200	-2.23740700
H	-1.12347300	-1.77559700	1.68110800

O	-0.57469900	-2.12916200	-0.86320700
C	-3.30988400	2.40280200	0.30441000
C	-2.31321600	3.11161200	0.96569700
C	-0.97863000	2.82299700	0.72097200
C	-0.67011200	1.82973100	-0.19928600
C	-1.65445500	1.11179600	-0.86715200
C	-2.98406100	1.40475000	-0.60625400
H	-4.34967300	2.62720100	0.50485900
H	-2.57428400	3.88729400	1.67296800
H	-0.18581200	3.35978500	1.22390000
H	-1.38060600	0.32567600	-1.55912900
H	-3.76034200	0.83715800	-1.10070500
S	1.01955600	1.42530200	-0.50196100
O	1.18232500	1.09389700	-1.89611300
O	1.85251400	2.46148400	0.05871700
C	1.19753500	-0.06123300	0.45151200
I	3.16742700	-0.83127800	0.26820100
H	1.01504700	0.17896900	1.49525100
H	0.50821400	-0.81905300	0.04618100

Ppstacking2_complex; 30 atoms; singlet

Energy= -893539.9217

C	-3.00622500	-1.95805700	-1.19516300
C	-2.53468800	-2.49972100	0.00063100
C	-3.00558000	-1.95678400	1.19609900
C	-3.90489900	-0.90620500	1.20436400
C	-4.40551700	-0.31961300	-0.00001900
C	-3.90555100	-0.90748900	-1.20405400
H	-2.65323500	-2.36079800	-2.13965900
H	-1.82494300	-3.31681400	0.00087800
H	-2.65206300	-2.35850400	2.14083400
H	-4.24938400	-0.48830000	2.14501700
H	-4.25057400	-0.49056700	-2.14494600
O	-5.23501600	0.65274100	-0.00031400
C	-2.72561900	2.57548000	-0.00067400
C	-2.20592100	2.12179100	-1.20636900
C	-1.15679500	1.21700600	-1.21397200
C	-0.64153200	0.77953800	0.00001800
C	-1.15617000	1.21868500	1.21368400
C	-2.20530500	2.12344300	1.20539000
H	-3.54943600	3.27579300	-0.00096100

H	-2.62689400	2.46235500	-2.14286100
H	-0.74578300	0.84419400	-2.14285600
H	-0.74465600	0.84716900	2.14286600
H	-2.62576200	2.46532300	2.14163300
S	0.76193300	-0.27944700	0.00039300
O	0.82572000	-1.00603400	1.24317600
O	0.82537100	-1.00744700	-1.24157500
C	2.09240000	0.91317000	-0.00052600
I	3.98009500	-0.05595800	-0.00021100
H	2.01313000	1.51718500	-0.89943500
H	2.01343400	1.51832300	0.89764100

Ppstacking3_complex; 30 atoms; singlet

Energy= -893540.0309

C	-5.06058100	0.04016300	0.21830000
C	-5.12782700	-0.50855700	-1.06237700
C	-4.20031200	-1.49373100	-1.39978900
C	-3.23495000	-1.91208300	-0.50096900
C	-3.12549200	-1.36184700	0.81556700
C	-4.10303300	-0.36742300	1.12885600
H	-5.76988900	0.80994400	0.50706100
H	-5.87837300	-0.18246500	-1.77069100
H	-4.23032400	-1.94034200	-2.38894300
H	-2.51555600	-2.67274800	-0.78808200
H	-4.06372400	0.08054900	2.11657400
O	-2.22609300	-1.73109400	1.64584300
C	-2.68235900	2.50073900	-0.41554900
C	-2.35151600	1.52490500	-1.34482100
C	-1.28655800	0.67006500	-1.10262400
C	-0.56912700	0.81523500	0.07689100
C	-0.89359500	1.78094300	1.02120200
C	-1.96193600	2.62655400	0.76711900
H	-3.51960300	3.16000700	-0.60546800
H	-2.93342700	1.41346500	-2.24903600
H	-1.01890800	-0.10387200	-1.80879200
H	-0.32280500	1.85790900	1.93720300
H	-2.23424100	3.38092700	1.49309700
S	0.84211500	-0.20006600	0.34967300
O	1.10932300	-0.29440000	1.76183100
O	0.73772800	-1.40498400	-0.43254600
C	2.13093300	0.80361200	-0.37616700

I	4.01432600	-0.16163900	-0.21744000
H	1.90494200	0.95014500	-1.42814900
H	2.17922800	1.74678100	0.15992400

Ppstacking4_complex; 30 atoms; singlet

Energy= -893539.0953

C	-5.05826900	-0.51899400	1.19758800
C	-5.70312200	-0.20500200	0.00038100
C	-5.06033300	-0.53272300	-1.19425300
C	-3.81793600	-1.14206900	-1.20070300
C	-3.12180900	-1.47197700	0.00549200
C	-3.81586800	-1.12822300	1.20898900
H	-5.53769500	-0.27808000	2.14136000
H	-6.67583700	0.26910900	-0.00145000
H	-5.54135800	-0.30260500	-2.13989300
H	-3.33076100	-1.38212900	-2.14047400
H	-3.32731000	-1.35754500	2.15073100
O	-1.97520700	-2.03221900	0.00767000
C	-2.53225800	2.60810300	-0.00935200
C	-2.03536500	2.12229700	-1.21299600
C	-1.01471200	1.18349600	-1.21649700
C	-0.51083400	0.74042100	-0.00165400
C	-1.00511800	1.20415400	1.20959100
C	-2.02600700	2.14256100	1.19843400
H	-3.32646300	3.34322000	-0.01246000
H	-2.44406400	2.47313300	-2.15121900
H	-0.61750500	0.79190700	-2.14357100
H	-0.60055200	0.82847500	2.14008400
H	-2.42719800	2.50926700	2.13382200
S	0.87723000	-0.34616400	0.00259200
O	0.94927800	-1.05804100	1.25166600
O	0.94895700	-1.06870700	-1.24032000
C	2.20742200	0.85163900	-0.00308400
I	4.09648100	-0.11545700	-0.00096800
H	2.12938200	1.45278100	-0.90397200
H	2.13121500	1.45995600	0.89311800

pbonding_complex; 30 atoms; singlet

Energy= -893542.0502

C	4.64550900	1.32263400	1.23615200
C	4.42295500	1.87999200	-0.02516500

C	4.20045900	1.01251400	-1.09058100
C	4.19264100	-0.36382000	-0.91337900
C	4.42062700	-0.97207200	0.36286700
C	4.64494000	-0.04569600	1.43173900
H	4.82056900	1.97633600	2.08467600
H	4.42292700	2.95213300	-0.16893200
H	4.02036900	1.41622500	-2.08181800
H	4.01905400	-1.02164200	-1.75888900
H	4.81732900	-0.45530500	2.42153600
O	4.41390100	-2.23591000	0.53519700
C	-6.14302600	-0.12215700	1.05585400
C	-5.46058800	1.08686300	1.11794500
C	-4.20151300	1.20290500	0.54815900
C	-3.64788900	0.09523900	-0.08060400
C	-4.31790800	-1.11951500	-0.15126300
C	-5.57594200	-1.22198400	0.42290800
H	-7.12564800	-0.20702000	1.50095200
H	-5.90971200	1.94074000	1.60675900
H	-3.65781200	2.13770700	0.57737900
H	-3.86319900	-1.96121600	-0.65659200
H	-6.11445800	-2.15860200	0.37285900
S	-2.03697400	0.22751900	-0.79102200
O	-1.91713000	-0.75050100	-1.84273500
O	-1.78008200	1.61271500	-1.09444800
C	-0.98419100	-0.26012900	0.55550900
I	1.06868500	-0.21511400	-0.02004900
H	-1.13877100	0.43653600	1.37471700
H	-1.24932800	-1.27421500	0.84135300

Sulphonyl radical IIa; 17 atoms; doublet

Energy= -514242.7633

C	0.07332700	3.02977500	0.00000000
C	0.07446000	2.34054000	1.20713900
C	0.07446000	0.95416300	1.21440100
C	0.07807700	0.27754300	0.00000000
C	0.07446000	0.95416300	-1.21440100
C	0.07446000	2.34054000	-1.20713900
H	0.07489400	4.11187200	0.00000000
H	0.07926600	2.88279500	2.14295100
H	0.08468500	0.39940800	2.14300300
H	0.08468500	0.39940800	-2.14300300
H	0.07926600	2.88279500	-2.14295100

S	0.05776300	-1.48668800	0.00000000
O	0.62447800	-1.94353900	-1.24444700
O	0.62447800	-1.94353900	1.24444700
C	-1.62396200	-1.88256500	0.00000000
H	-2.13516900	-1.93879700	0.94848900
H	-2.13516900	-1.93879700	-0.94848900

Intermediate Va-*ortho*; 29 atoms; doublet

Energy= -706897.2940

C	2.64353100	-1.23828000	0.70500800
C	3.70557600	-1.21909600	-0.14852000
C	4.17344100	-0.01573100	-0.72425700
C	3.52125200	1.19760900	-0.45525200
C	2.40439300	1.29126800	0.38721300
C	1.95150800	0.02301300	1.13291700
H	2.29500400	-2.17097800	1.13383600
H	4.20008900	-2.15132000	-0.40026100
H	2.18301200	0.22938100	2.19413700
O	1.76542400	2.35408800	0.63019700
S	-0.32340800	-0.64371400	-0.35297200
O	-0.28353500	-2.08897000	-0.40633500
O	0.21861200	0.09589900	-1.47002100
C	0.41722100	-0.07507800	1.16537700
H	0.00454000	0.91686200	1.35010600
H	0.06112300	-0.77289100	1.92435400
H	3.85622500	2.11579900	-0.92669000
H	5.02249800	-0.03721500	-1.39589000
C	-2.02167600	-0.16531300	-0.16793100
C	-2.38246900	1.14700000	-0.44800800
C	-2.95279900	-1.10047100	0.25832600
C	-3.70753600	1.52560900	-0.29817400
H	-1.63450800	1.85412300	-0.78394200
C	-4.27819200	-0.71145800	0.40234500
H	-2.64153800	-2.11627900	0.46136800
C	-4.65223900	0.59696700	0.12584400
H	-4.00453200	2.54274500	-0.51587100
H	-5.01729800	-1.43117400	0.72784700
H	-5.68616600	0.89599000	0.23850300

Intermediate Va-*para*; 29 atoms; doublet

Energy= -706888.7579

C	2.29078300	1.19143700	1.12315900

C	1.68659800	-0.14196000	1.46771500
C	2.40283500	-1.26525700	0.77198500
C	3.43578300	-1.06115500	-0.08342600
C	3.97956300	0.23444800	-0.40232100
C	3.33717200	1.33241000	0.27126400
H	1.85603600	2.06360400	1.60121200
O	4.96788000	0.39269700	-1.21141200
S	-0.44703600	-0.29360800	-0.40050700
O	-0.27047500	-1.64547700	-0.88131500
O	0.08238000	0.79133700	-1.19343000
C	0.14387100	-0.17616800	1.27807600
H	3.90600600	-1.91782900	-0.55905200
H	2.05270400	-2.27233500	0.97112800
C	-2.19040000	-0.02260800	-0.21307400
C	-2.68249700	1.27534900	-0.25504700
C	-3.02759400	-1.11168300	-0.01388400
C	-4.04449600	1.48294900	-0.09446000
H	-2.00717700	2.10431600	-0.42196600
C	-4.38872000	-0.89284200	0.14553300
H	-2.61631800	-2.11223200	0.00338100
C	-4.89374700	0.40095300	0.10654700
H	-4.44333500	2.48785000	-0.12974100
H	-5.05425100	-1.73210400	0.29667500
H	-5.95587100	0.56737000	0.22992600
H	-0.29715600	-1.04142200	1.77759800
H	-0.30615300	0.73213800	1.68429500
H	1.75495800	-0.29115800	2.56019900
H	3.72994400	2.32583000	0.07129500

G.4. QUANTUM YIELD MEASUREMENT

A ferrioxalate actinometry solution was prepared by following the Hammond variation of the Hatchard and Parker procedure outlined in Handbook of Photochemistry.[17] Ferrioxalate actinometer solution measures the decomposition of ferric ions to ferrous ions, which are complexed by 1,10-phenanthroline (complete complexation takes about an hour) and monitored by UV/Vis absorbance at 510 nm.[18] The moles of iron-phenanthroline complex formed are related to moles of photons absorbed.

The following solutions were prepared and stored in the dark:

1. *Potassium ferrioxalate solution*: 589.5 mg of potassium ferrioxalate (commercially available from Alfa Aesar) and 278 μL of sulfuric acid (96%) were added to a 100 mL volumetric flask and filled to the mark with water (MilliQ grade).

2. *Phenantroline solution:* 0.2% by weight of 1,10-phenanthroline in water (200 mg in 100 mL volumetric flask).

3. *Buffer solution*: to a 100 mL volumetric flask 4.94 g of NaOAc and 1 mL of sulfuric acid (96%) were added and filled to the mark with water (MilliQ grade).

4. *Model reaction solution I*: the mixture between phenol **4a** and α-iodosulfone **5a** was added to a 1 mL volumetric flask, using the described reaction conditions (see Section C) and dibromomethane as internal standard, and filled to the mark with acetonitrile (HPLC grade).

5. *Model reaction solution II*: a mixture between phenol **4l** and α-iodosulfone **5a** was added to a 1 mL volumetric flask, using the described reaction conditions (see Section C) and dibromomethane as internal standard, and filled to the mark with acetonitrile (HPLC grade).

The actinometry measurements were done as follows:

A1. 365 nm LED: 1 mL of the actinometer solution was added to a quartz cuvette (l = 10 mm). The actinometry solution (placed 2 cm away from the lamp) were irradiated with 3 W 365 nm LED for specified time intervals (0, 10, 20, 30) seconds.

A2. 456 nm LED: 1 mL of the actinometer solution was added to a quartz cuvette (l = 10 mm). The actinometry solution (placed 1 cm away from the lamp) were irradiated with 3 W 456 nm LED for specified time intervals (0, 15, 30, 45, 60) seconds.

B. After irradiation all the actinometer solution was removed and placed in a 10 mL volumetric flask. 0.5mL of 1,10-phenanthroline solution and 2 mL of buffer solution was added to this flask and filled to the mark with water (MilliQ grade).

C. The UV-Vis spectra of actinometry samples were recorded for each time interval (Figure S23 and Figure S24). The absorbance of the actinometry solution was monitored at 510 nm.

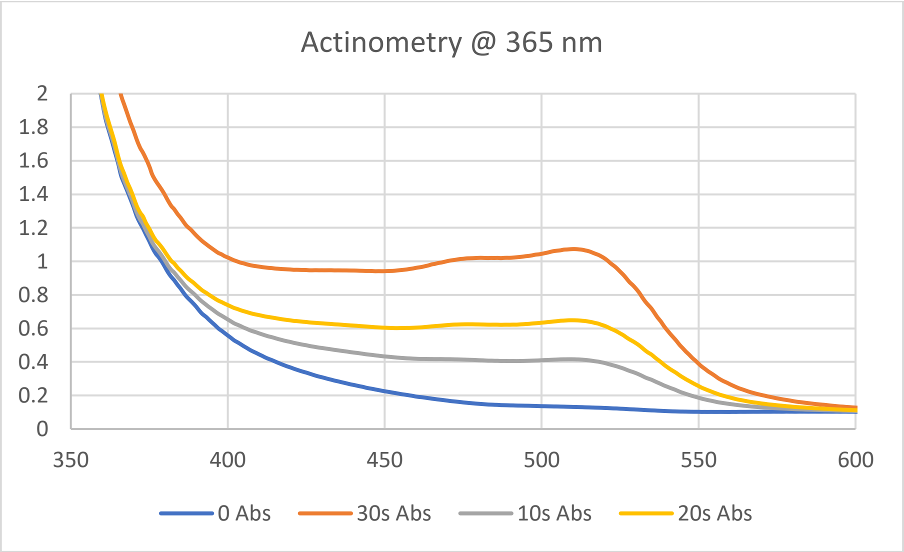

Figure S23. UV-Vis spectra of actinometry samples irradiated with 365 nm light for the indicated time intervals.

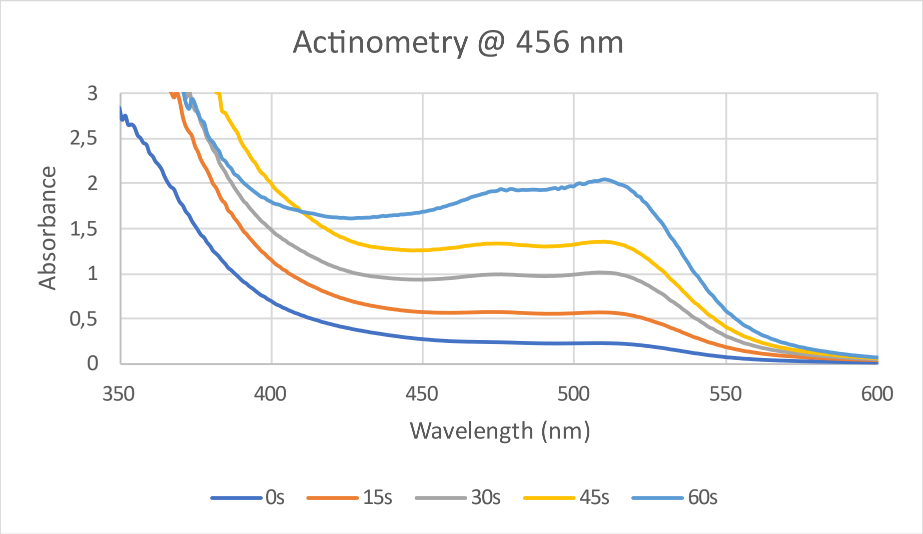

Figure S24. UV-Vis spectra of actinometry samples irradiated with 456 nm light for the indicated time intervals.

D. The moles of Fe^{2+} formed for each sample is determined using Beer's Law:

$$mmoles\ Fe^{2+} = \frac{V_1 V_3 \Delta A_{(510\ nm)}}{10^3 V_2 l \varepsilon_{(510\ nm)}}$$

Equation S1. Lambert-Beer's law.

where V_1 is the irradiated volume (1 mL), V_2 is the aliquot of the irradiated solution taken for the determination of the ferrous ions (1 mL), V_3 is the final volume after complexation with phenanthroline (10 mL), l is the optical path-length of the irradiation cell (1 cm), $\Delta A_{(510\ nm)}$ the optical difference in absorbance between the irradiated solution and that taken in the dark, $\varepsilon_{(510\ nm)}$ is the molar extinction coefficient of the complex $Fe(phen)_3^{2+}$ (11100 L mol^{-1} cm^{-1}).

E. The moles of Fe^{2+} formed (N) are plotted as a function of time (t) (Figure S25 and Figure S26).

The slope is a product of the photon flux (F) and the quantum yield for Fe^{2+} ($\varphi Fe^{2+} = 1.21$ for λ = 365 nm; and $\varphi Fe^{2+} = 0.9$ for λ = 456 nm),[17] since F = N/ΦFe^{2+} t. The F was determined to be:

365 nm: **2.21 10^{-8} einstein s^{-1}**.

456 nm: **2.77·10^{-8} einstein s^{-1}**.

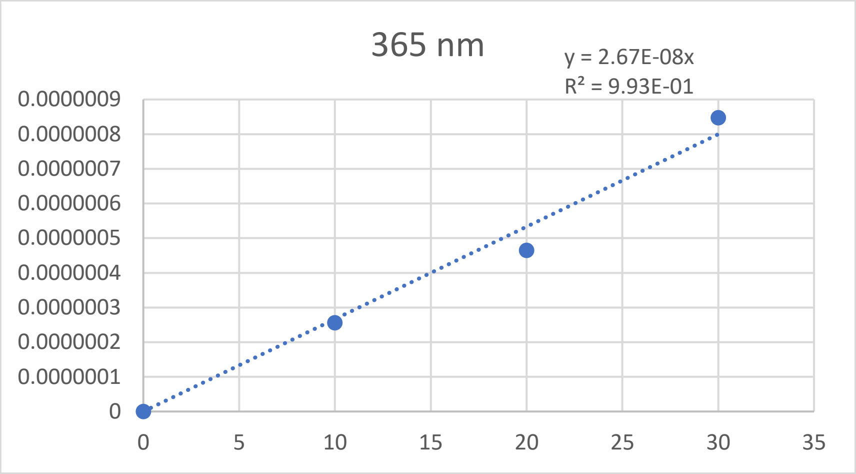

Figure S25. Moles of Fe^{2+} formed after irradiation with 365 nm light as a function of time.

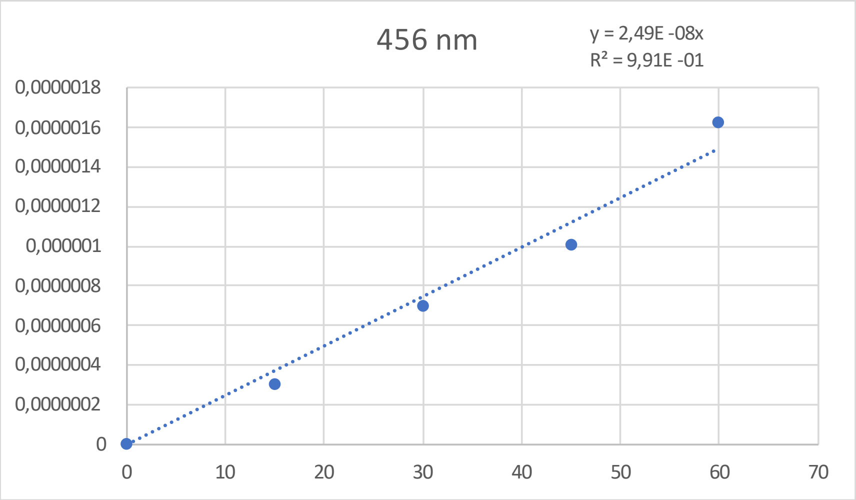

Figure S26. Moles of Fe^{2+} formed after irradiation with 456 nm light as a function of time.

Since the ferrioxalate actinometer absorbance at 365 nm is 1.50 and at 456 nm is 0.26, and inferior to 2 in both cases (if it is major than 2 at the wavelength used, it can be assumed that the entire incident light is absorbed), a correction factor (C) based on the fraction of light absorbed by the actinometer has to be considered to calculate the photon flux and the final quantum yield of the reaction[17] (note that is also needed if the absorbance of the reaction under study at the optimized concentration is inferior to 2 at the wavelength used). Thus, according to the definition of quantum yield:[19]

$$\Phi_{Fe^{2+}} = \frac{\left(\frac{\delta_{molesFe^{2+}}}{\delta_{time}}\right)}{F C_{Fe^{3+}}} \rightarrow F = \frac{\left(\frac{\delta_{molesFe^{2+}}}{\delta_{time}}\right)}{\Phi_{Fe^{2+}}\ C_{Fe^{3+}}} \qquad C_{Fe^{3+}} = [1 - 10^{-A(\lambda)}]$$

Equation S2. Correlation between quantum yield equation and the correction factor.

the photon flux (F) previously found has to be divided by the appropriate correction factor C.

365 nm: C= 0.968, calculated with A_{365nm} = 1.50; corrected F = **2.28 10^{-8} einstein s^{-1}.**

456 nm: C= 0.45, calculated with A_{456nm} = 0.26; corrected F = **6.15·10^{-8} einstein s^{-1}**.

F. The *reaction solutions I* and *II* described at point **4** and **5**, respectively, were irradiated using the same system, and the moles of product formed for the reaction of interest are described below. The moles of product formed were determined by ^{1}H-NMR analysis using dibromomethane as internal standard. The moles of product formed per unit of time were related to the number of photons absorbed.

Reaction solution I

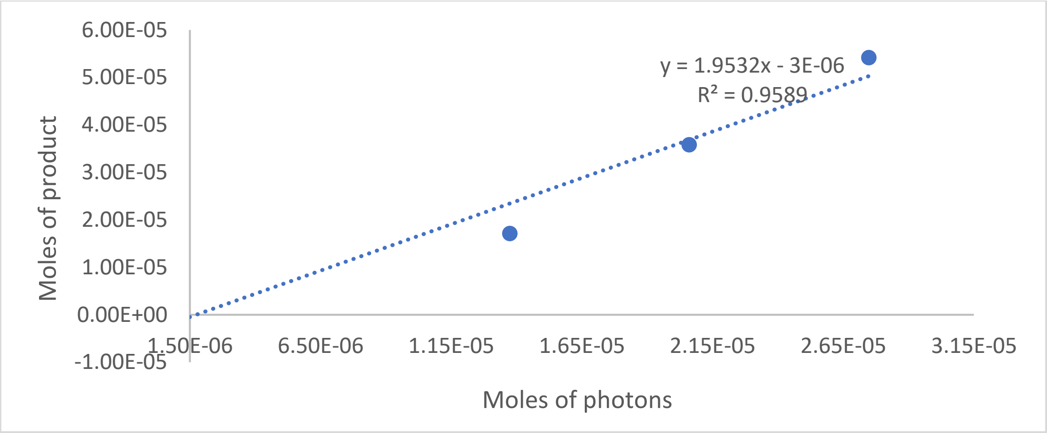

Figure S27. Moles of product formed after irradiation with 365 nm light as a function of the moles of emitted photons.

The absorbance of the reaction at the irradiation wavelength (365 nm) is A_{365nm}> 2, hence no correction factor must be applied since it can be assumed that the entire incident light is absorbed. The **quantum yield (φ)** corresponding to the registered slope is **1.95** (Figure S27).

Reaction solution II

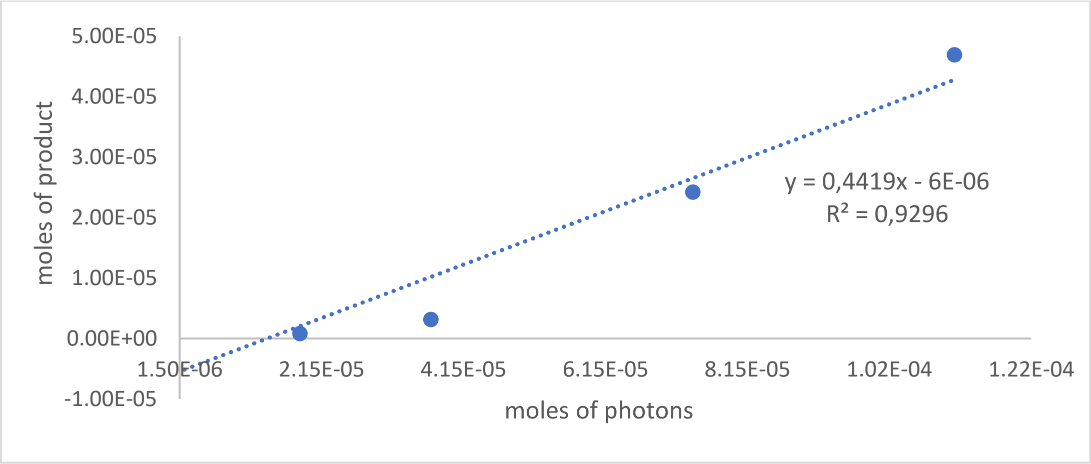

Figure S28. Moles of product formed after irradiation with 456 nm light as a function of the moles of emitted photons.

The absorbance of the reaction at the irradiation wavelength (456 nm) is A_{456nm} = 0.09. The correction factor is C = 0.188 (see equation S2). Therefore, by applying the correction factor (slope/C) the **quantum yield (φ)** is **2.35** (Figure S28).

H. REFERENCES

[1] Nappi, M., Bergonzini, G., Melchiorre, P. Metal-free photochemical aromatic perfluoroalkylation of α-cyano arylacetates. *Angew. Chem. Int. Ed.* **2014**, *53*, 4921–4925.

[2] Filippini, G., Silvi, M., Melchiorre, P. Enantioselective Formal α-Methylation and α-Benzylation of Aldehydes by Means of Photo-organocatalysis. *Angew. Chem. Int. Ed.* **2017**, *56*, 4447–4451.

[3] Zhao, Y., Truhlar, D. G., Zhao, Y., Truhlar, · D G. The M06 suite of density functionals for main group thermochemistry, thermochemical kinetics, noncovalent interactions, excited states, and transition elements: two new functionals and systematic testing of four M06-class functionals and 12 other functionals and inorganometallic chemistry and for noncovalent interactions. *Theor Chem Account*, **2008**, *120*, 215–241.

[4] Tomasi, J., Mennucci, B., Cammi, R. Quantum Mechanical Continuum Solvation Models. *Chem. Rev.* **2005**, *105*, 8, 2999-3094.

[5] Weigend, F., Häser, M., Patzelt, H., Ahlrichs, R. RI-MP2: Optimized Auxiliary Basis Sets and Demonstration of Efficiency. *Chem. Phys. Lett.* **1998**, *294*, 143–152.

[6] https://gaussian.com/g09citation/ - Revision used: E.01

[7] Hart, M. E., Suchland, K. L., Miyakawa, M., Bunzow, J. R., Grandy, D. K., Scanlan, T. S. Trace Amine-Associated Receptor Agonists: Synthesis and Evaluation of Thyronamines and Related Analogues. *J. Med. Chem.* **2006**, *49*, 1101–1112.

[8] Bindman, N. A.; Bobeica, S. C.; Liu, W. R.; van der Donk, W. A. Facile Removal of Leader Peptides from Lanthipeptides by Incorporation of a Hydroxy Acid. *J. Am. Chem. Soc.* **2015**, *137*, 6975−6978.

[9] Betts, H. M., Sephton, S. M., Tong, C., Awais, R. O., Hill, P. J., Perkins, A. C., Aigbirhio, F. I. Synthesis, in Vitro Evaluation, and Radiolabeling of Fluorinated Puromycin Analogues: Potential Candidates for PET Imaging of Protein Synthesis. *J. Med. Chem.* **2016**, *59*, 20, 9422–9430.

[10] Madabhushi, S., Jillella, R., Sriramoju, V., Singh, R. Oxyhalogenation of thiols and disulfides intosulfonyl chlorides/bromides using oxone-KX (X = Cl or Br) in water. *Green Chem.*,**2014**, *16*, 3125–3131.

[11] Imamoto, T., Koto, H. An efficient synthesis of α-iodo derivatives of sulfones, sulfoximines and phosphine oxides. *Synthesis* **1985**, *10*, 982-983.

[12] Zhang, Y.-M., Fan, X., Yang, S.-M., Scannevin, R. H., Burke, S. L., Rhodes, K. J., Jackson, P. F. Syntheses and in vitro evaluation of arylsulfone-based MMP inhibitors with heterocycle-derived zinc-binding groups (ZBGs). *Bioorg. Med. Chem. Lett.* **2008**, *18*, 405–408.

[13] Das, I.; Pathak, T. Desulfonylation with Mg-MeOH-NiBr2: An Expedient Reagent System for the Synthesis of 2-Amino-2,3-Dideoxy Furanosides. *Org. Lett.* **2006**, *8*, 1303–1306.

[14] Xiao, Y., Xu, Y., Cheon, H. S., Chae, J. Copper(II)-Catalyzed Hydroxylation of Aryl Halides Using Glycolic Acid as a Ligand. *J. Org. Chem.* **2013**, 78, 5804–5809.

[15] Qi, H. -L., Chen, D.-S., Ye, J.-S., Huang, J.-M. Electrochemical Technique and Copper-Promoted Transformations: Selective Hydroxylation and Amination of Arylboronic Acids. *J. Org. Chem.* **2013**, *78*, 7482–7487.

[16] Kauch, M., Snieckus, V., Hoppe, D. Substitution of Hydroxybiaryls via Directed Ortho-Lithiation of N-Silylated O-Aryl N-Isopropylcarbamates. *J. Org. Chem.* **2005**, *70*, 7149–7158.

[17] Murov, S. L.; *Handbook of Photochemistry*, Marcel Dekker, New York, **1973**.

[18] Kuhn, H. J., Braslavsky, S. E., Schmidt, R. Chemical Actinometry (IUPAC Technical Report), *Pure Appl. Chem*. **2004**, *76*, 2105-2146.

[19] Braslavsky, S. E.; *Glossary of terms used in photochemistry*, 3rd edition (IUPAC Recommendations 2006) *Pure Appl. Chem.* **2007**, *79*, 293; on page 406.

[20] (a) Aranzaes, J. R., Daniel, M.C., Astruc, D. Metallocenes as references for the determination of redox potentials by cyclic voltammetry – permethylated iron and cobalt sandwich complexes, inhibition by polyamine dendrimers, and the role of hydroxy-containing ferrocenes. *Can. J. Chem.* **2006**, *84*, 288-299; (b) Pavlishchuk, V. V., Addison, A. W. *Inorganica Chim. Acta* **2000**, *298*, 97–102.

[21] H.-Y. Chuang, M. Schupp, R. Meyrelles, B. Maryasin, N. Maulide. Redox-Neutral Selenium-Catalysed Isomerisation of *para*-Hydroxamic Acids into *para*-Aminophenols. *Angew. Chem. Int. Ed.* **2021**, *60*, 13778–13782.

I. NMR SPECTRA

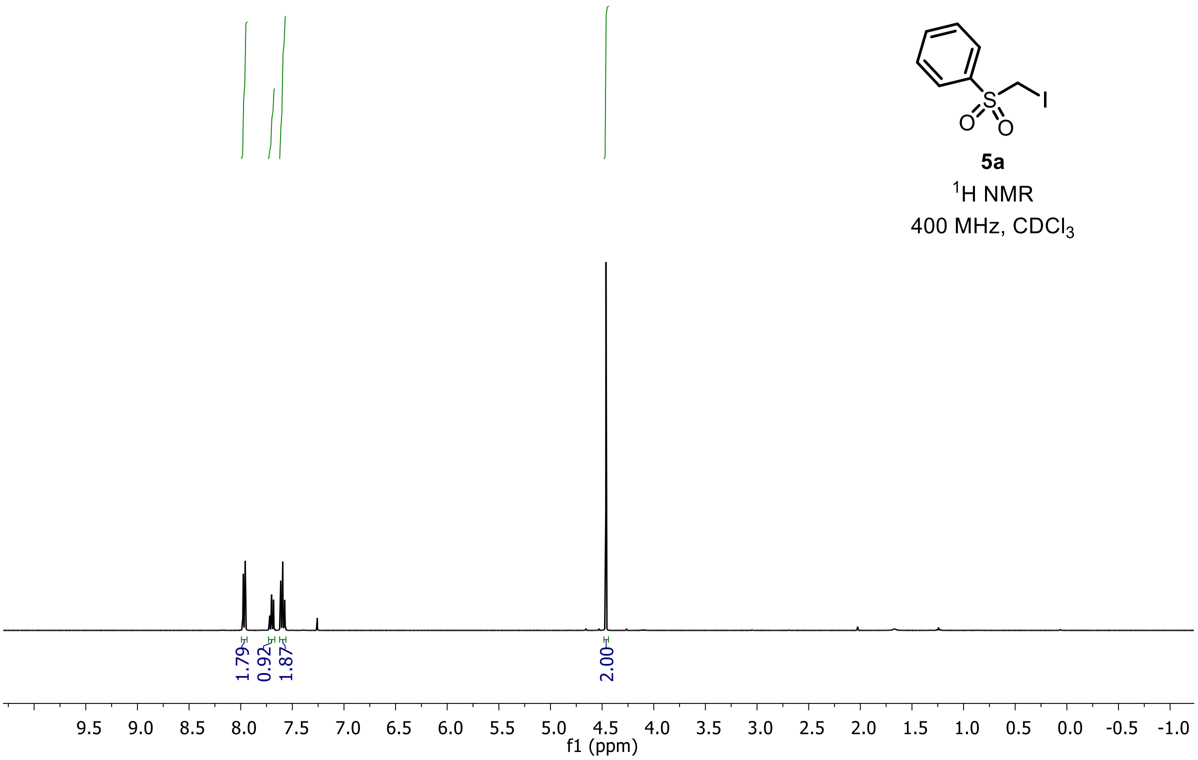

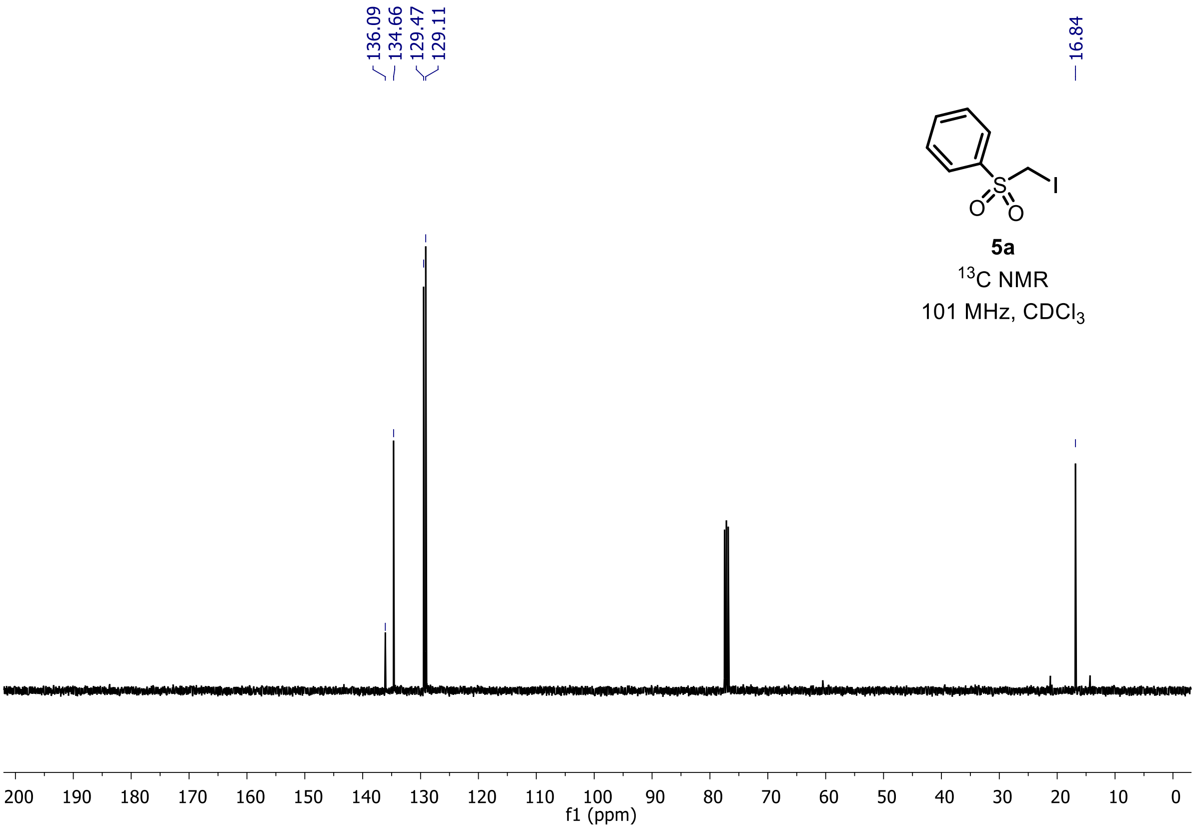

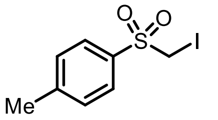

5b

^{1}H NMR

400 MHz, CDCl$_3$**Cl$_3$**

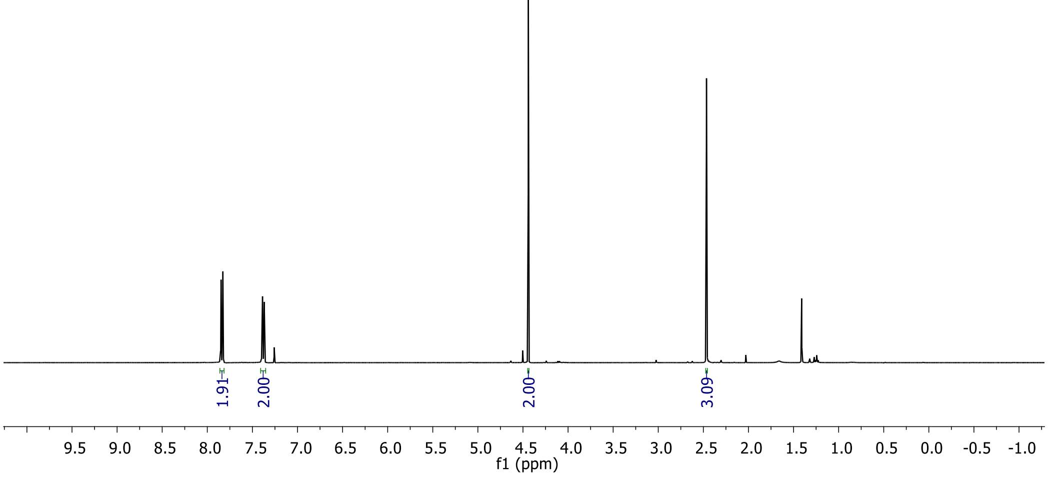

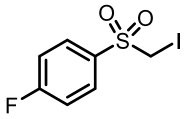

5c

^{1}H NMR

400 MHz, CDCl$_3$

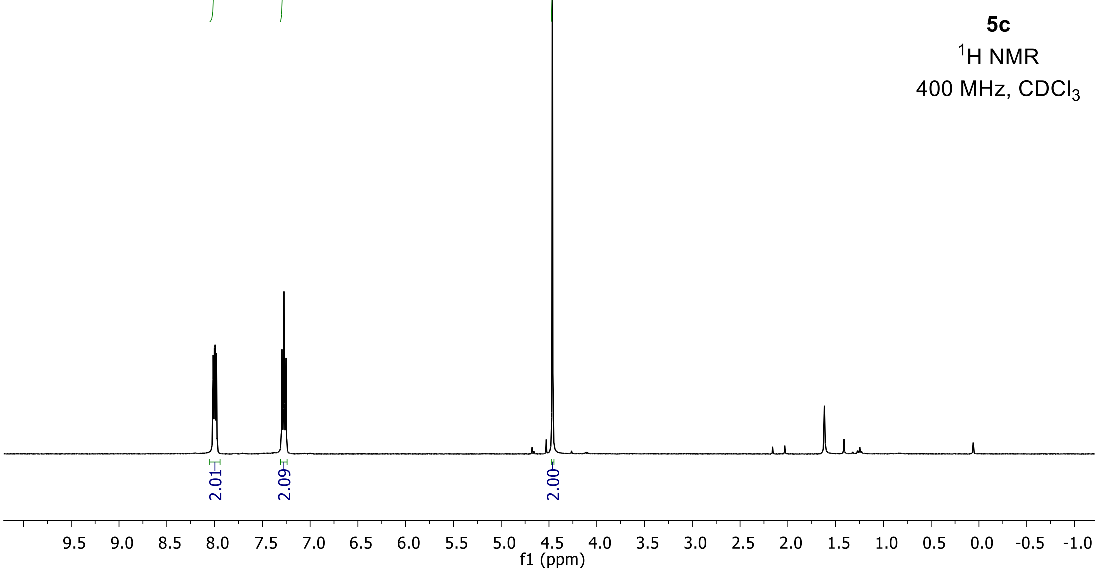

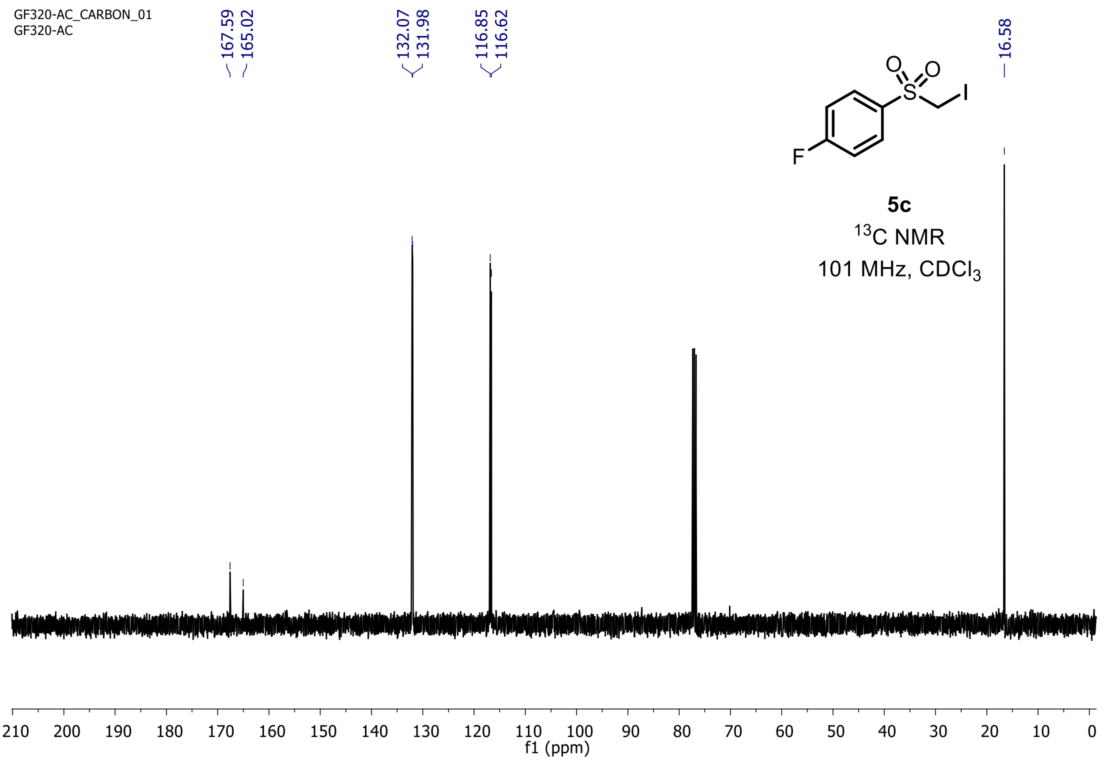
GF320-AC_CARBON_01
GF320-AC
167.59
165.02
132.07
131.98
116.85
116.62
16.58
5c
^{13}C NMR
101 MHz, CDCl$_3$
210 200 190 180 170 160 150 140 130 120 110 100 90 80 70 60 50 40 30 20 10 0
f1 (ppm)

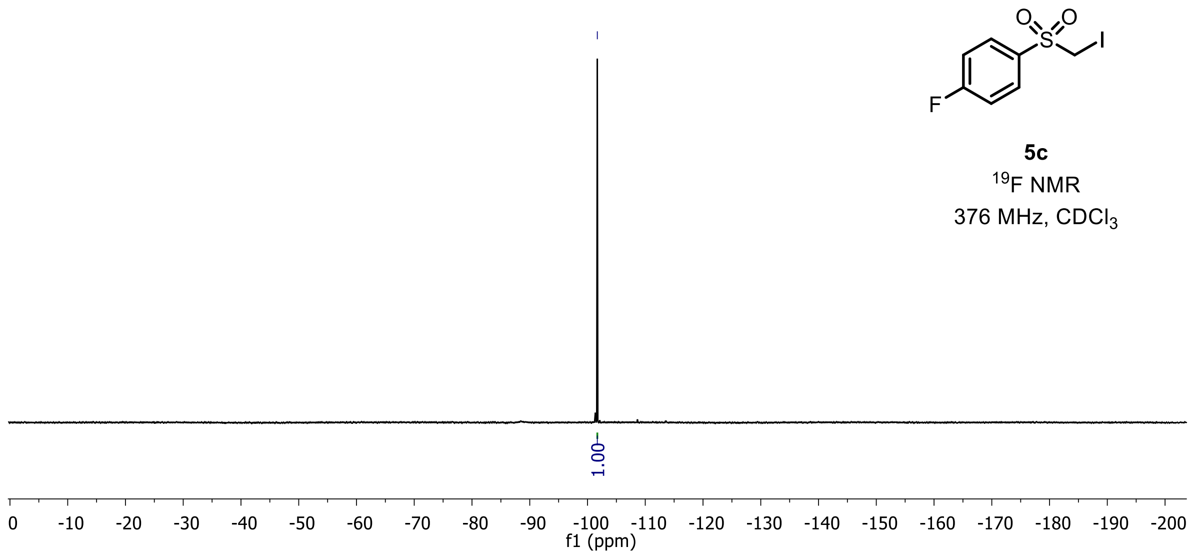
5c
^{19}F NMR
376 MHz, CDCl$_3$
1.00
0 -10 -20 -30 -40 -50 -60 -70 -80 -90 -100 -110 -120 -130 -140 -150 -160 -170 -180 -190 -200
f1 (ppm)

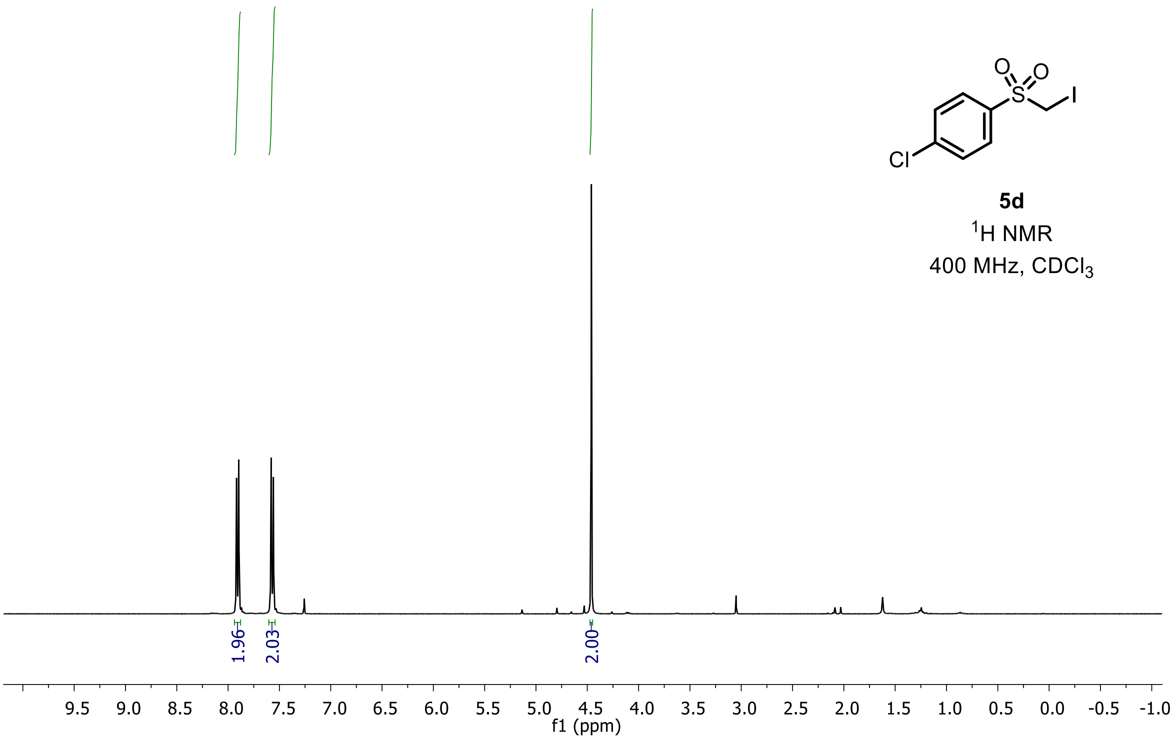

O
O
S
I
Cl
5d
^{1}H NMR
400 MHz, CDCl$_3$
1.96
2.03
2.00
9.5 9.0 8.5 8.0 7.5 7.0 6.5 6.0 5.5 5.0 4.5 4.0 3.5 3.0 2.5 2.0 1.5 1.0 0.5 0.0 -0.5 -1.0
f1 (ppm)

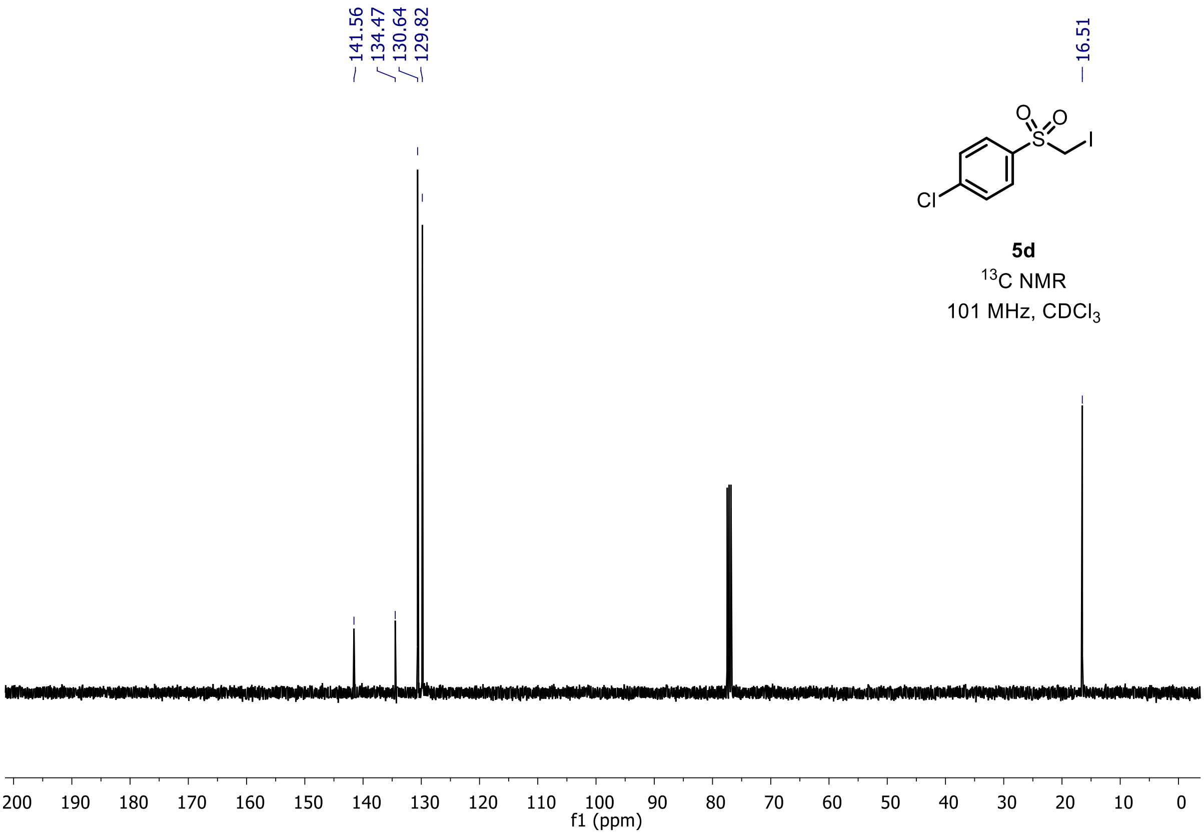

141.56
134.47
130.64
129.82
16.51
O
O
S
I
Cl
5d
^{13}C NMR
101 MHz, CDCl$_3$
200 190 180 170 160 150 140 130 120 110 100 90 80 70 60 50 40 30 20 10 0
f1 (ppm)

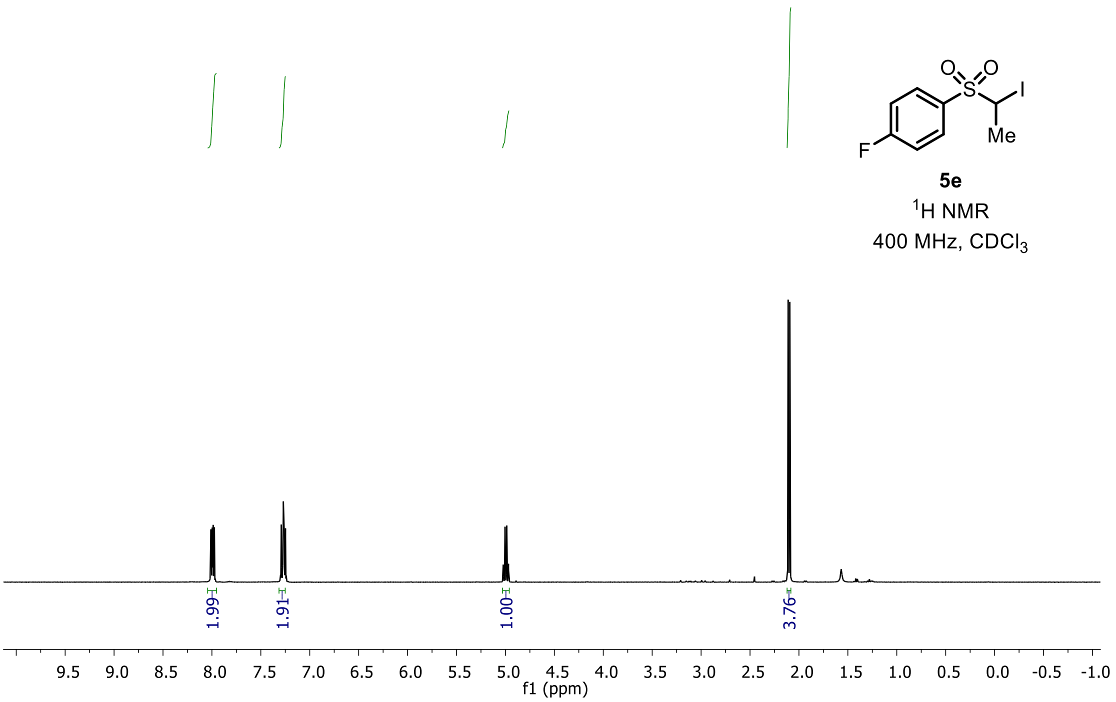
5e
1H NMR
400 MHz, CDCl3
1.99
1.91
1.00
3.76
9.5 9.0 8.5 8.0 7.5 7.0 6.5 6.0 5.5 5.0 4.5 4.0 3.5 3.0 2.5 2.0 1.5 1.0 0.5 0.0 -0.5 -1.0
f1 (ppm)

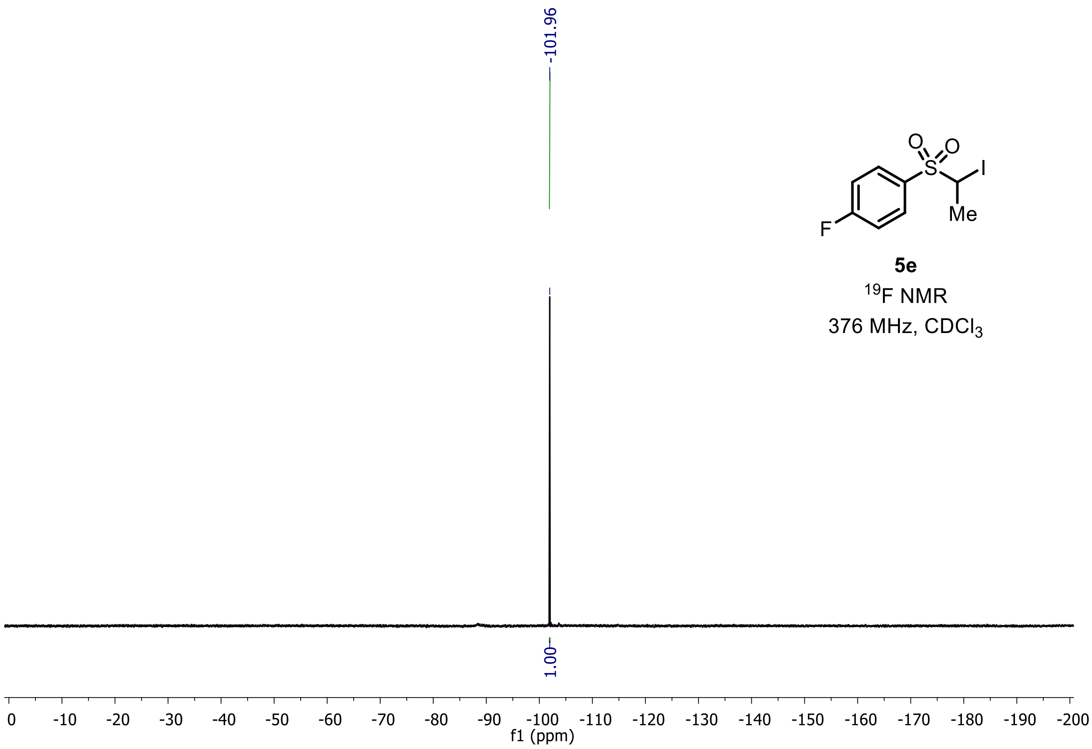
-101.96
5e
19F NMR
376 MHz, CDCl3
1.00
0 -10 -20 -30 -40 -50 -60 -70 -80 -90 -100 -110 -120 -130 -140 -150 -160 -170 -180 -190 -200
f1 (ppm)

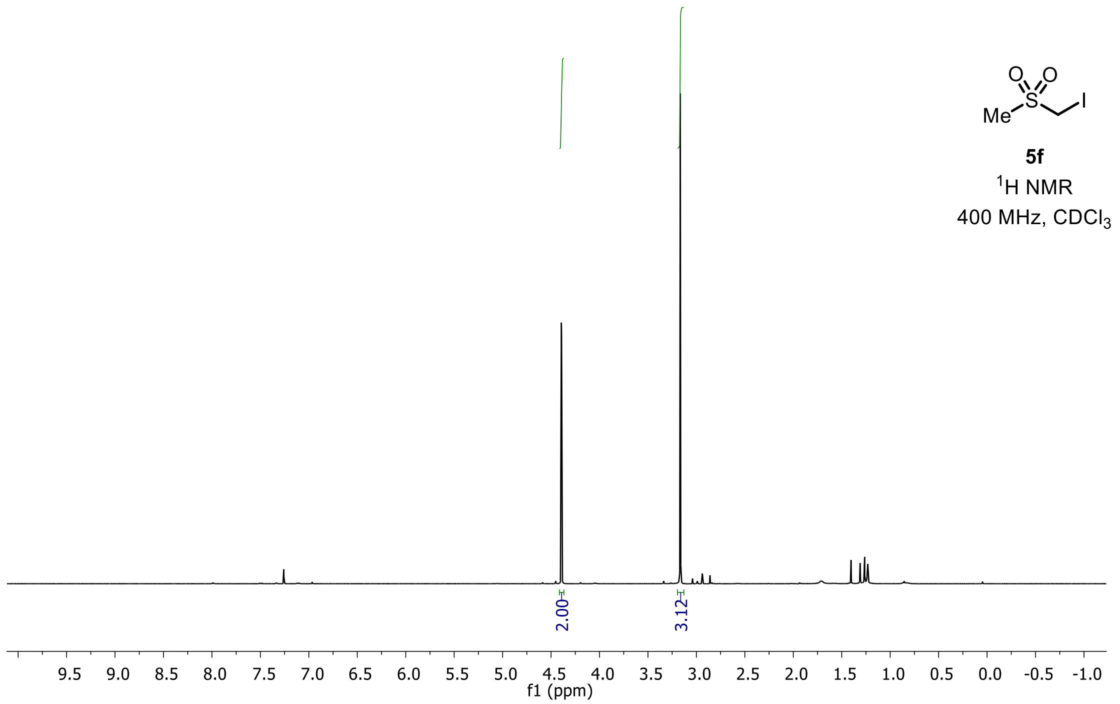
Me
5f
^{1}H NMR
400 MHz, CDCl$_3$
2.00
3.12
9.5 9.0 8.5 8.0 7.5 7.0 6.5 6.0 5.5 5.0 4.5 4.0 3.5 3.0 2.5 2.0 1.5 1.0 0.5 0.0 -0.5 -1.0
f1 (ppm)

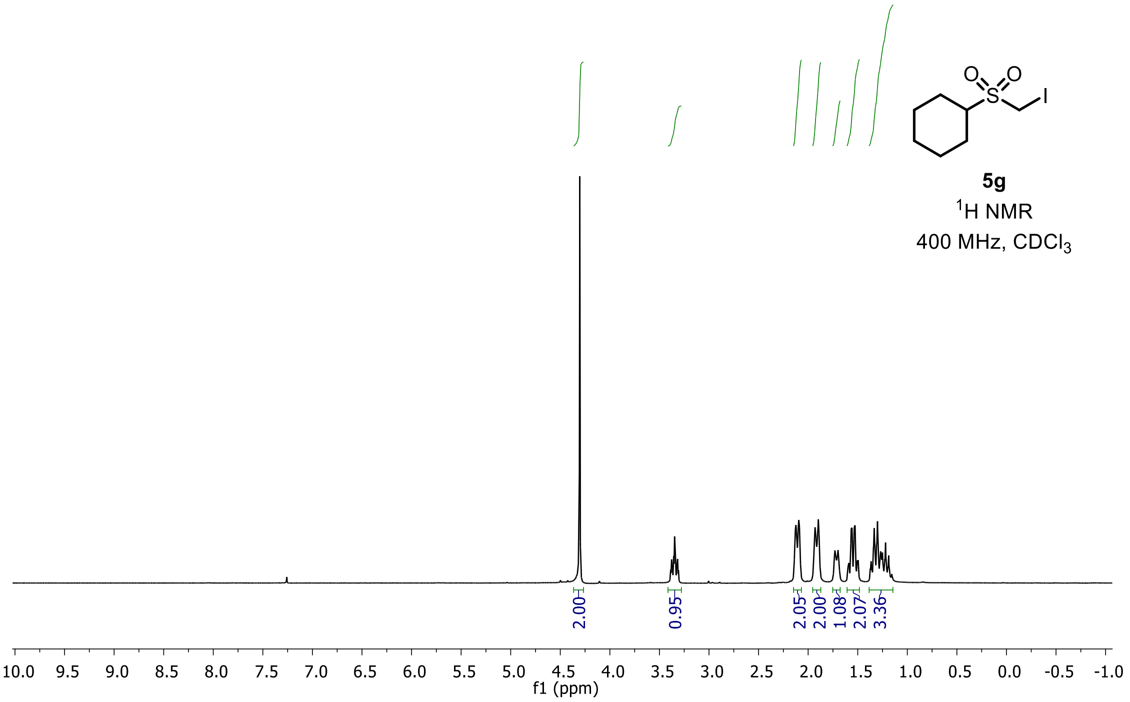
5g
^{1}H NMR
400 MHz, CDCl$_3$
2.00
0.95
2.05
2.00
1.08
2.07
3.36
10.0 9.5 9.0 8.5 8.0 7.5 7.0 6.5 6.0 5.5 5.0 4.5 4.0 3.5 3.0 2.5 2.0 1.5 1.0 0.5 0.0 -0.5 -1.0
f1 (ppm)

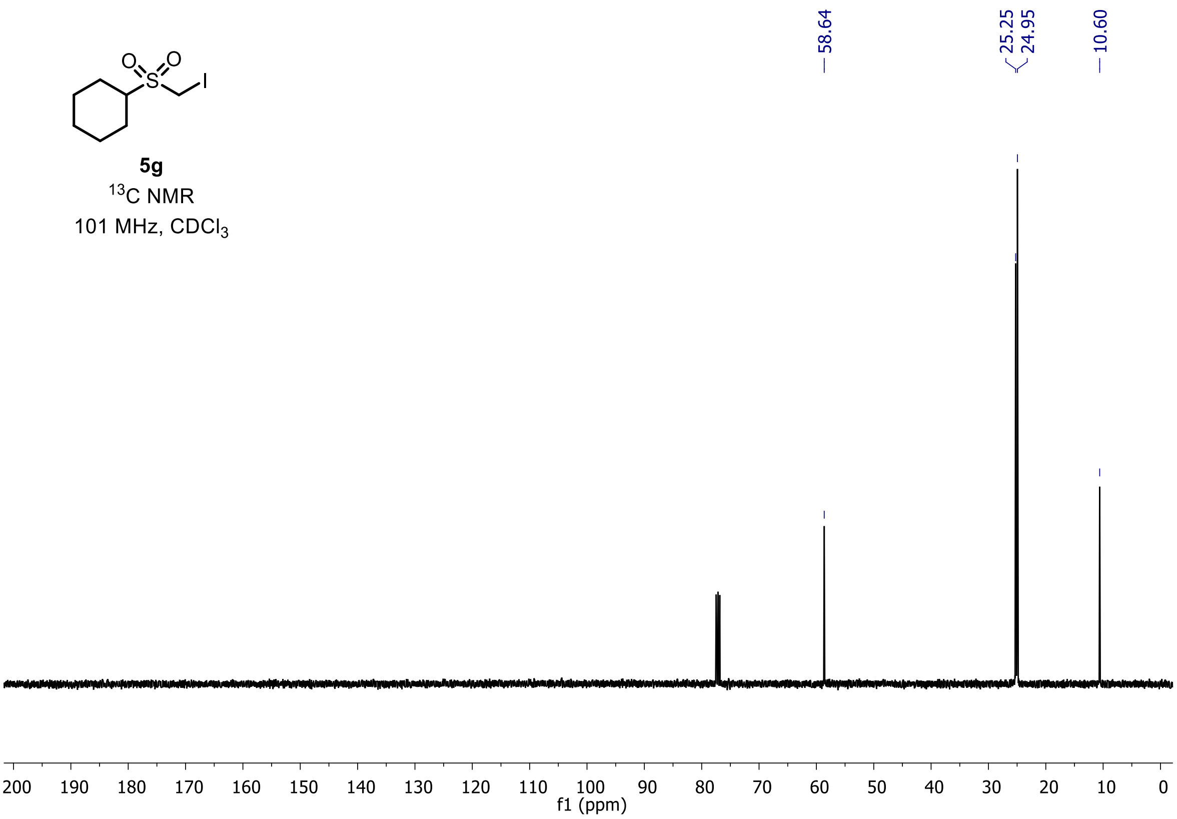

5g
^{13}C NMR
101 MHz, $CDCl_3$
58.64
25.25
24.95
10.60
200 190 180 170 160 150 140 130 120 110 100 90 80 70 60 50 40 30 20 10 0
f1 (ppm)

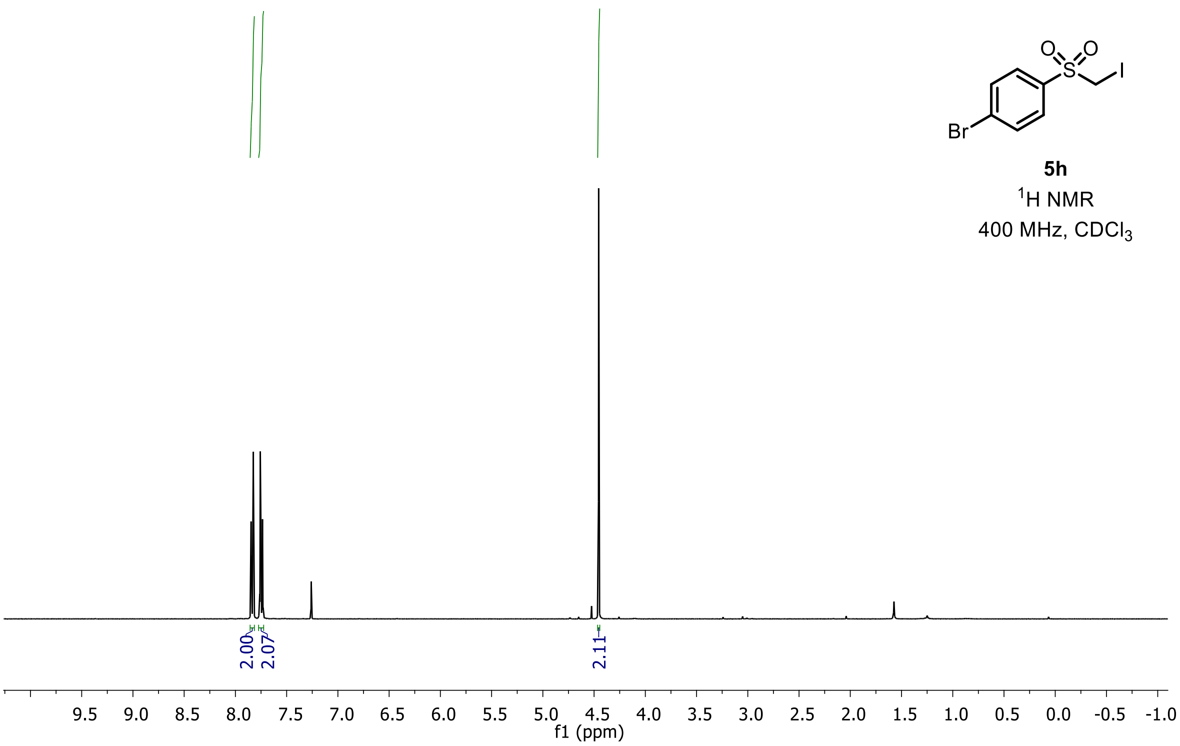

Br
5h
^{1}H NMR
400 MHz, $CDCl_3$
2.00
2.07
2.11
9.5 9.0 8.5 8.0 7.5 7.0 6.5 6.0 5.5 5.0 4.5 4.0 3.5 3.0 2.5 2.0 1.5 1.0 0.5 0.0 -0.5 -1.0
f1 (ppm)

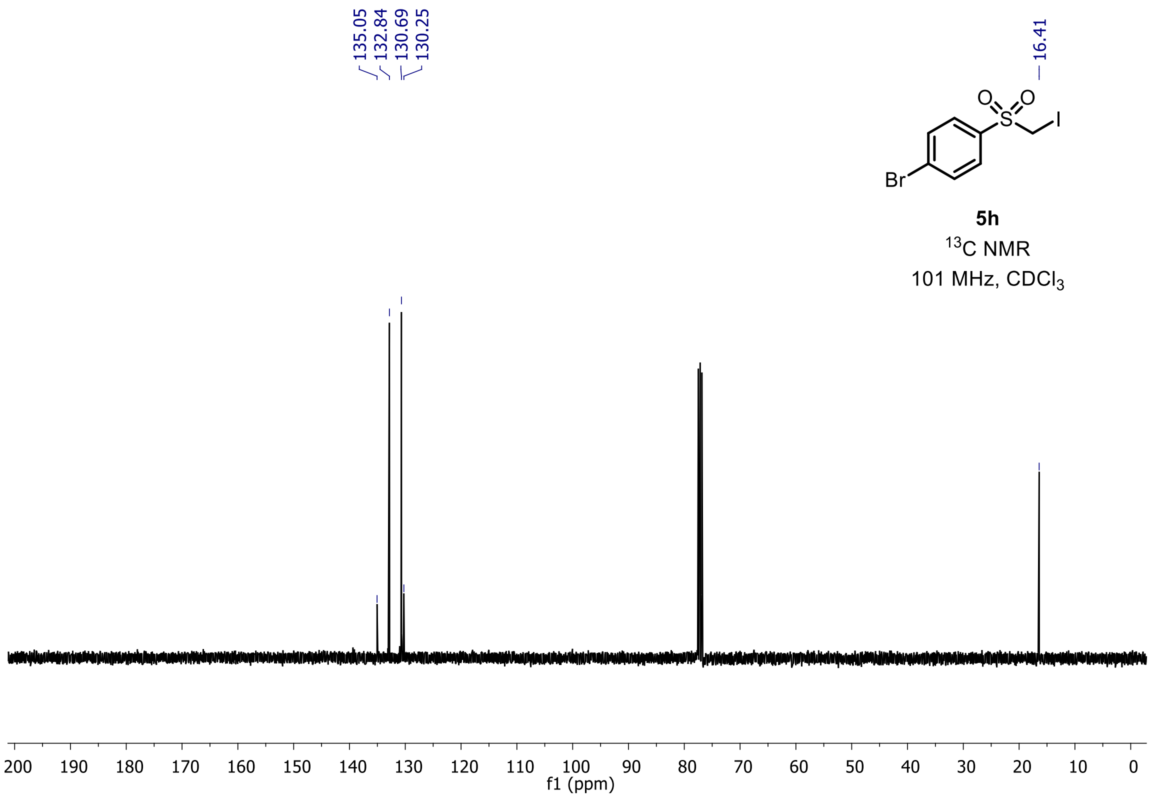

5h
^{13}C NMR
101 MHz, $CDCl_3$

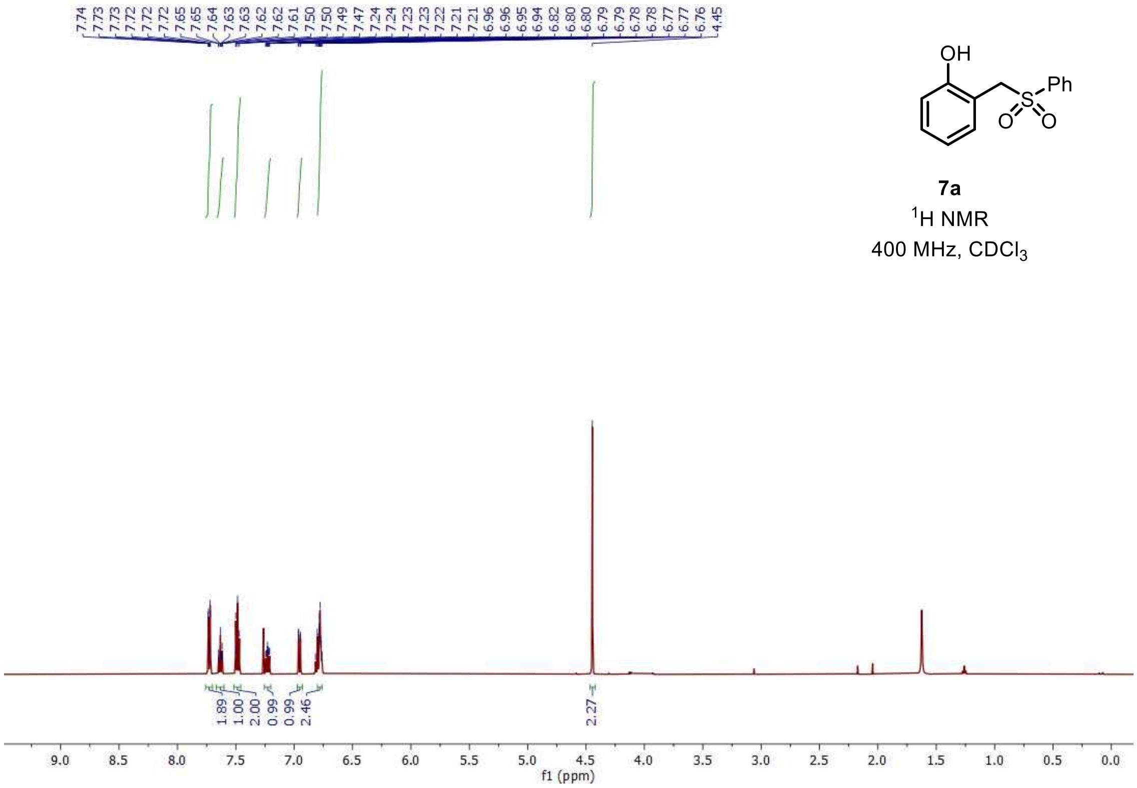

7a
^{1}H NMR
400 MHz, $CDCl_3$

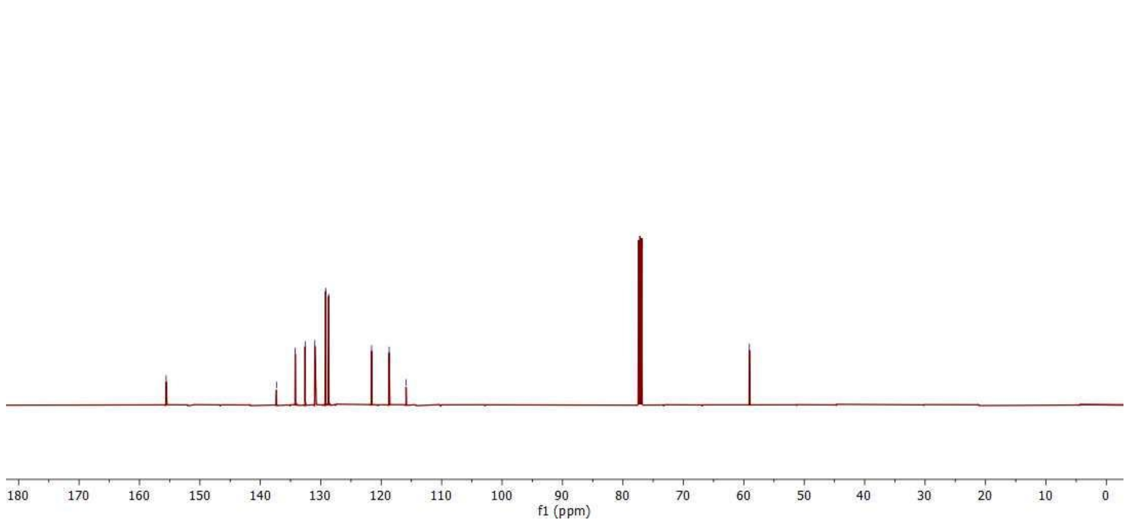

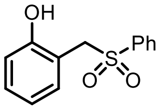

7a
^{13}C NMR
101 MHz, $CDCl_3$

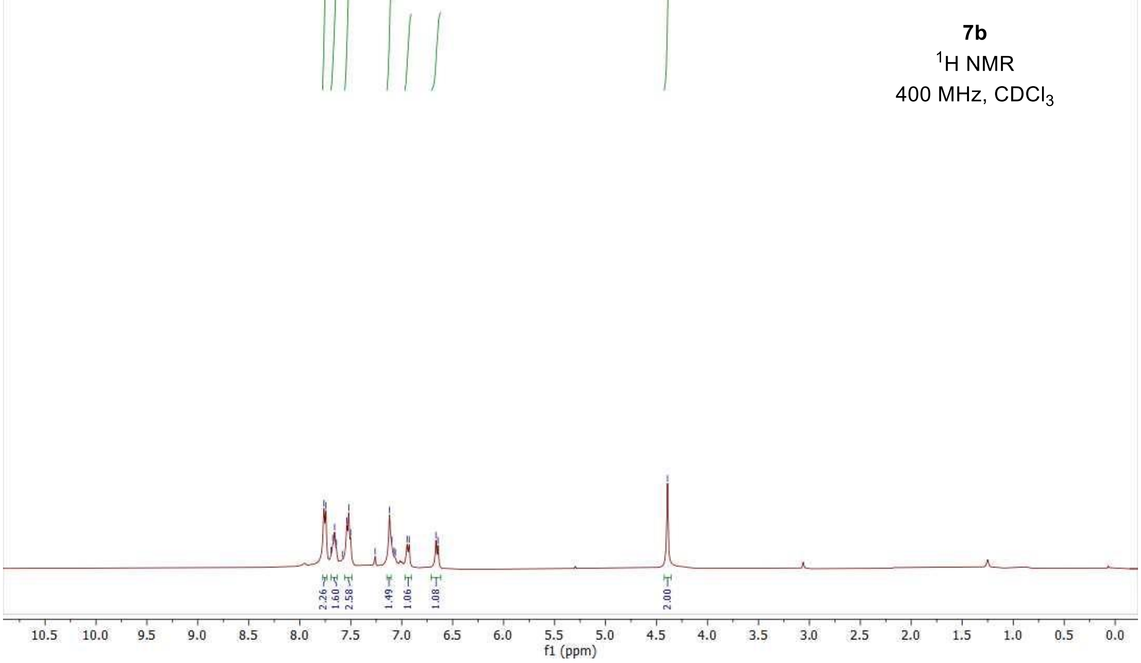

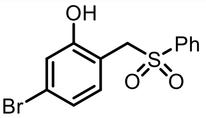

7b
^{1}H NMR
400 MHz, $CDCl_3$

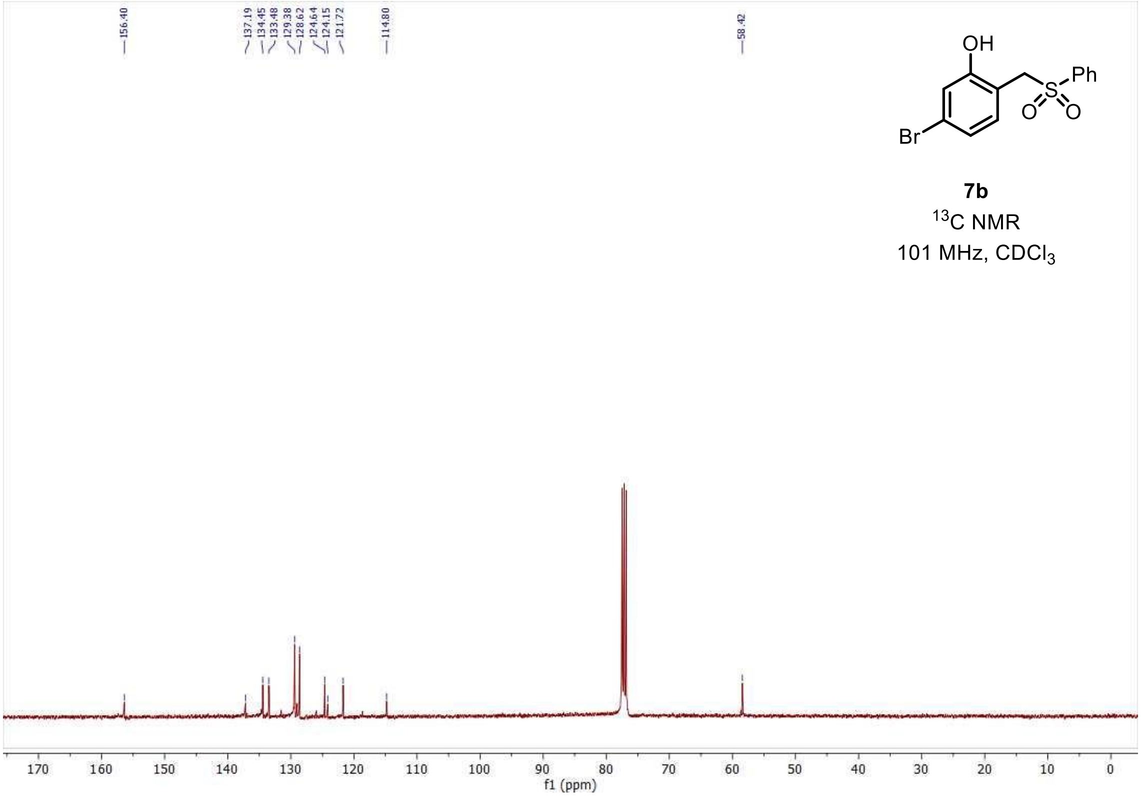
156.40
137.19
134.45
133.48
129.38
128.62
124.64
124.15
121.72
114.80
58.42
OH
Ph
S
O
O
Br
7b
^{13}C NMR
101 MHz, $CDCl_3$
170 160 150 140 130 120 110 100 90 80 70 60 50 40 30 20 10 0
f1 (ppm)

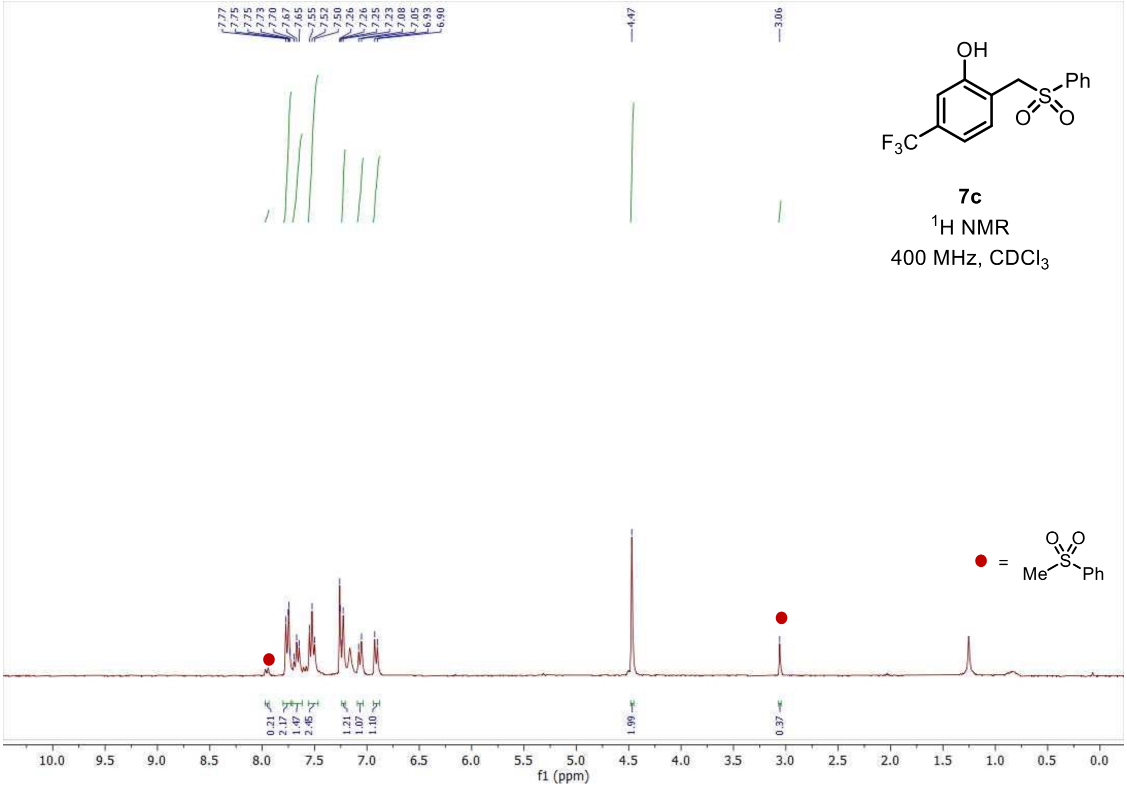
7.77
7.75
7.73
7.70
7.67
7.65
7.55
7.52
7.50
7.26
7.26
7.25
7.23
7.08
7.05
6.93
6.90
4.47
3.06
OH
Ph
S
O
O
F_3C
7c
^1H NMR
400 MHz, $CDCl_3$
=
O O
S
Me
Ph
0.21
2.17
1.47
2.45
1.21
1.07
1.10
1.99
0.37
10.0 9.5 9.0 8.5 8.0 7.5 7.0 6.5 6.0 5.5 5.0 4.5 4.0 3.5 3.0 2.5 2.0 1.5 1.0 0.5 0.0
f1 (ppm)

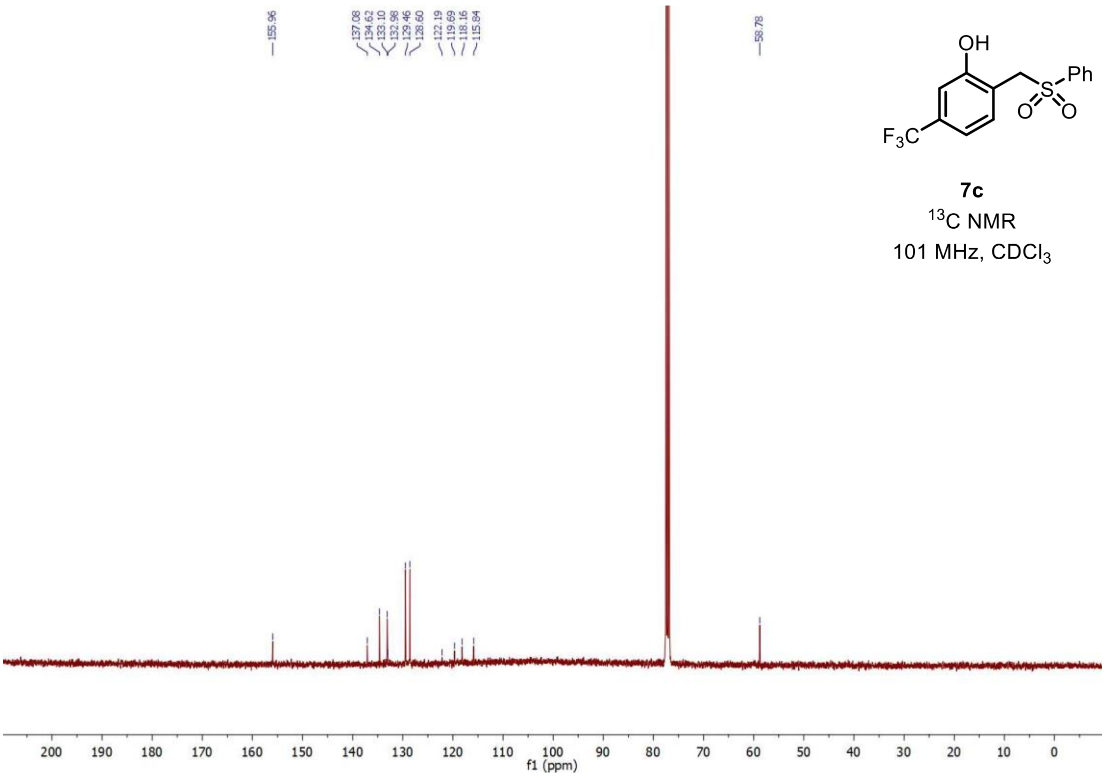

7c
^{13}C NMR
101 MHz, $CDCl_3$

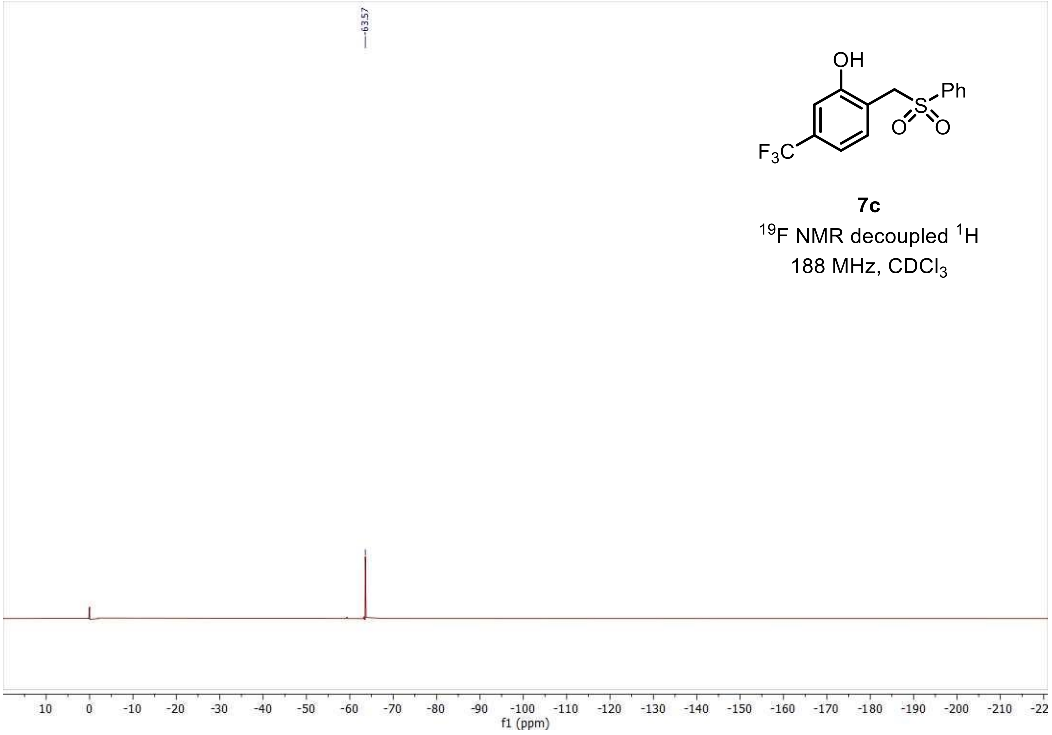

7c
^{19}F NMR decoupled ^{1}H
188 MHz, $CDCl_3$

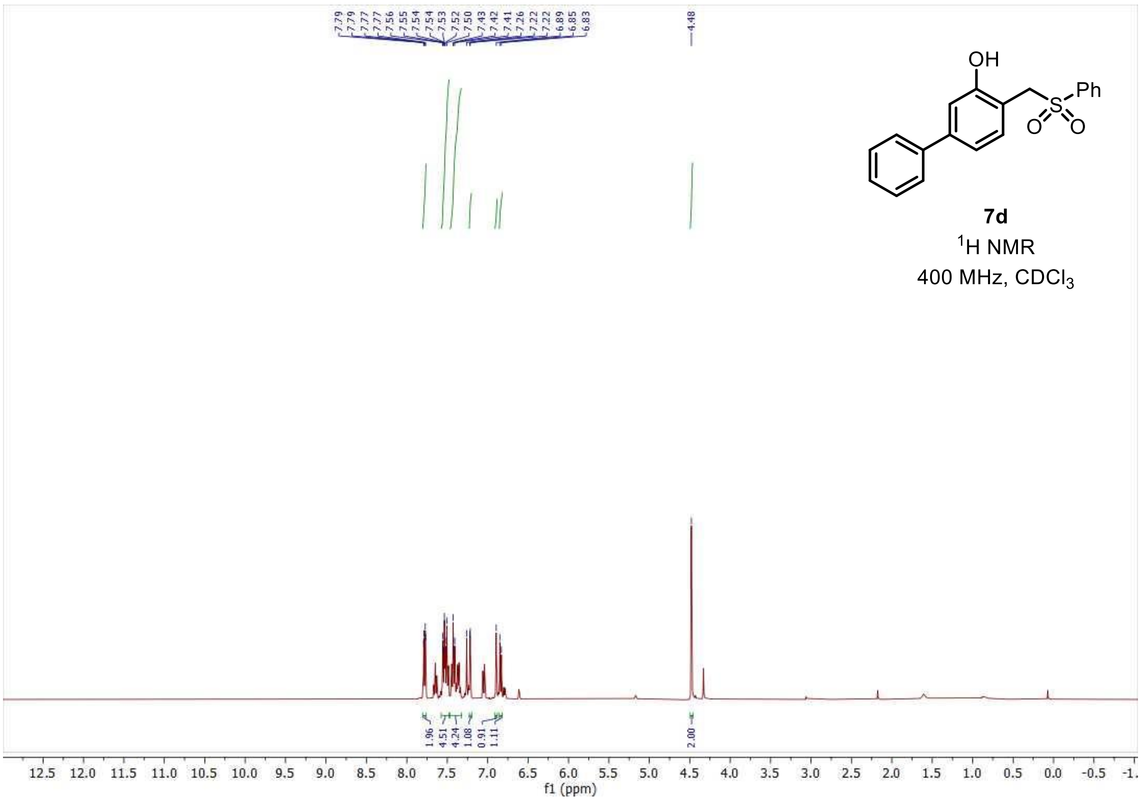

7d
^{1}H NMR
400 MHz, $CDCl_3$

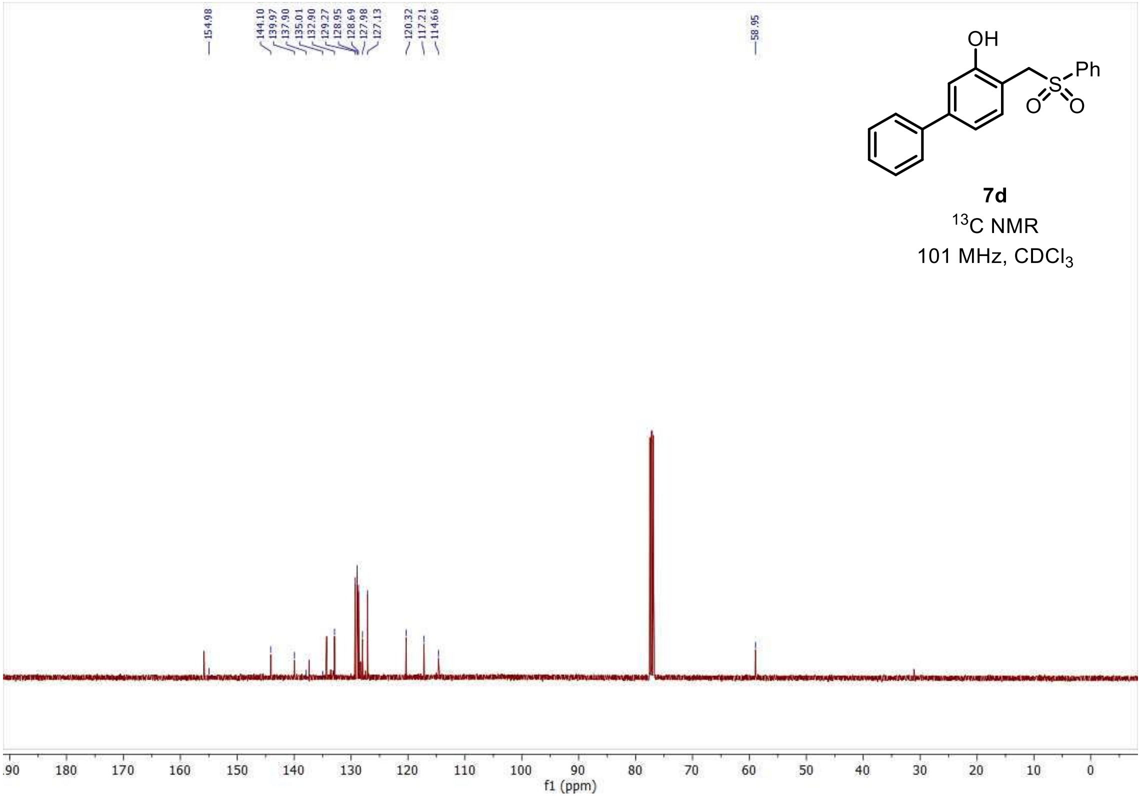

7d
^{13}C NMR
101 MHz, $CDCl_3$

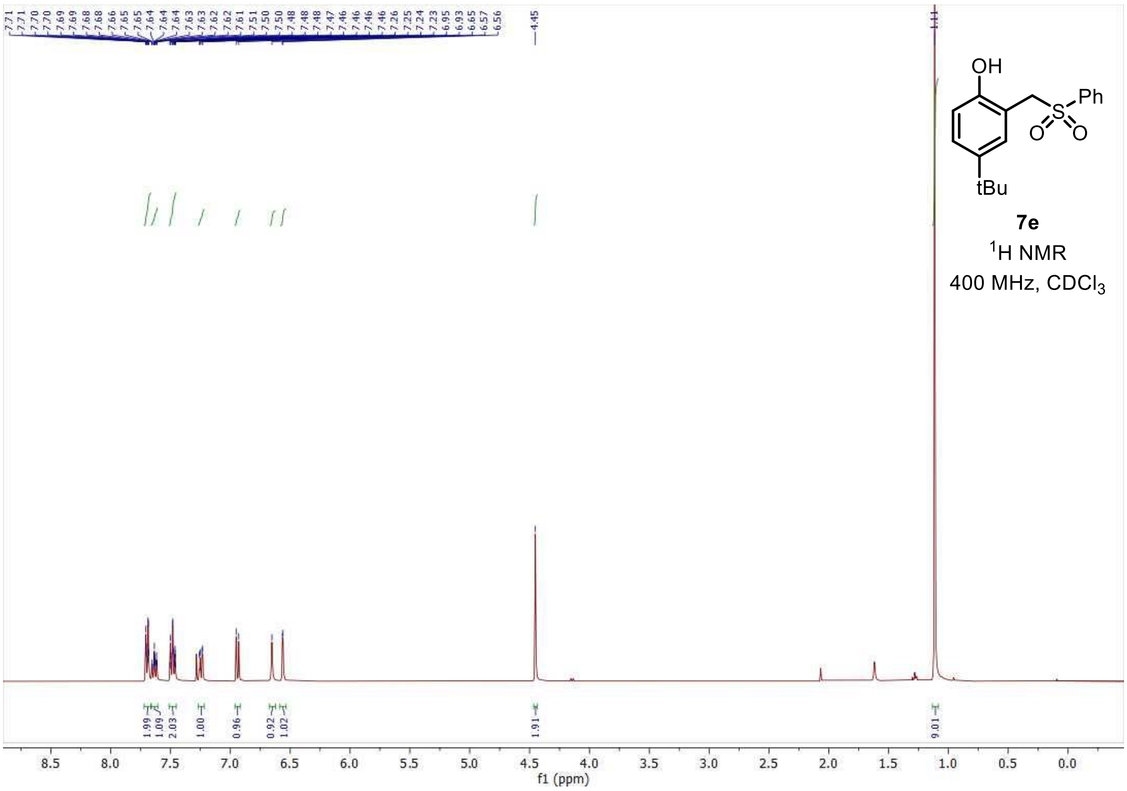
OH
Ph
S
O
O
tBu
7e
^{1}H NMR
400 MHz, CDCl$_3$
f1 (ppm)

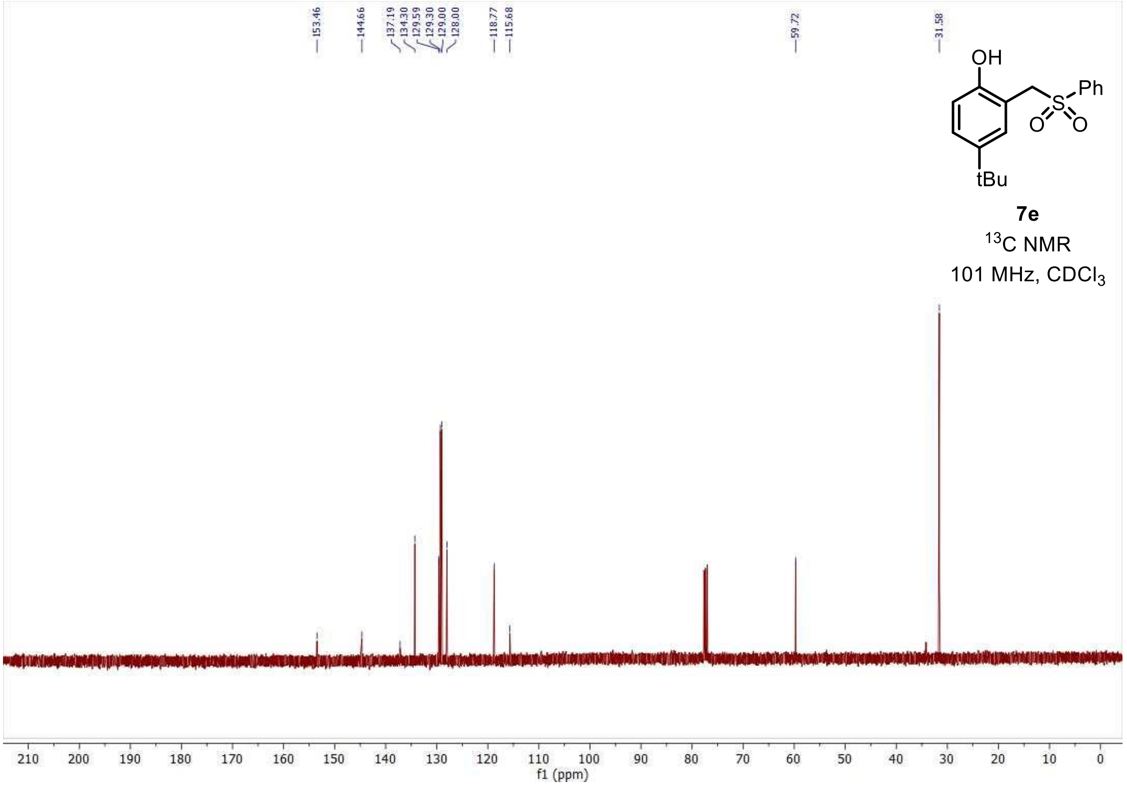
OH
Ph
S
O
O
tBu
7e
^{13}C NMR
101 MHz, CDCl$_3$
f1 (ppm)

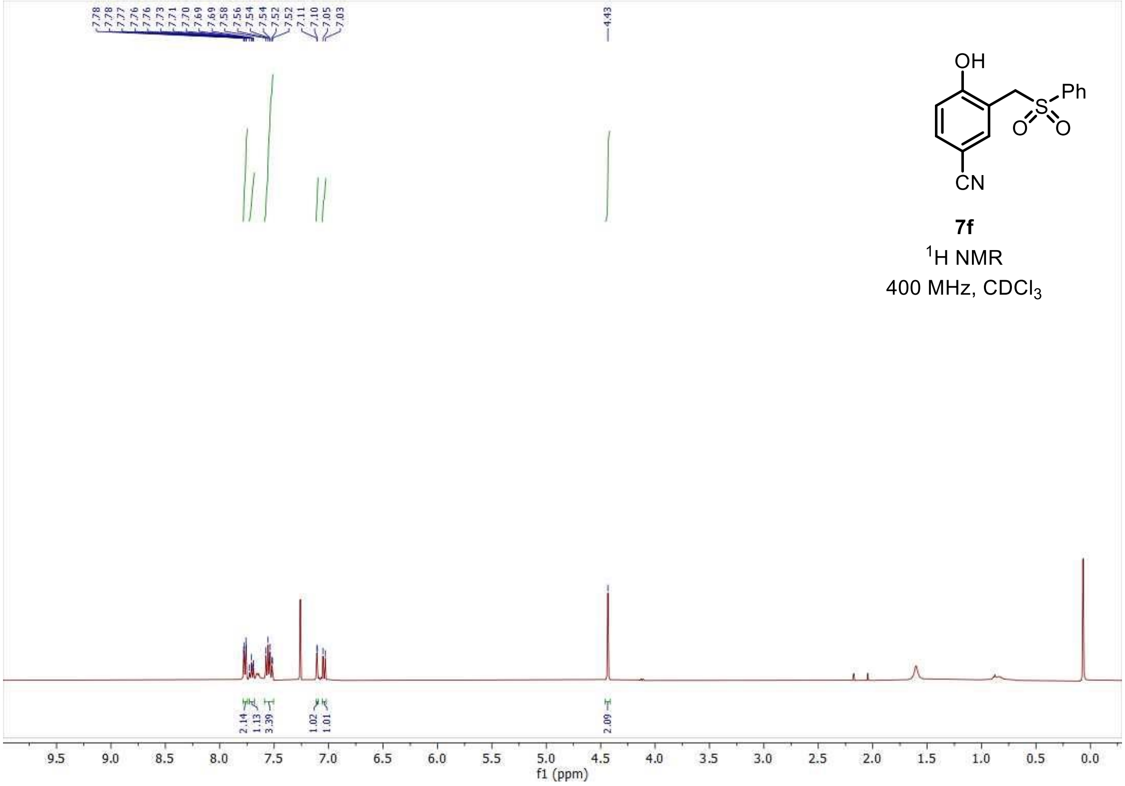

7.78
7.78
7.77
7.76
7.76
7.73
7.71
7.70
7.69
7.69
7.58
7.56
7.54
7.54
7.52
7.52
7.11
7.10
7.05
7.03
4.43
OH
Ph
S
O
O
CN
7f
^{1}H NMR
400 MHz, $CDCl_3$
2.14
1.13
3.39
1.02
1.01
2.09
9.5
9.0
8.5
8.0
7.5
7.0
6.5
6.0
5.5
5.0
4.5
4.0
3.5
3.0
2.5
2.0
1.5
1.0
0.5
0.0
f1 (ppm)

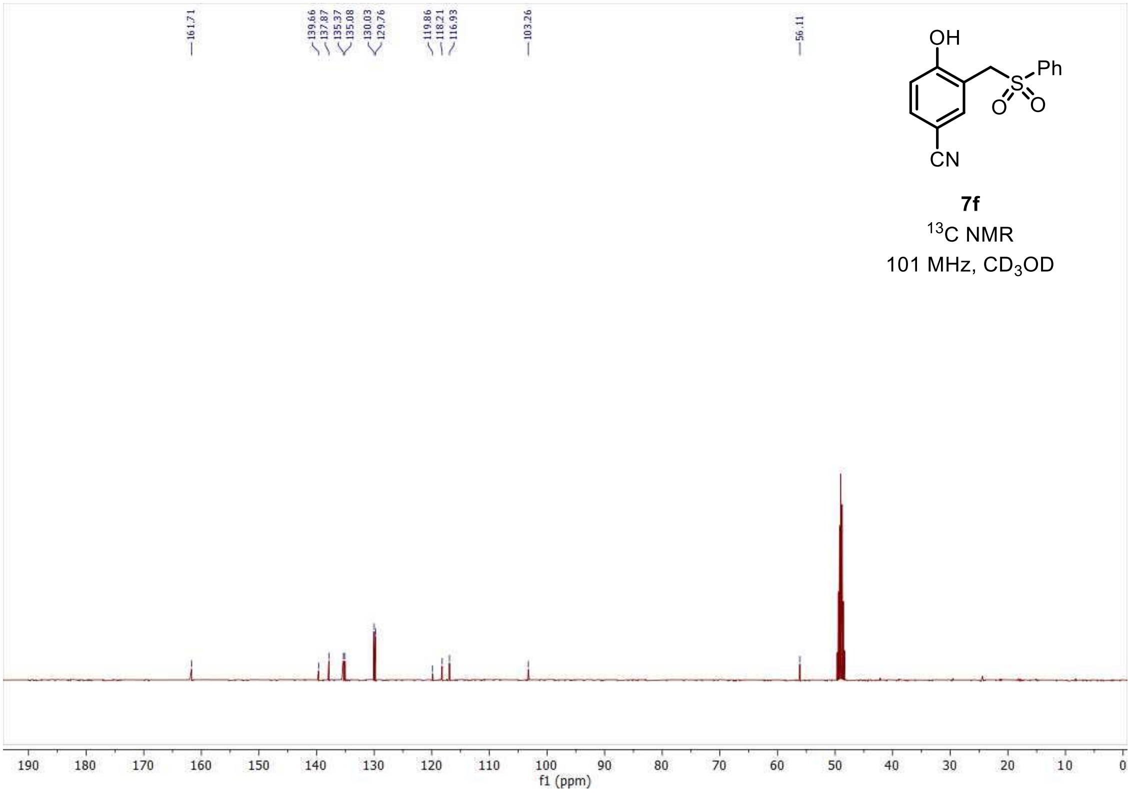
161.71
139.66
137.87
135.37
135.08
130.03
129.76
119.86
118.21
116.93
103.26
56.11
OH
Ph
O
S
O
CN
7f
^{13}C NMR
101 MHz, CD_3OD
190 180 170 160 150 140 130 120 110 100 90 80 70 60 50 40 30 20 10 0
f1 (ppm)

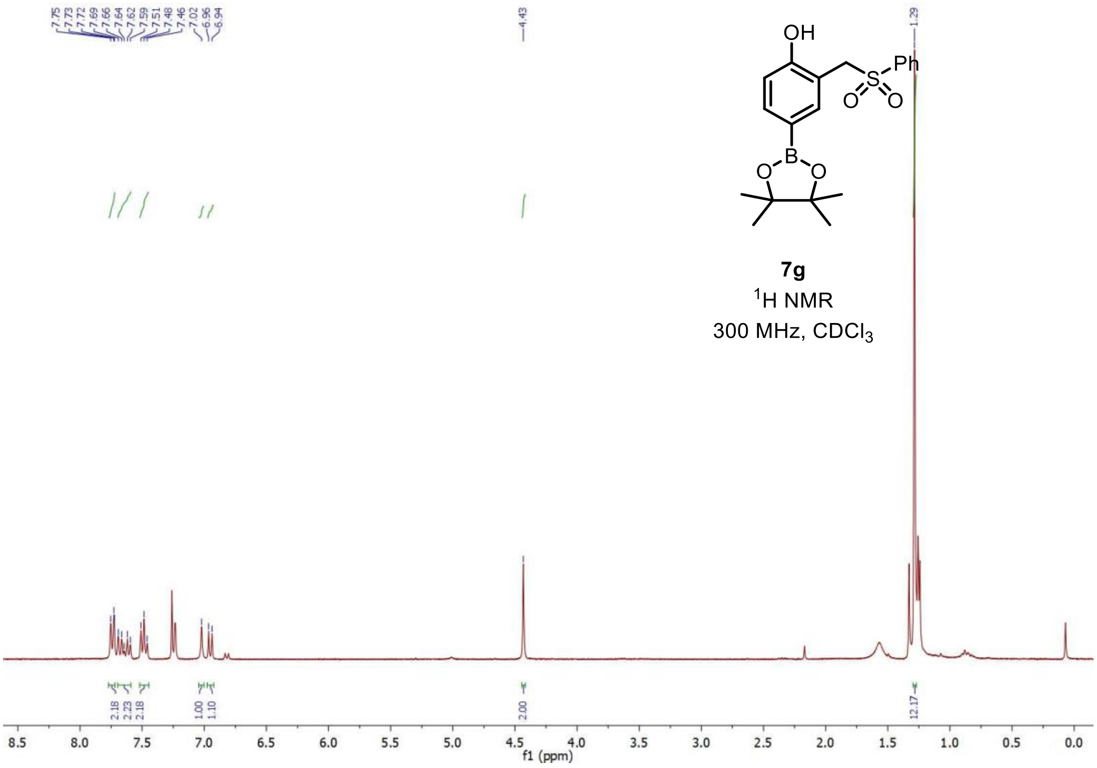
7.75
7.73
7.72
7.69
7.66
7.64
7.62
7.59
7.51
7.48
7.46
7.02
6.96
6.94
4.43
1.29
OH
Ph
O
S
O
B
O
O
7g
^{1}H NMR
300 MHz, $CDCl_3$
2.18
2.23
2.18
1.00
1.10
2.00
12.17
8.5 8.0 7.5 7.0 6.5 6.0 5.5 5.0 4.5 4.0 3.5 3.0 2.5 2.0 1.5 1.0 0.5 0.0
f1 (ppm)

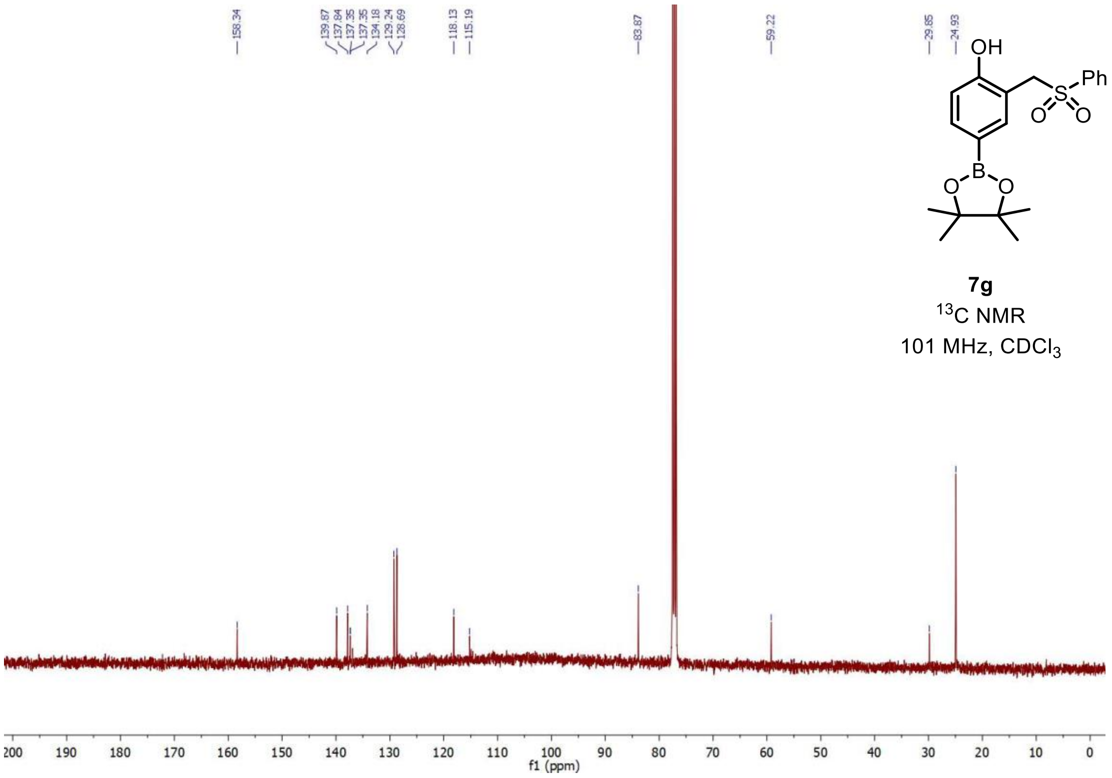

7g
^{13}C NMR
101 MHz, $CDCl_3$

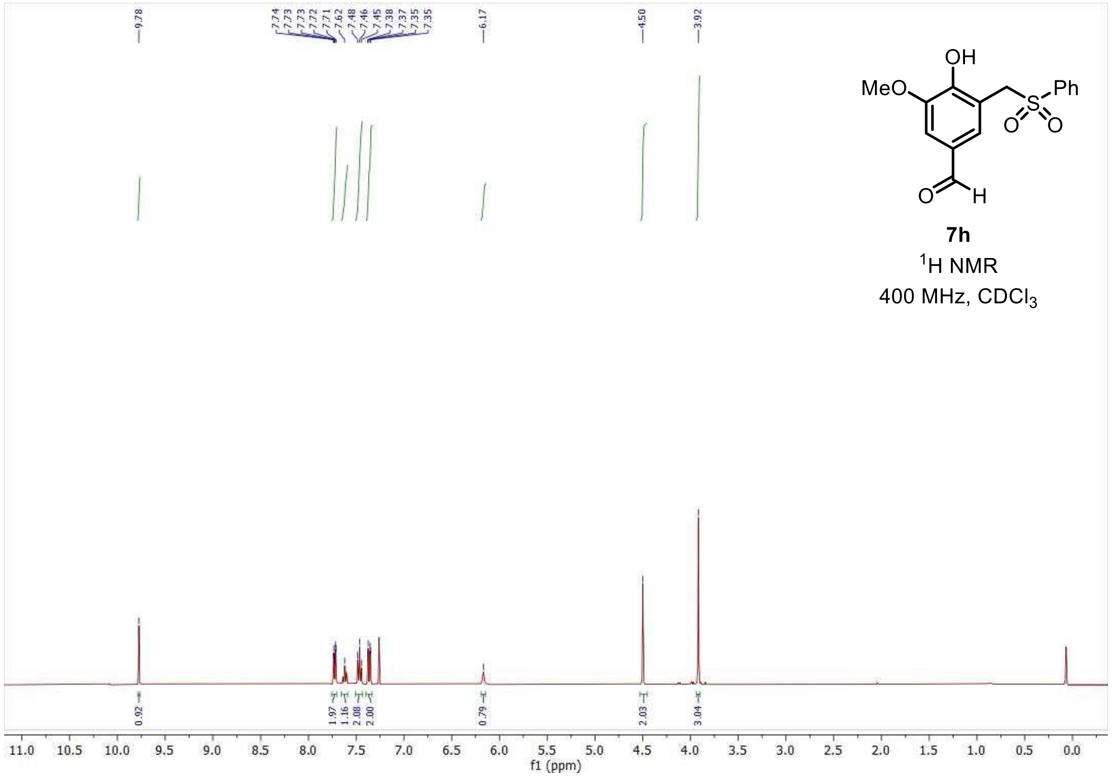

7h
^{1}H NMR
400 MHz, $CDCl_3$

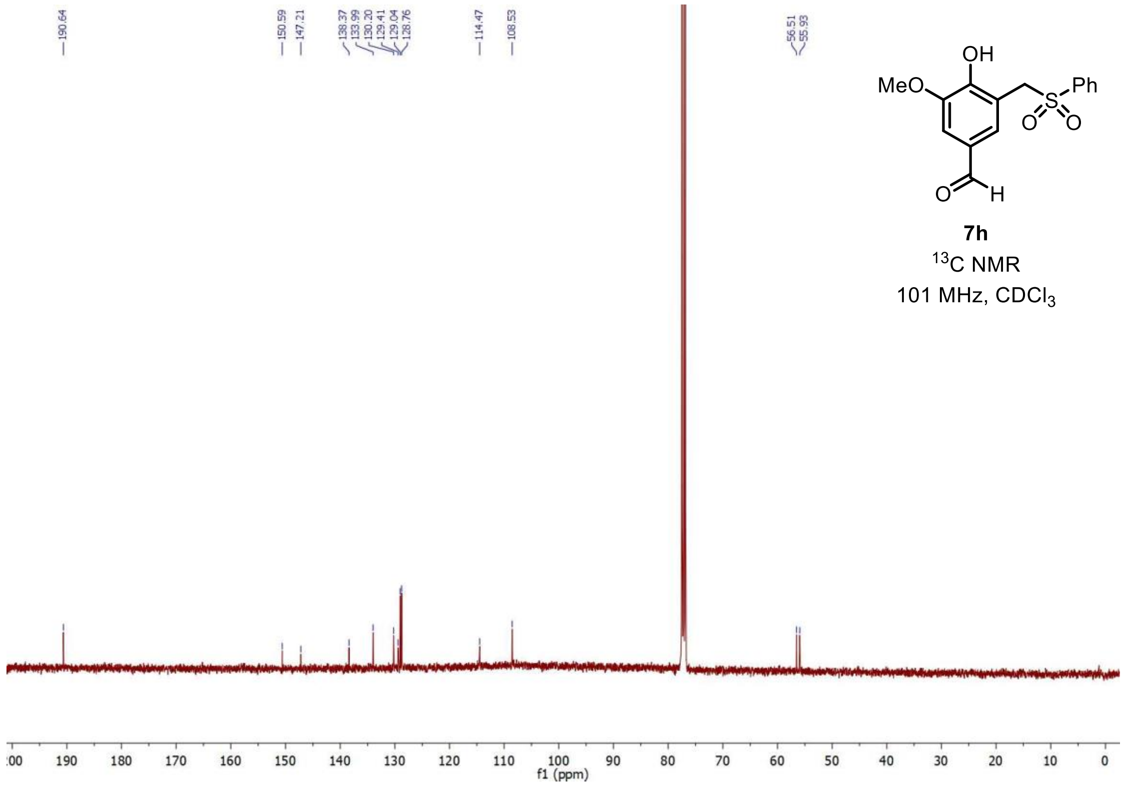

190.64
150.59
147.21
138.37
133.99
130.20
129.41
129.04
128.76
114.47
108.53
56.51
55.93
MeO
OH
Ph
S
O
O
H
7h
^{13}C NMR
101 MHz, $CDCl_3$
f1 (ppm)

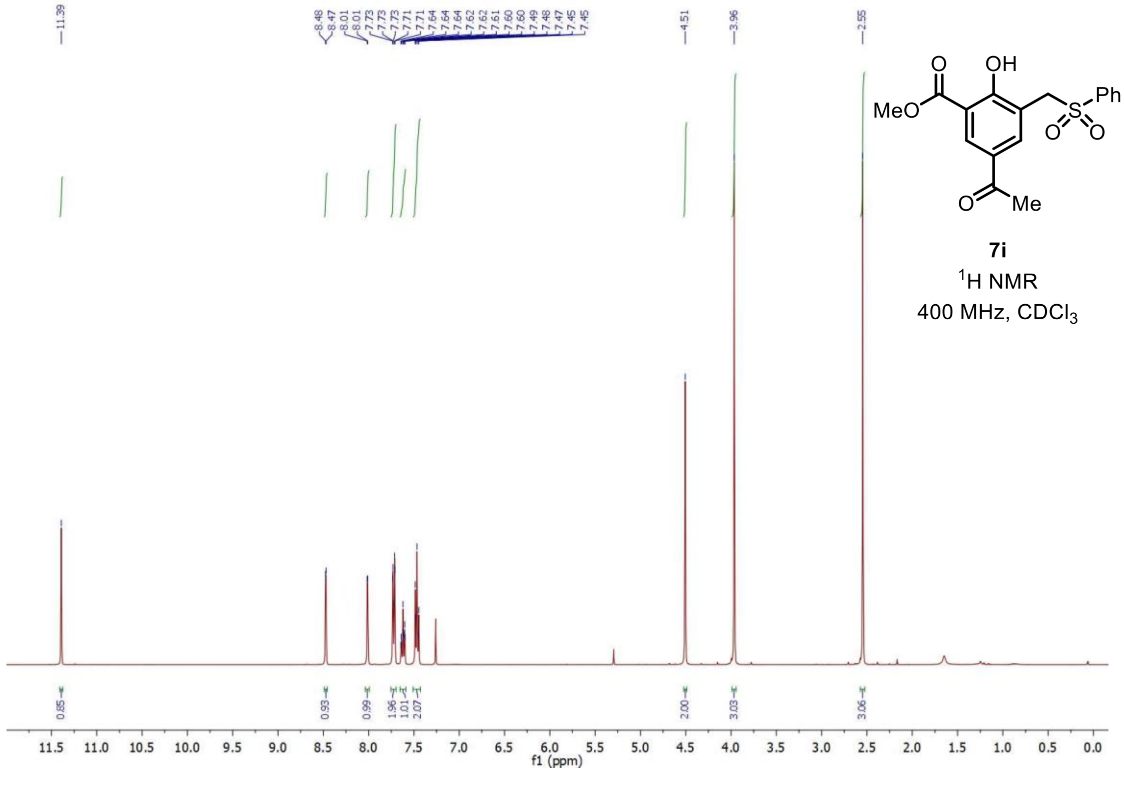

11.39
8.48
8.47
8.01
8.01
7.73
7.73
7.71
7.71
7.64
7.64
7.62
7.61
7.60
7.49
7.48
7.47
7.45
7.45
4.51
3.96
2.55
0.85
0.93
0.99
1.96
1.01
2.07
2.00
3.03
3.06
MeO
OH
Ph
S
O
O
Me
7i
^{1}H NMR
400 MHz, $CDCl_3$
f1 (ppm)

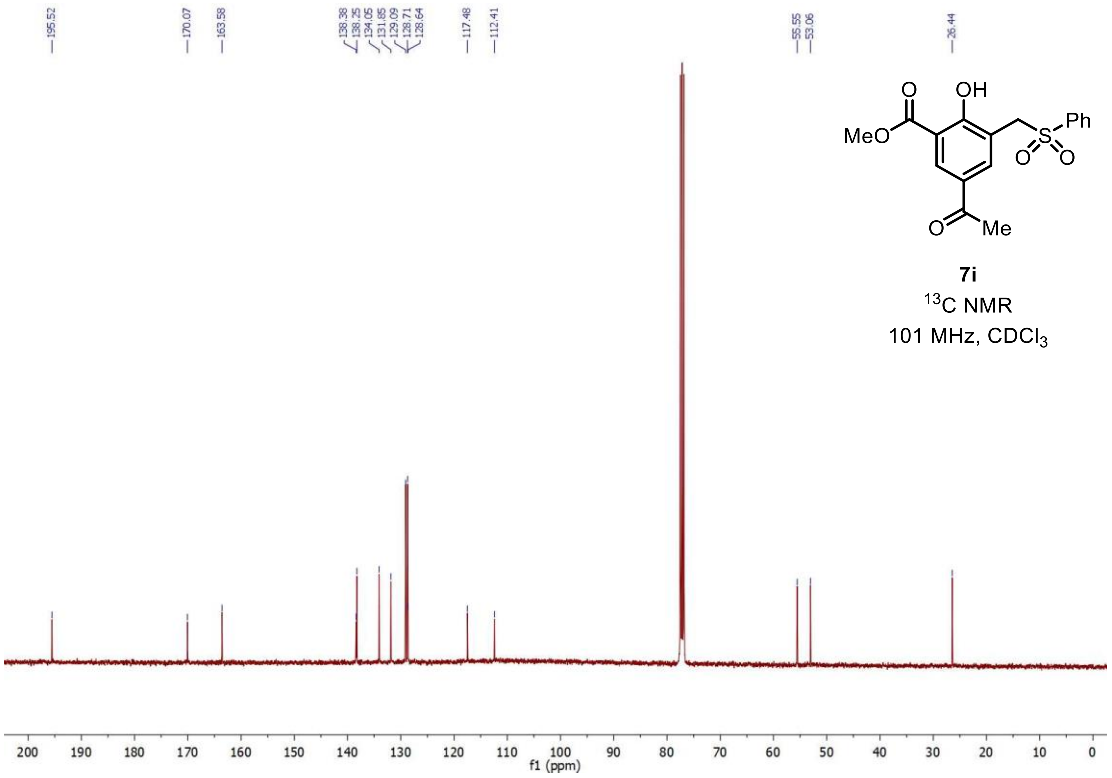
7i
^{13}C NMR
101 MHz, CDCl$_3$
f1 (ppm)

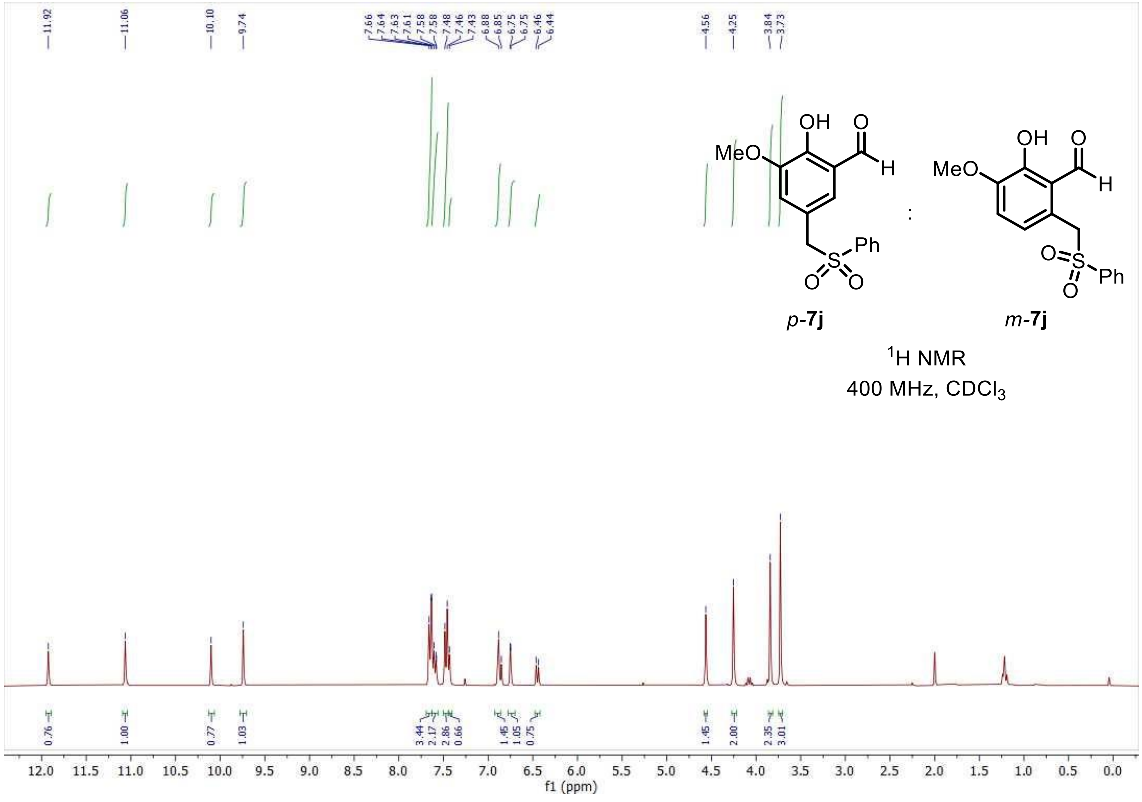
p-7j
m-7j
^{1}H NMR
400 MHz, CDCl$_3$
f1 (ppm)

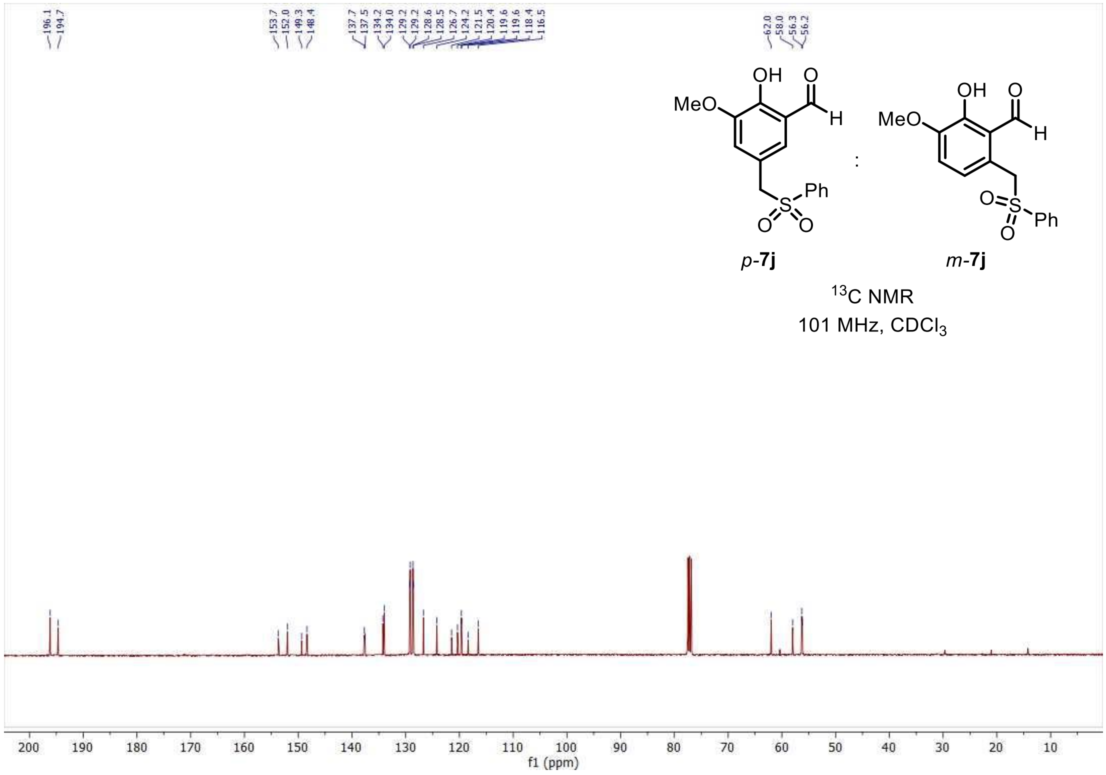

MeO
OH
H
Ph
p-**7j**
m-**7j**
^{13}C NMR
101 MHz, $CDCl_3$
f1 (ppm)

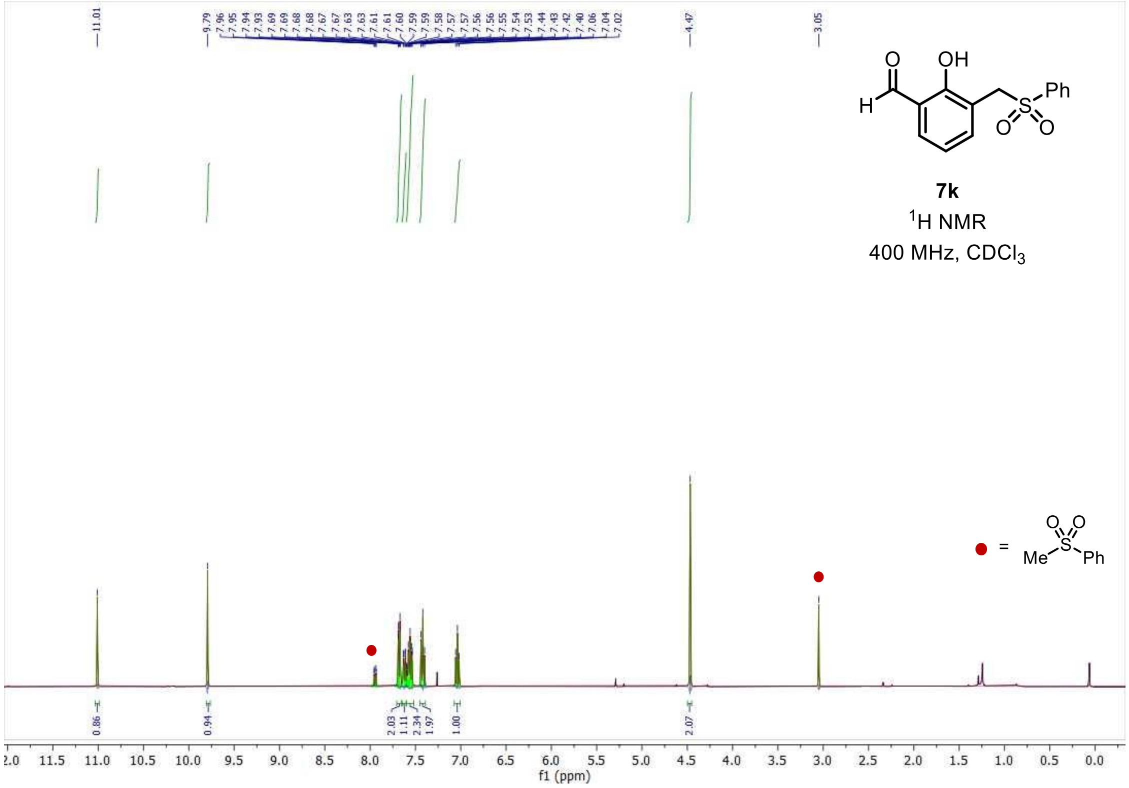

OH
H
Ph
7k
^{1}H NMR
400 MHz, $CDCl_3$
Me
f1 (ppm)

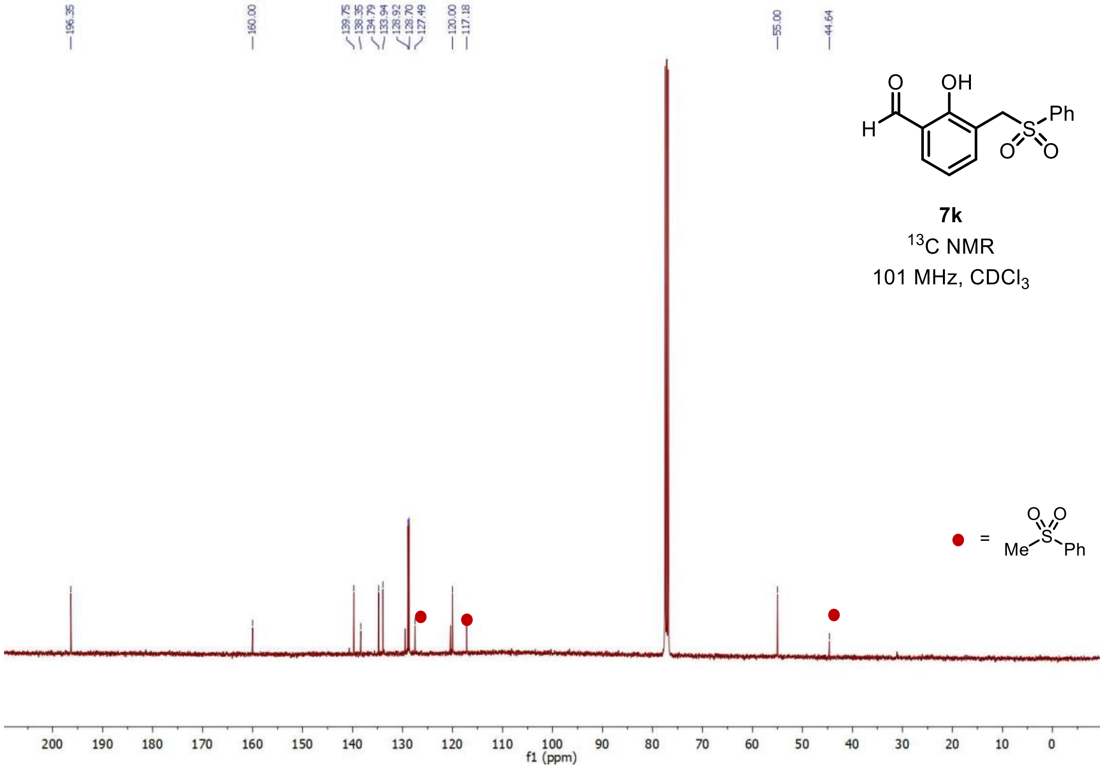
196.35
160.00
139.75
138.35
134.79
133.94
128.92
128.70
127.49
120.00
117.18
55.00
44.64
O
OH
H
Ph
S
O
O
7k
^{13}C NMR
101 MHz, $CDCl_3$
= Me S Ph O O
f1 (ppm)

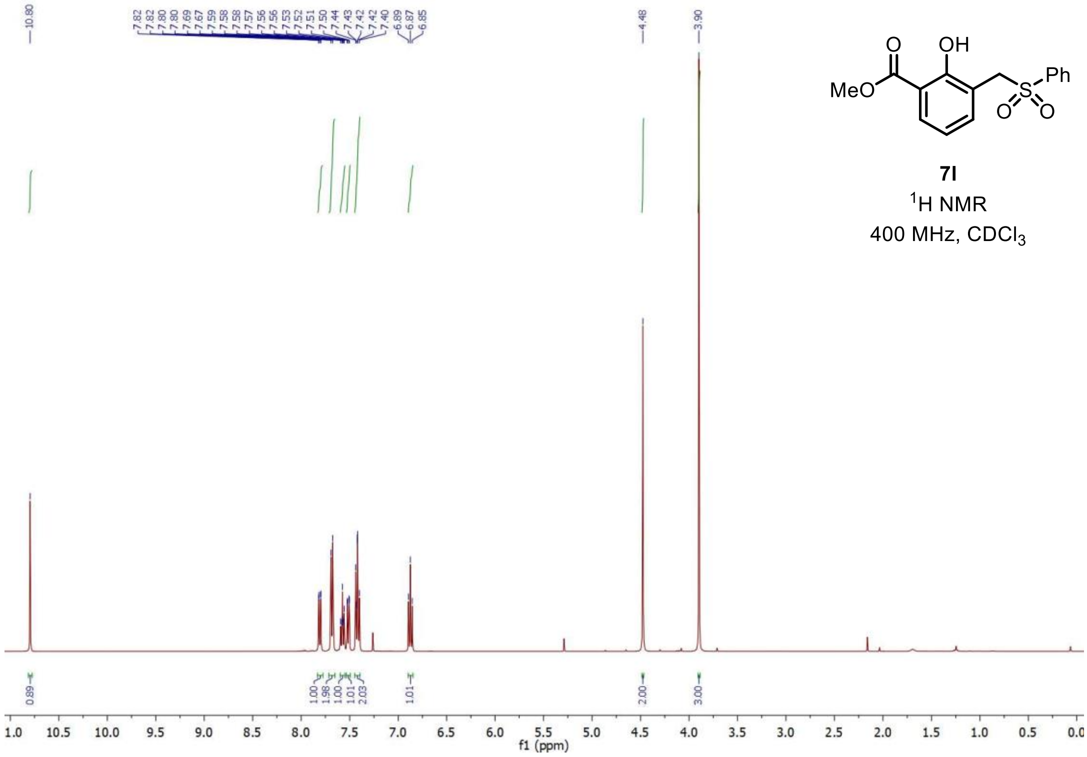
10.80
7.82
7.82
7.80
7.80
7.69
7.67
7.59
7.58
7.58
7.57
7.56
7.56
7.53
7.52
7.51
7.50
7.44
7.43
7.42
7.42
7.40
6.89
6.87
6.85
4.48
3.90
O
OH
MeO
Ph
S
O
O
7l
^{1}H NMR
400 MHz, $CDCl_3$
0.89
1.00
1.98
1.00
1.01
2.03
1.01
2.00
3.00
f1 (ppm)

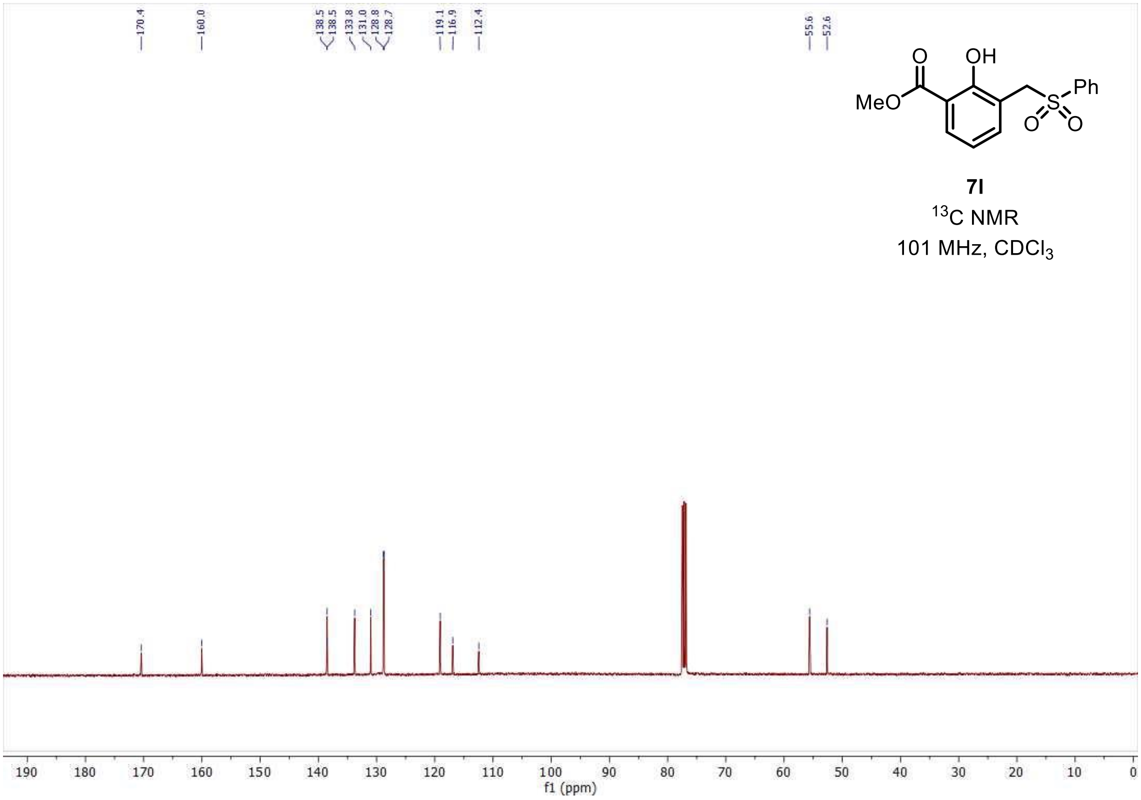
170.4
160.0
138.5
138.5
133.8
131.0
128.8
128.7
119.1
116.9
112.4
55.6
52.6
O
OH
MeO
S
Ph
O
O
7l
13C NMR
101 MHz, CDCl3
f1 (ppm)

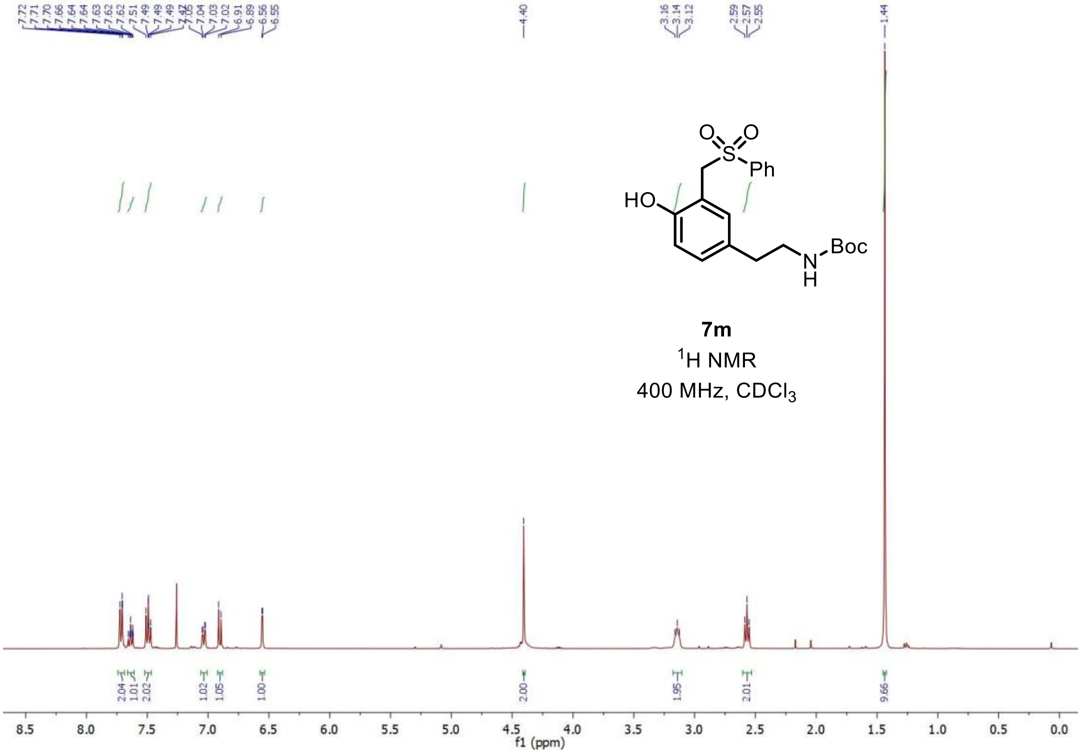
O
O
S
Ph
HO
N
Boc
H
7m
1H NMR
400 MHz, CDCl3
f1 (ppm)

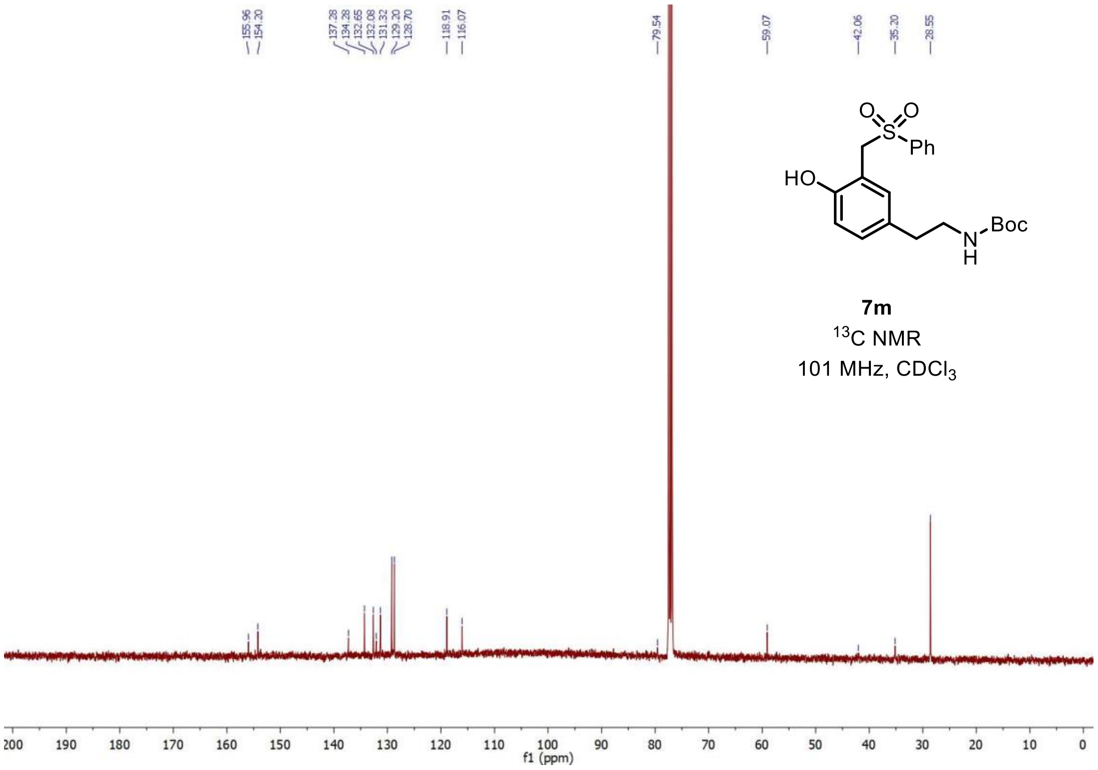
155.96
154.20
137.28
134.28
132.65
132.08
131.32
129.20
128.70
118.91
116.07
79.54
59.07
42.06
35.20
28.55
HO
S
O O
Ph
N
H
Boc
7m
^{13}C NMR
101 MHz, $CDCl_3$
f1 (ppm)

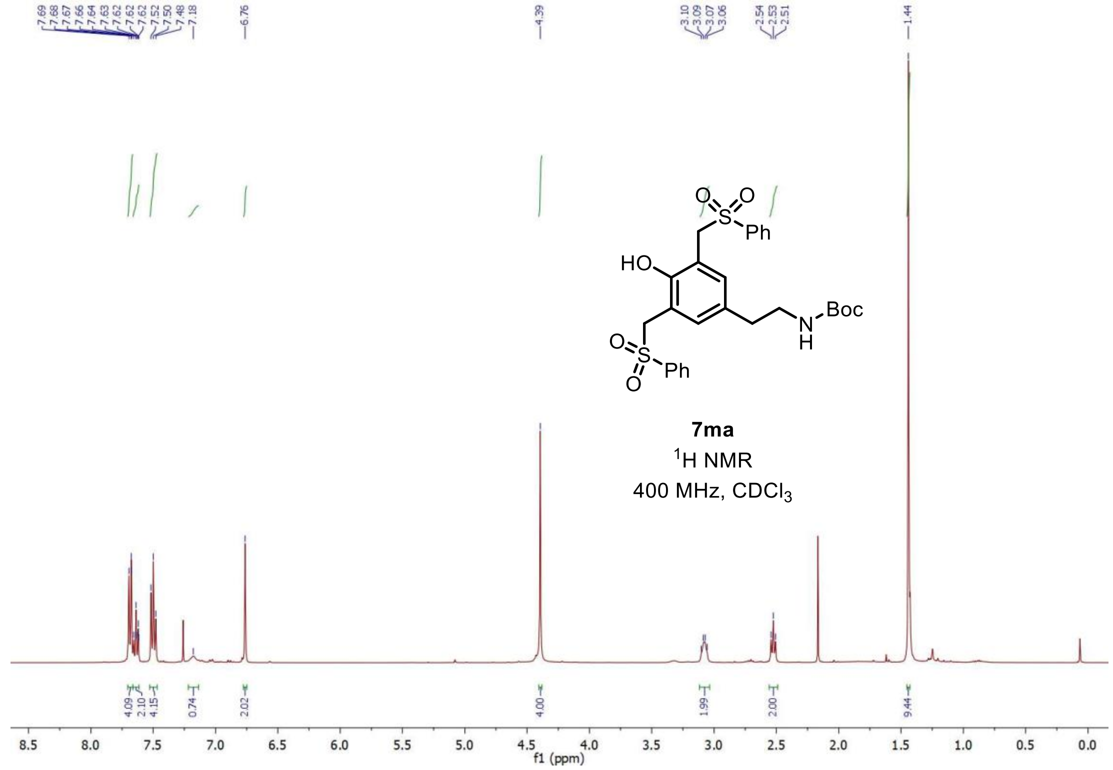
7.69
7.68
7.67
7.66
7.64
7.63
7.62
7.62
7.52
7.50
7.48
7.18
6.76
4.39
3.10
3.09
3.07
3.06
2.54
2.53
2.51
1.44
HO
S
O O
Ph
N
H
Boc
7ma
^{1}H NMR
400 MHz, $CDCl_3$
4.09
2.10
4.15
0.74
2.02
4.00
1.99
2.00
9.44
f1 (ppm)

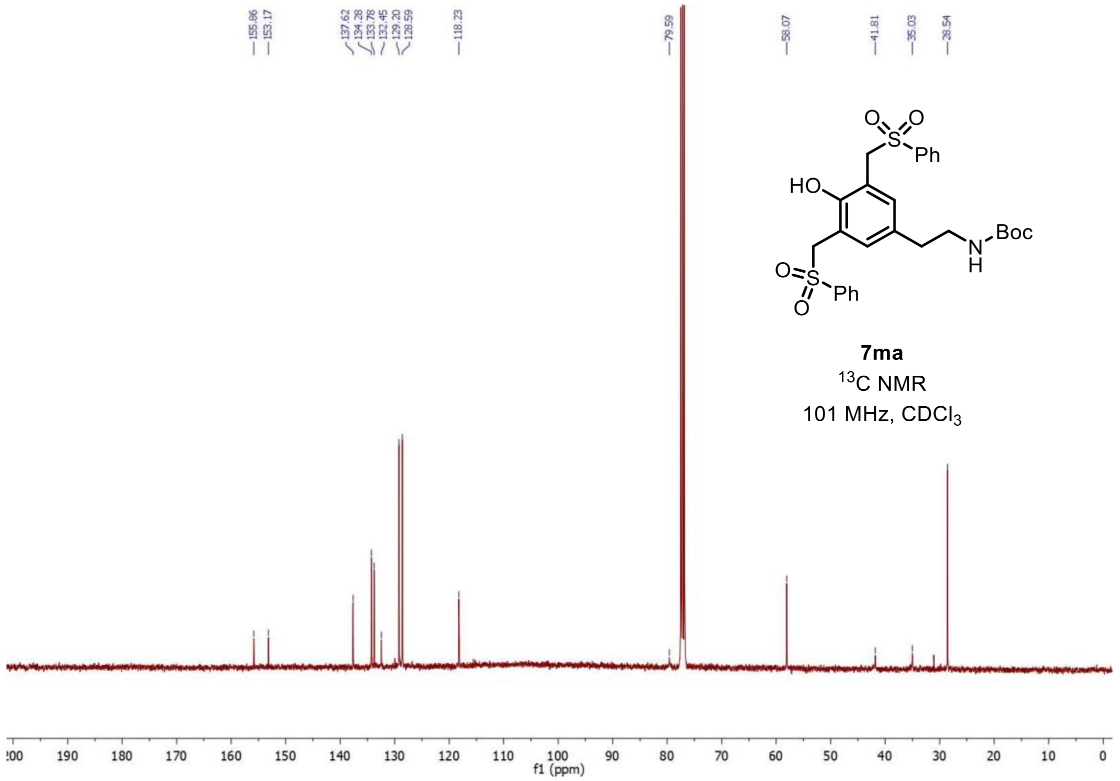
155.86
153.17
137.62
134.28
133.78
132.45
129.20
128.59
118.23
79.59
58.07
41.81
35.03
28.54
Ph
HO
N
H
Boc
Ph
7ma
^{13}C NMR
101 MHz, $CDCl_3$
f1 (ppm)

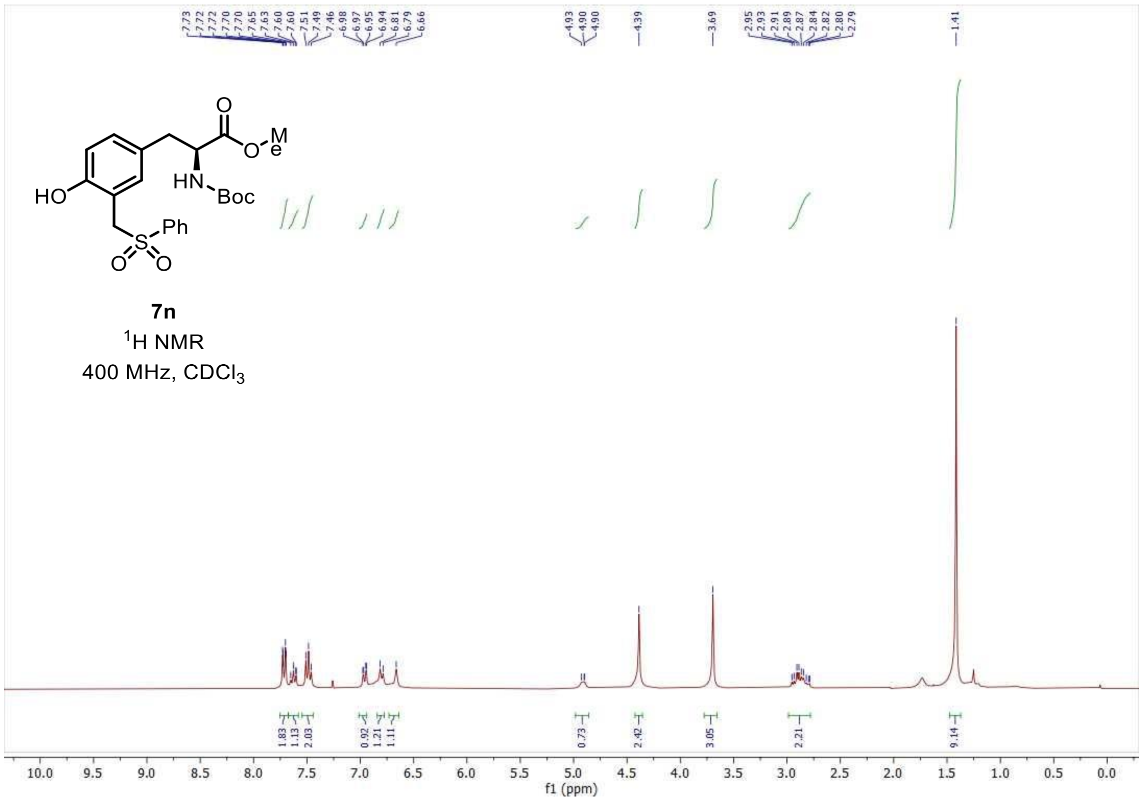
HO
HN
Boc
Ph
7n
^{1}H NMR
400 MHz, $CDCl_3$
f1 (ppm)

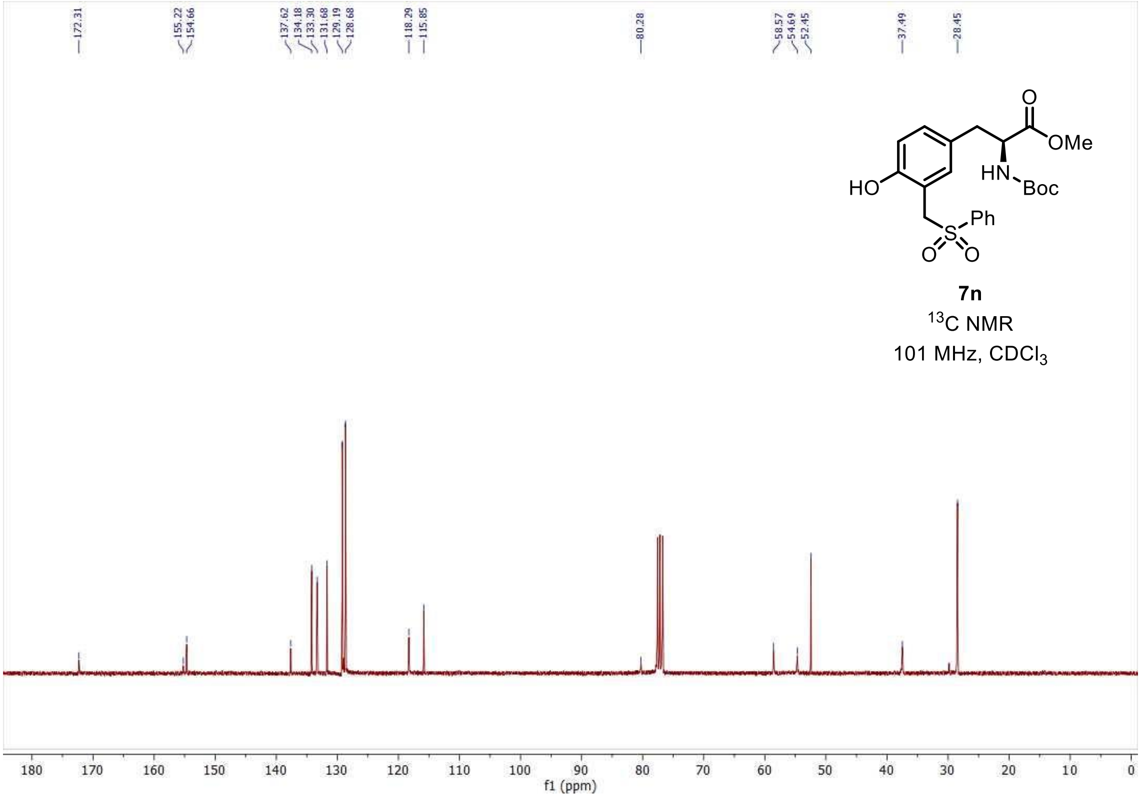

172.31
155.22
154.66
137.62
134.18
133.30
131.68
129.19
128.68
118.29
115.85
80.28
58.57
54.69
52.45
37.49
28.45
OMe
HN
Boc
HO
Ph
7n
13C NMR
101 MHz, CDCl3
f1 (ppm)

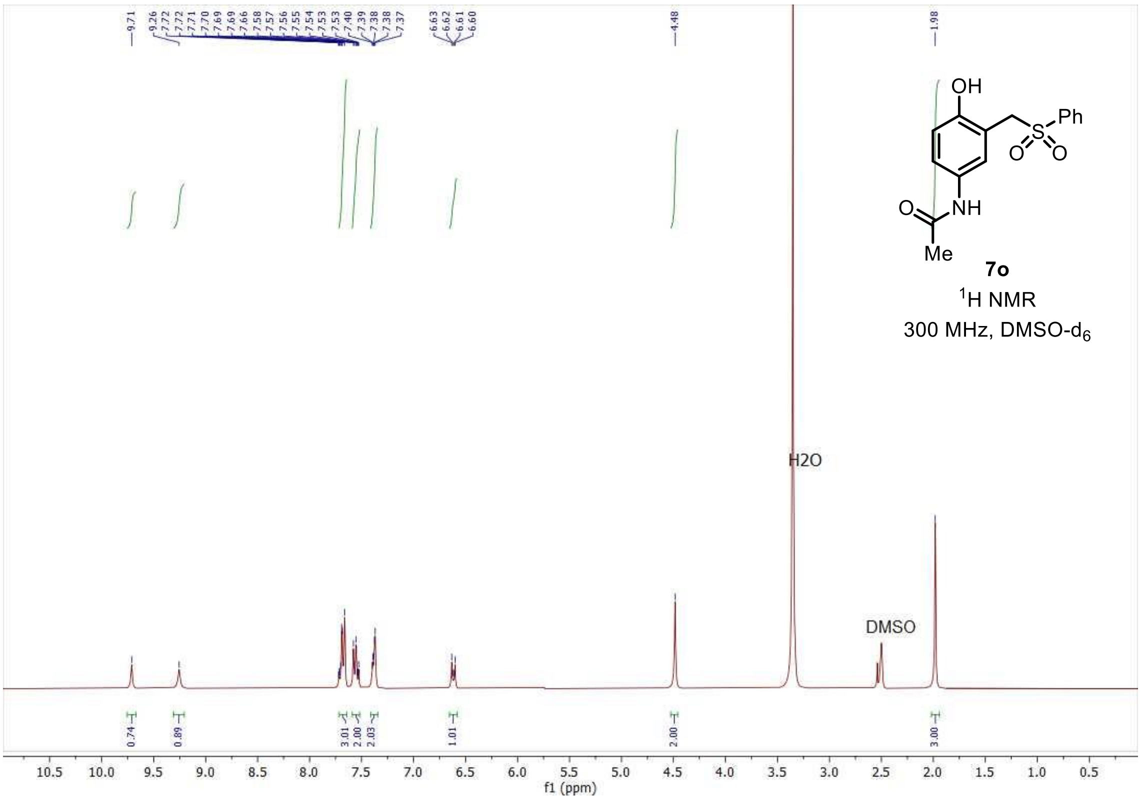

9.71
9.26
4.48
1.98
OH
Ph
NH
Me
7o
1H NMR
300 MHz, DMSO-d6
H2O
DMSO
0.74
0.89
3.01
2.00
2.03
1.01
2.00
3.00
f1 (ppm)

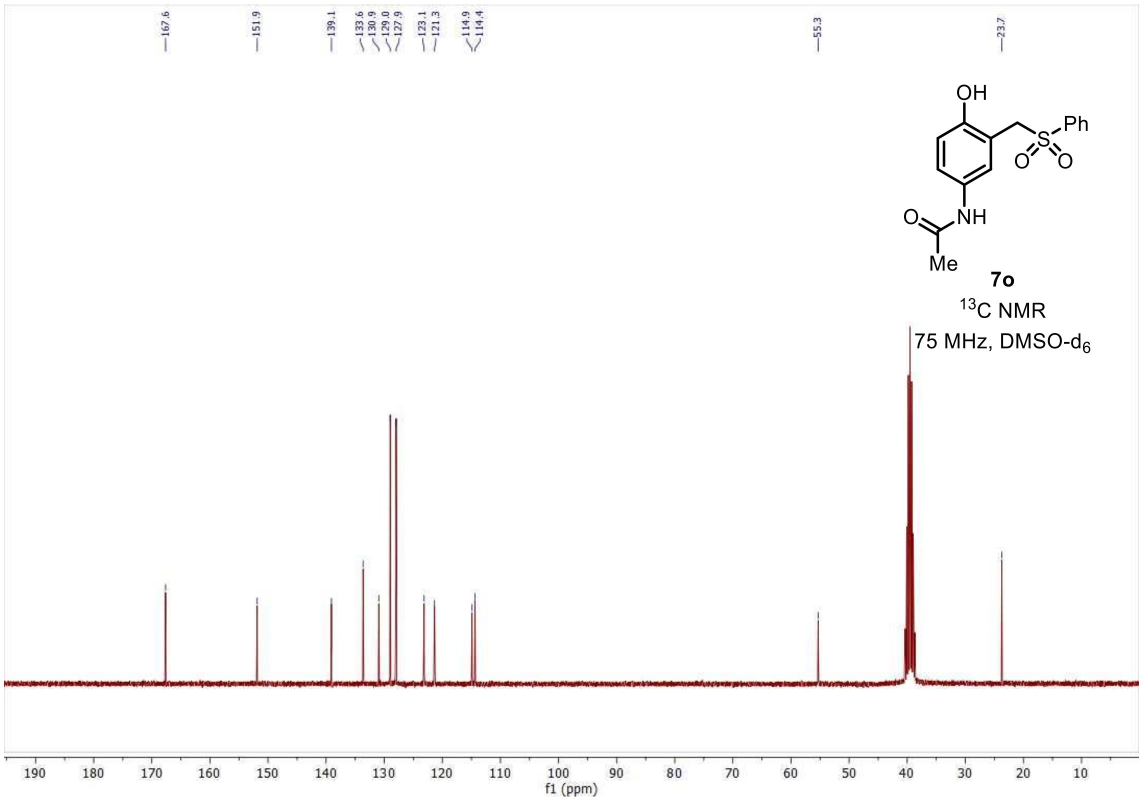
167.6
151.9
139.1
133.6
130.9
129.0
127.9
123.1
121.3
114.9
114.4
55.3
23.7
OH
Ph
S
O
O
NH
O
Me
7o
^{13}C NMR
75 MHz, DMSO-d_6
f1 (ppm)

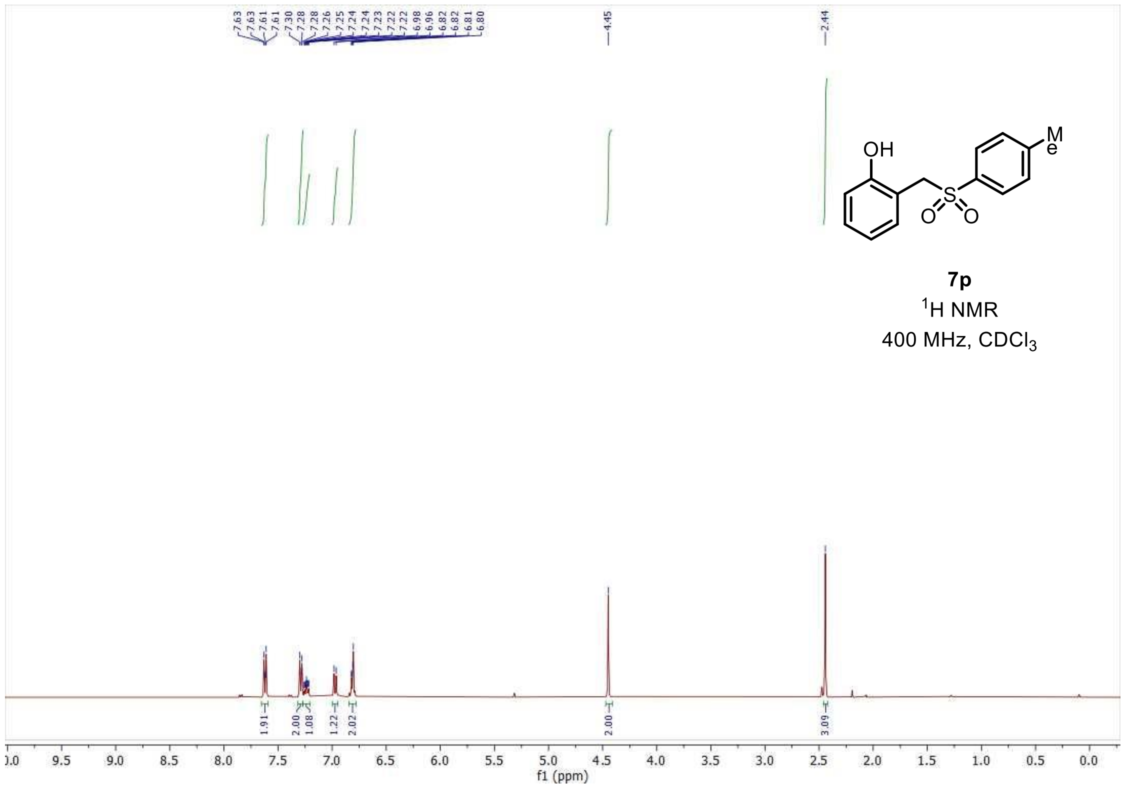
7.63
7.63
7.61
7.61
7.30
7.28
7.28
7.26
7.25
7.24
7.24
7.23
7.22
7.22
6.98
6.96
6.82
6.82
6.81
6.80
4.45
2.44
OH
S
O
O
Me
7p
^{1}H NMR
400 MHz, $CDCl_3$
1.91
2.00
1.08
1.22
2.02
2.00
3.09
f1 (ppm)

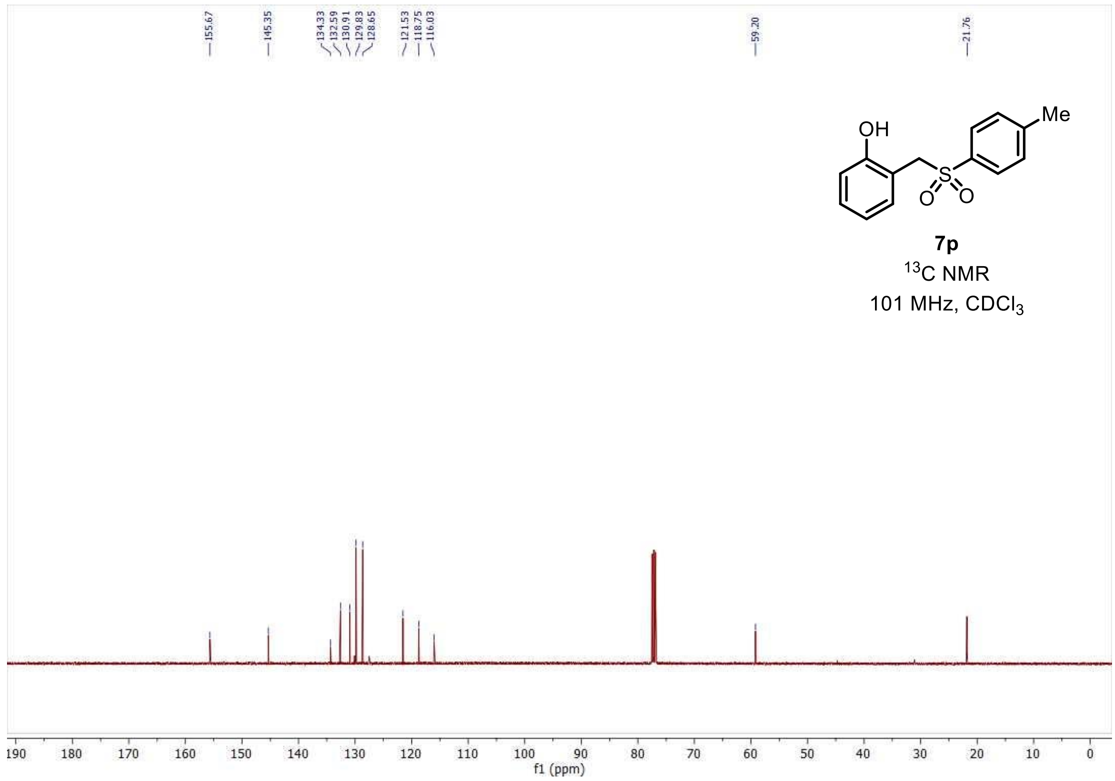
OH
Me
S
O
O
7p
13C NMR
101 MHz, CDCl3
f1 (ppm)

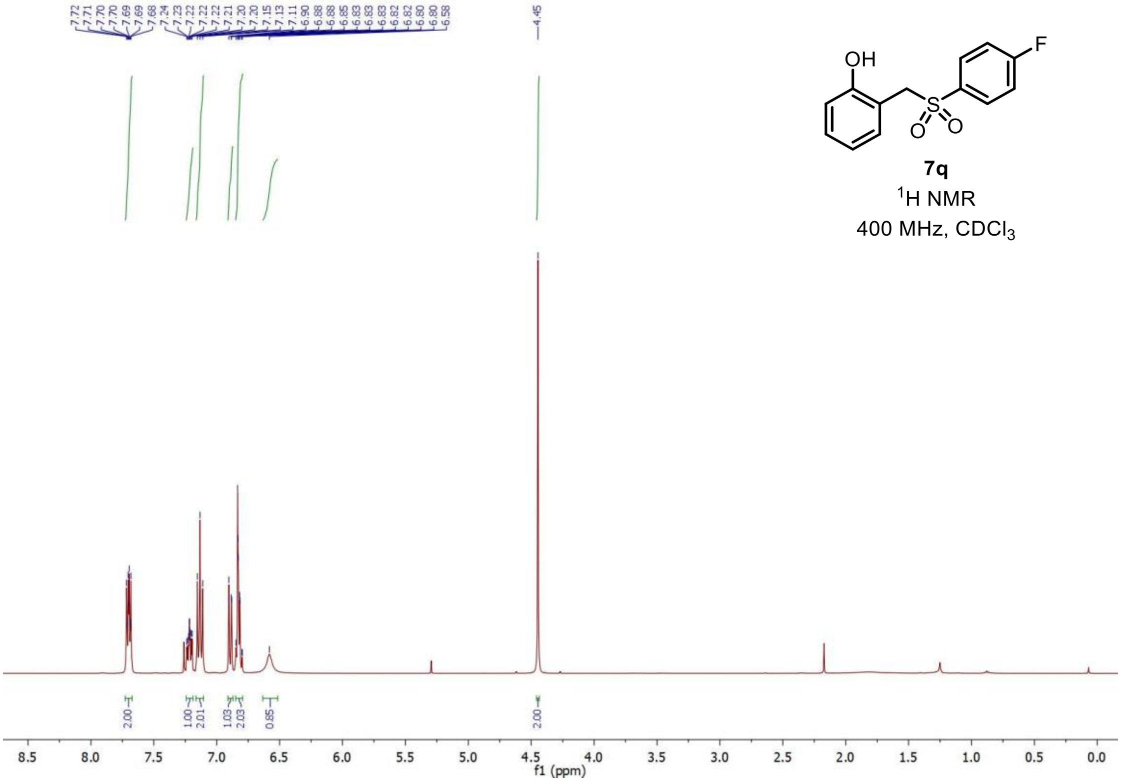
OH
F
S
O
O
7q
1H NMR
400 MHz, CDCl3
f1 (ppm)

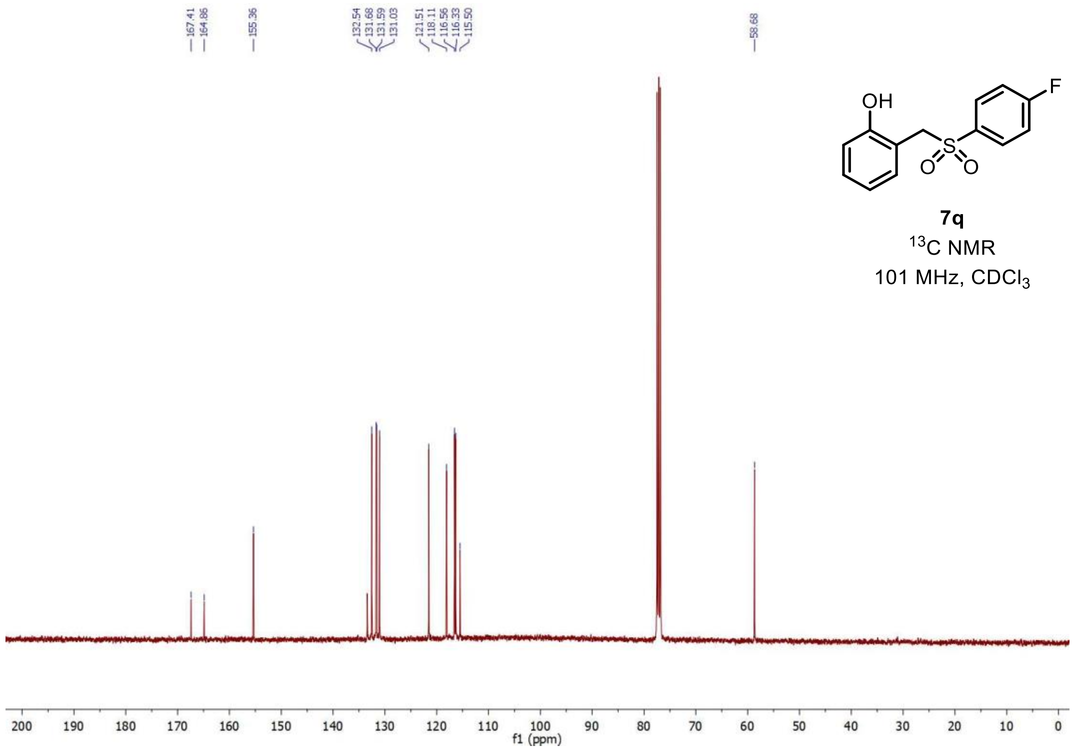
167.41
164.86
155.36
132.54
131.68
131.59
131.03
121.51
118.11
116.56
116.33
115.50
58.68
OH
F
S
O
O
7q
^{13}C NMR
101 MHz, $CDCl_3$
200 190 180 170 160 150 140 130 120 110 100 90 80 70 60 50 40 30 20 10 0
f1 (ppm)

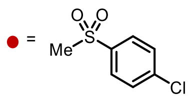
=
Me
S
O
O
Cl

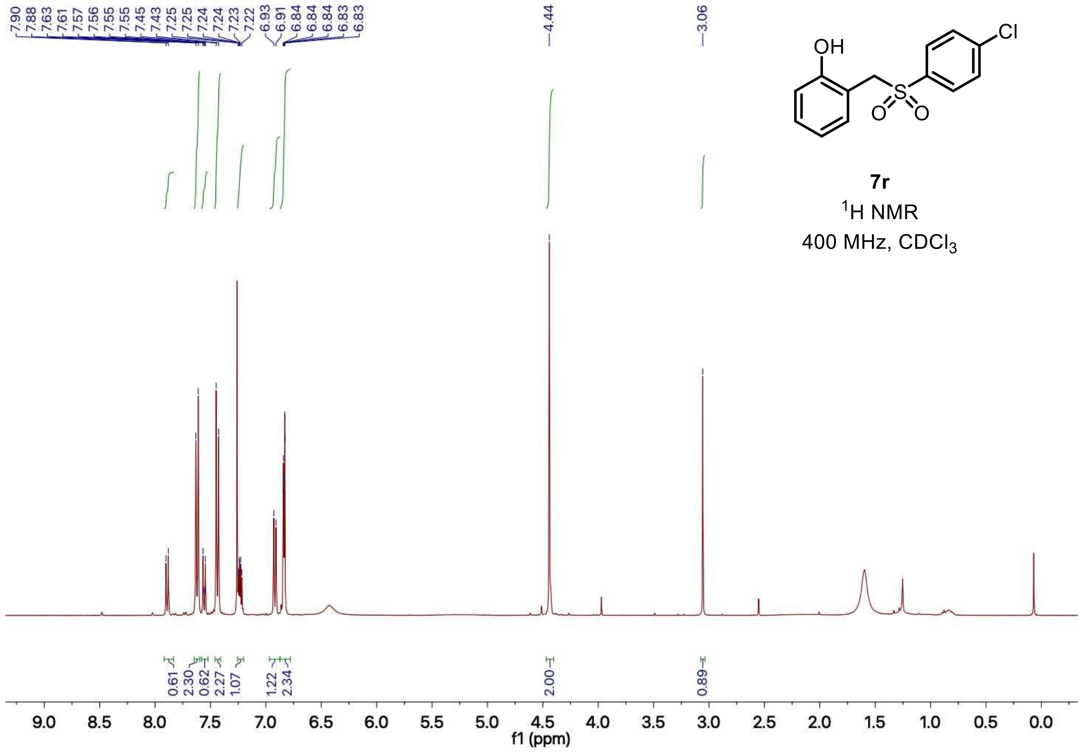

7r
^{1}H NMR
400 MHz, $CDCl_3$

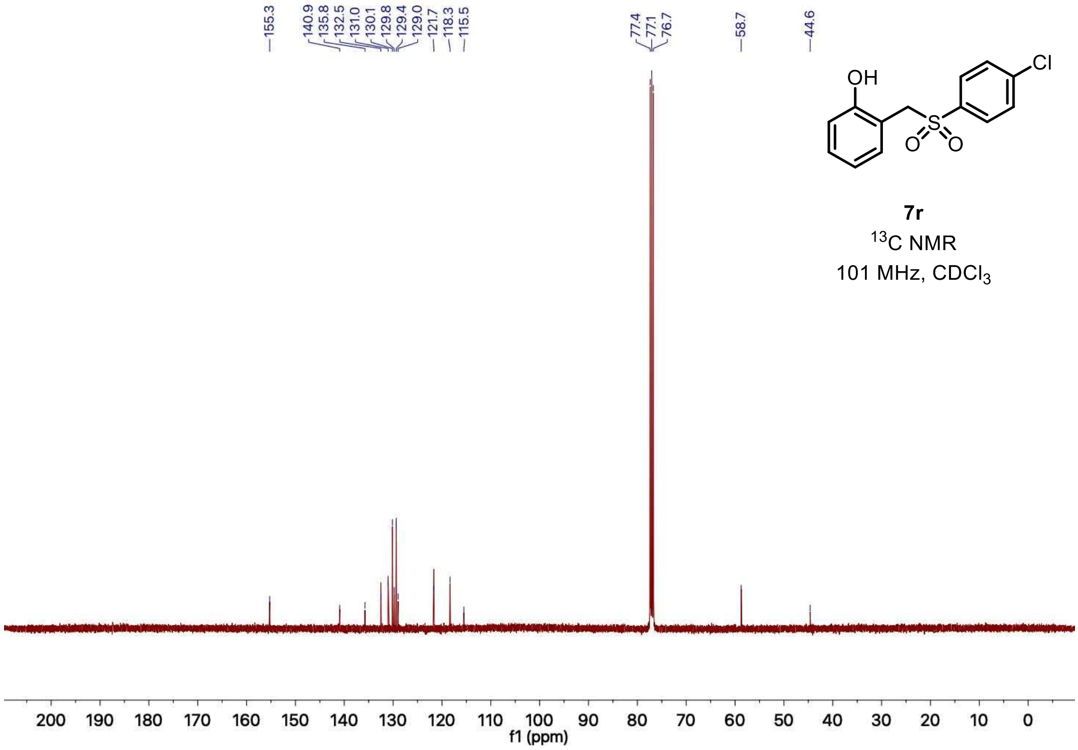

7r
^{13}C NMR
101 MHz, $CDCl_3$

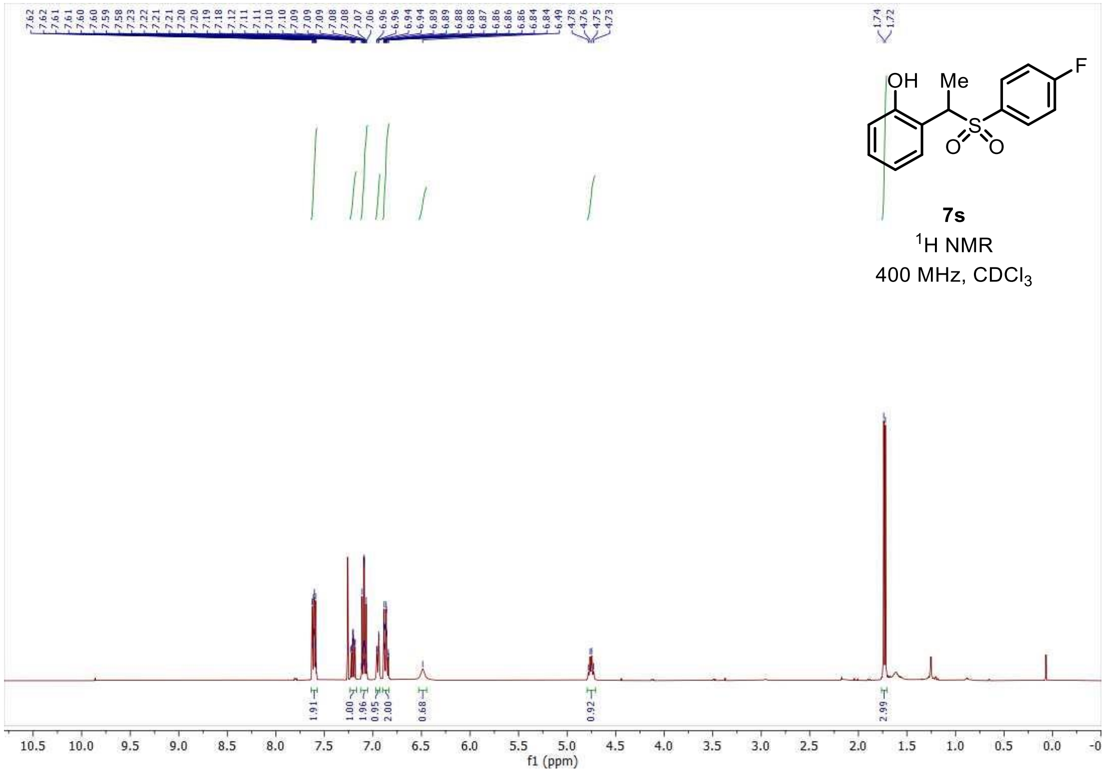
7s
1H NMR
400 MHz, CDCl3
f1 (ppm)

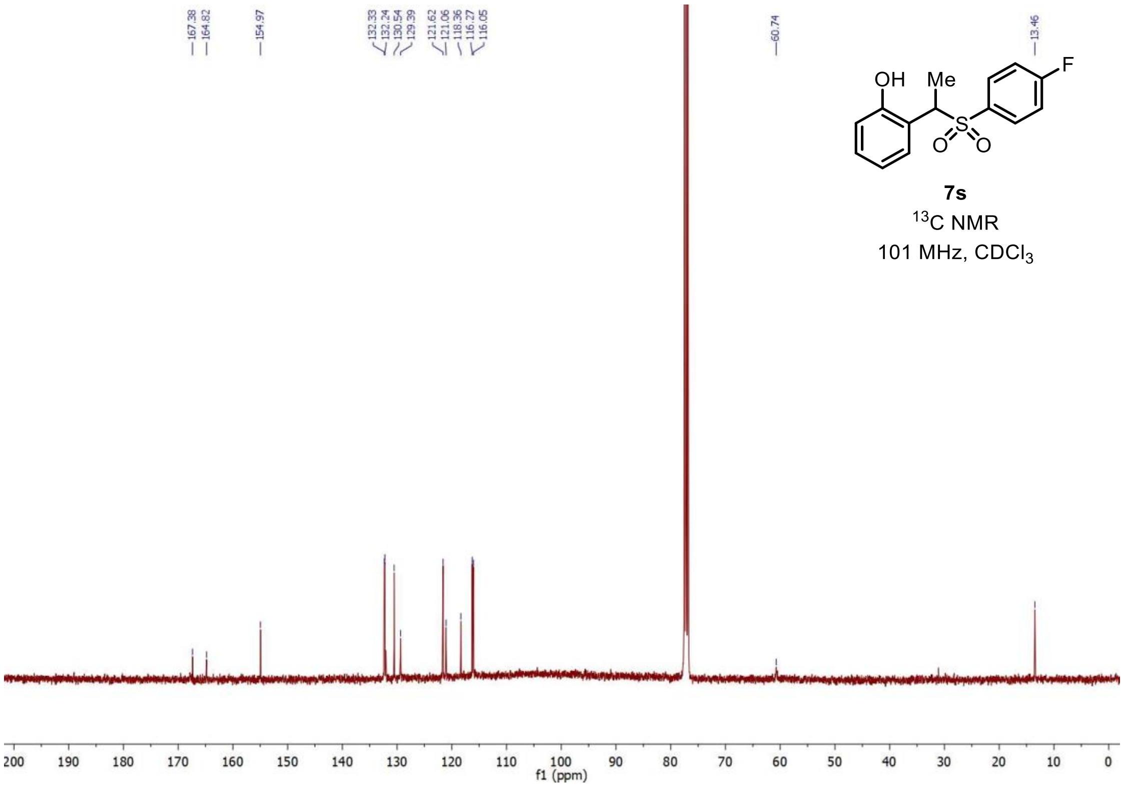
7s
13C NMR
101 MHz, CDCl3
f1 (ppm)

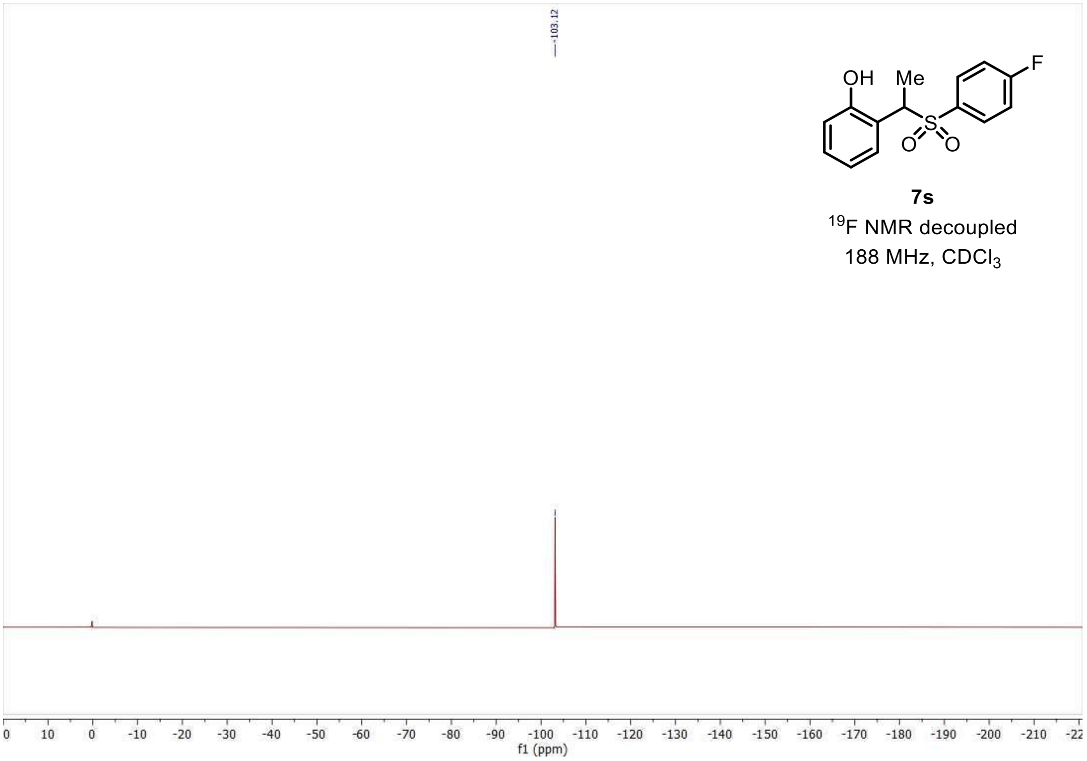
-103.12
OH
Me
F
S
O
O
7s
19F NMR decoupled
188 MHz, CDCl3
f1 (ppm)

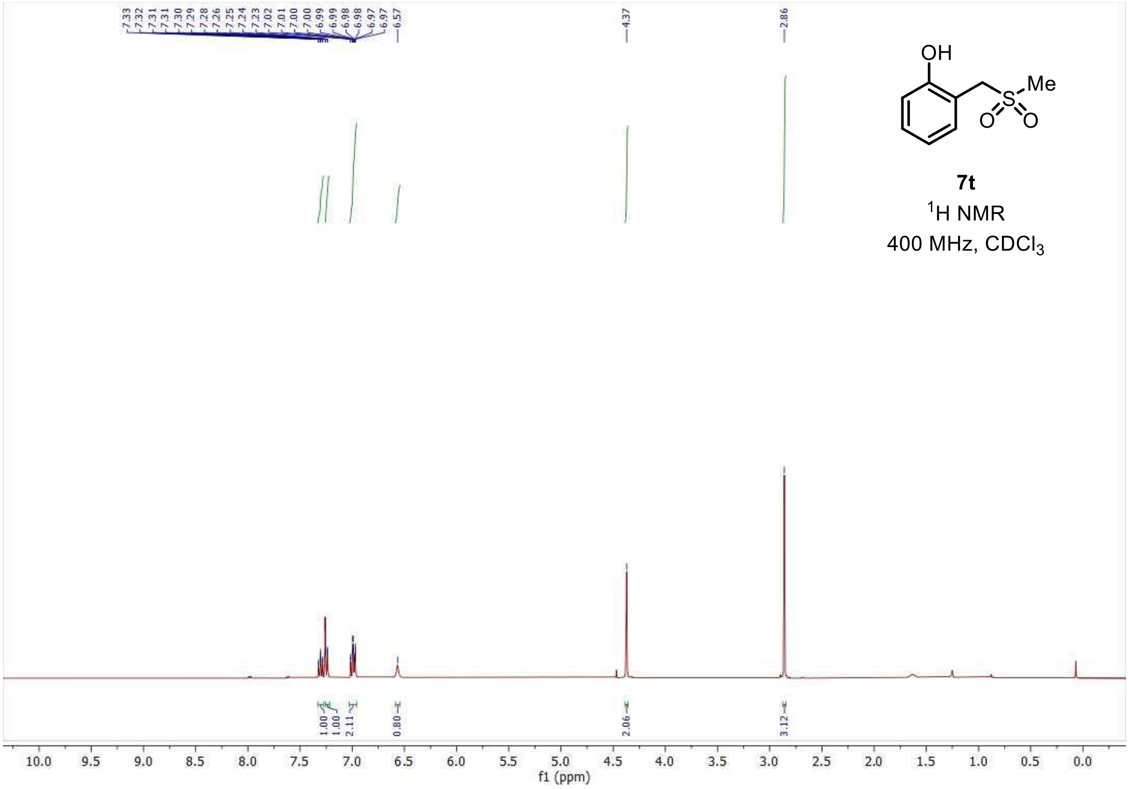
7.33
7.32
7.31
7.31
7.30
7.29
7.28
7.26
7.25
7.24
7.23
7.02
7.01
7.01
7.00
7.00
6.99
6.99
6.98
6.98
6.97
6.97
6.57
4.37
2.86
OH
S
Me
O
O
7t
1H NMR
400 MHz, CDCl3
1.00
1.00
2.11
0.80
2.06
3.12
f1 (ppm)

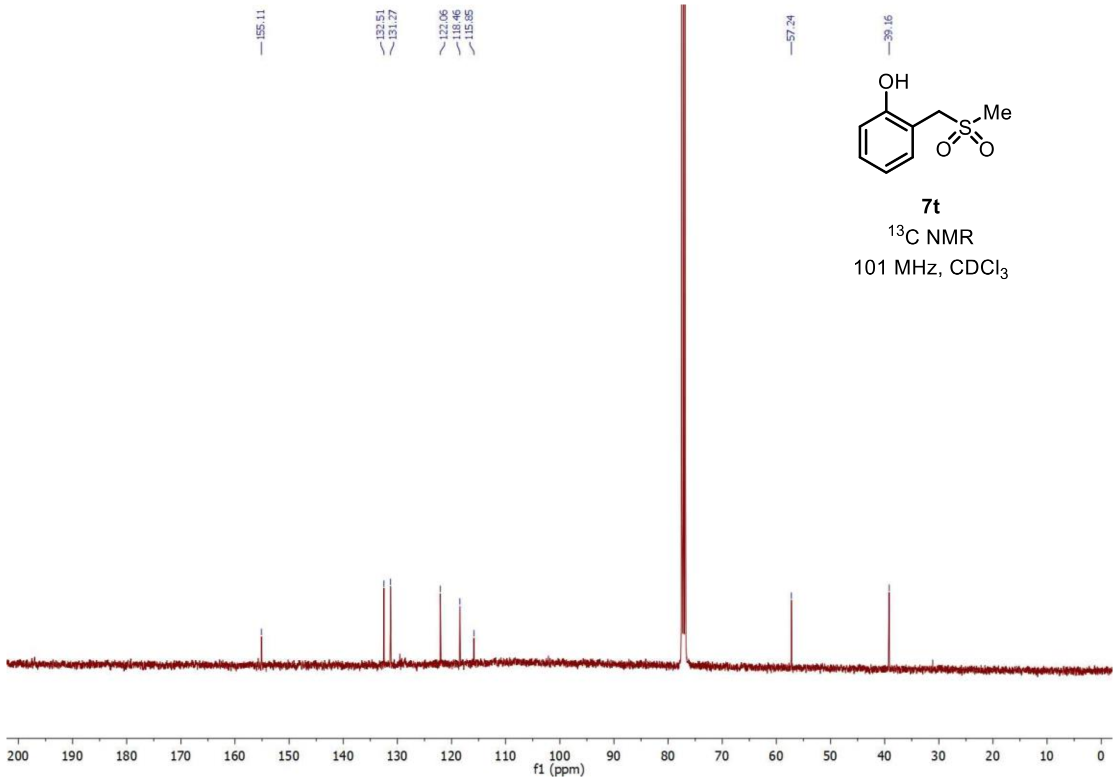
155.11
132.51
131.27
122.06
118.46
115.85
57.24
39.16
OH
S
Me
O
O
7t
13C NMR
101 MHz, CDCl3
f1 (ppm)

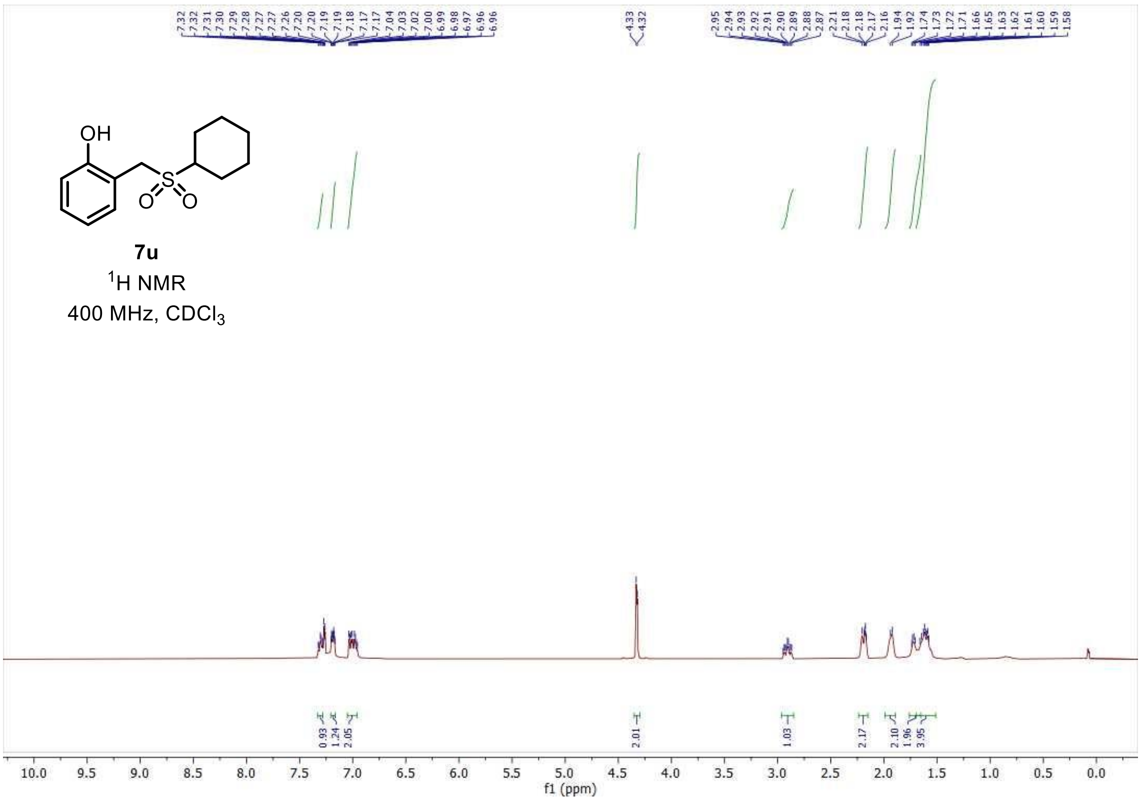
4.33
4.32
OH
S
O
O
7u
1H NMR
400 MHz, CDCl3
0.93
1.24
2.05
2.01
1.03
2.17
2.10
1.96
3.95
f1 (ppm)

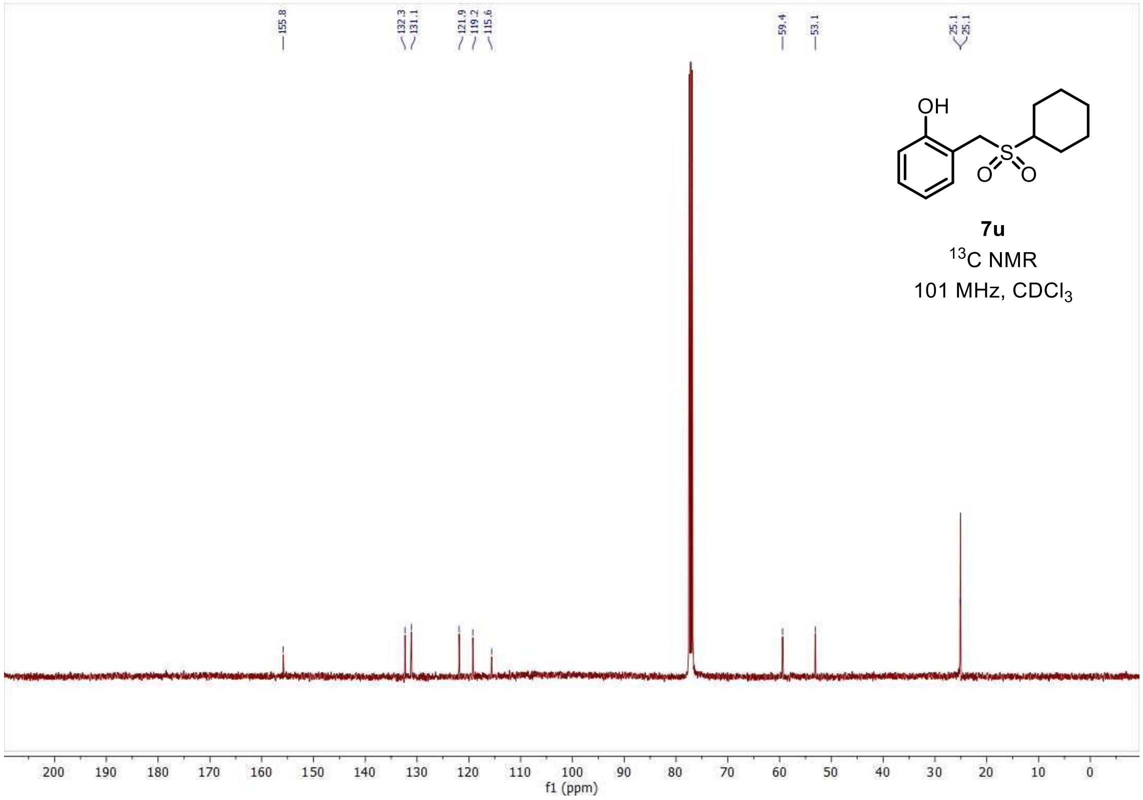
155.8
132.3
131.1
121.9
119.2
115.6
59.4
53.1
25.1
25.1
OH
S
O
O
7u
^{13}C NMR
101 MHz, $CDCl_3$
200 190 180 170 160 150 140 130 120 110 100 90 80 70 60 50 40 30 20 10 0
f1 (ppm)

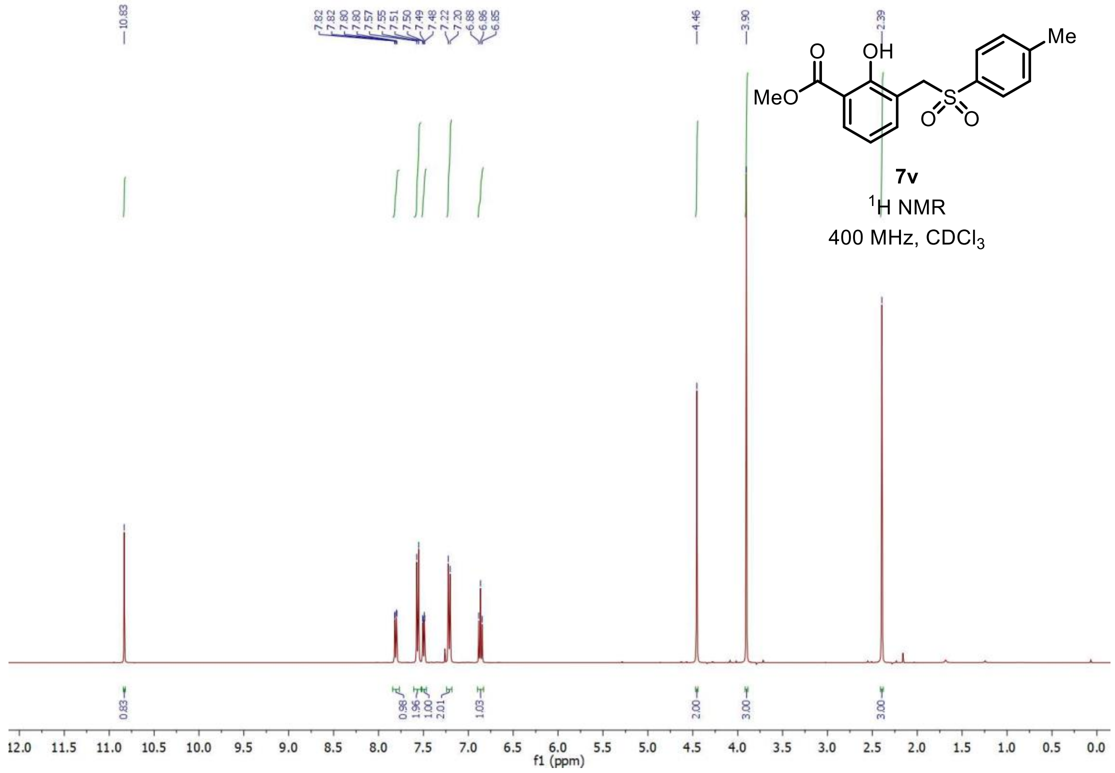
10.83
7.82
7.82
7.80
7.80
7.57
7.55
7.51
7.50
7.49
7.48
7.22
7.20
6.88
6.86
6.85
4.46
3.90
2.39
O
OH
Me
MeO
S
O
O
7v
^{1}H NMR
400 MHz, $CDCl_3$
0.83
0.98
1.96
1.00
2.01
1.03
2.00
3.00
3.00
12.0 11.5 11.0 10.5 10.0 9.5 9.0 8.5 8.0 7.5 7.0 6.5 6.0 5.5 5.0 4.5 4.0 3.5 3.0 2.5 2.0 1.5 1.0 0.5 0.0
f1 (ppm)

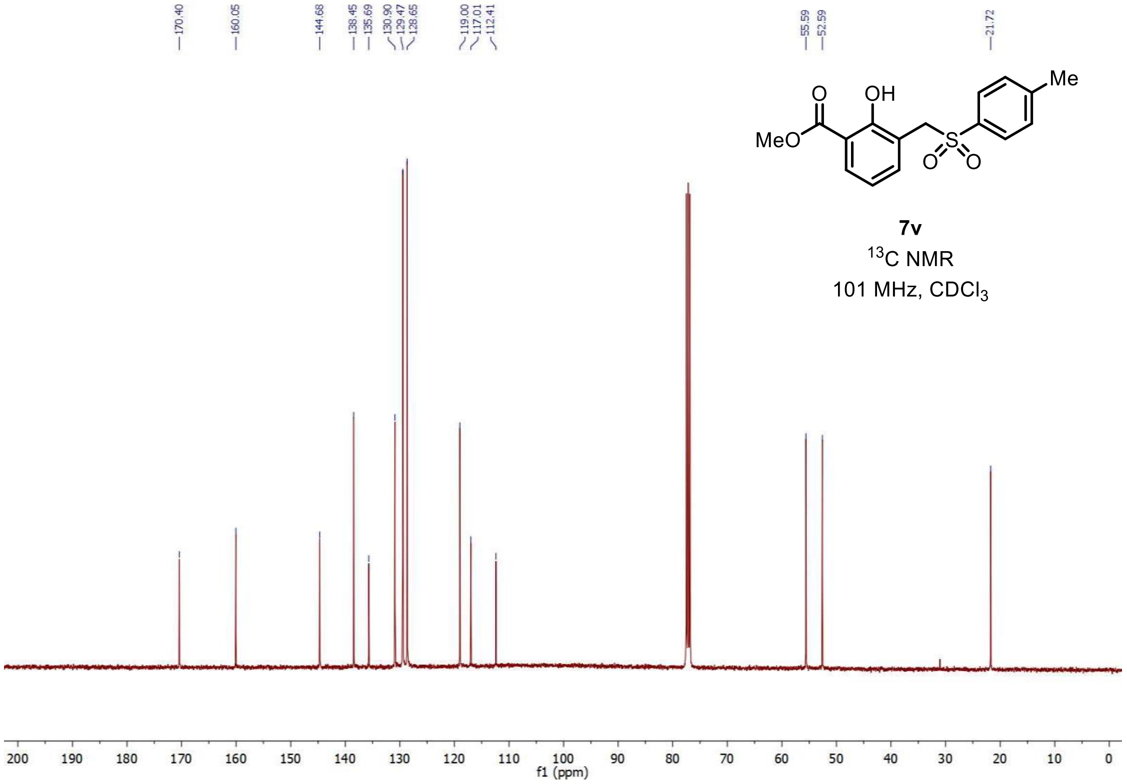

7v

^{13}C NMR

101 MHz, $CDCl_3$

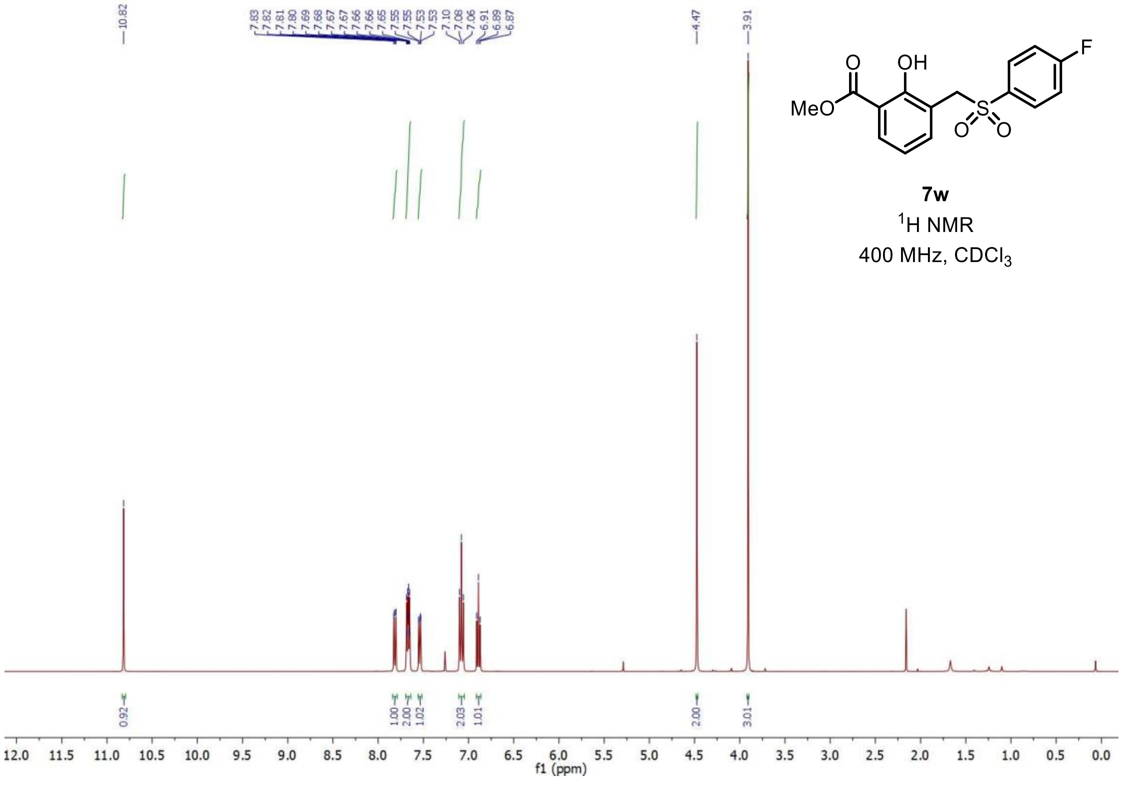

7w

^{1}H NMR

400 MHz, $CDCl_3$

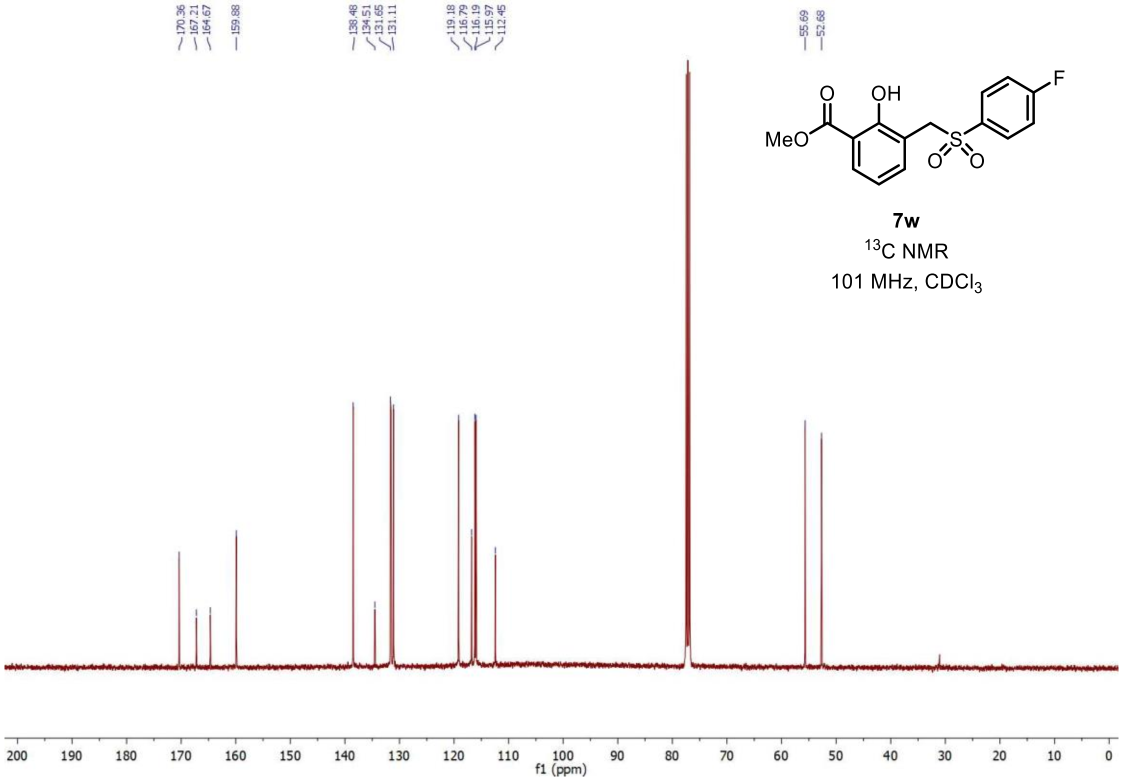
170.36
167.21
164.67
159.88
138.48
134.51
131.65
131.11
119.18
116.79
116.19
115.97
112.45
55.69
52.68
O
OH
F
MeO
S
O
O
7w
^{13}C NMR
101 MHz, $CDCl_3$
200 190 180 170 160 150 140 130 120 110 100 90 80 70 60 50 40 30 20 10 0
f1 (ppm)

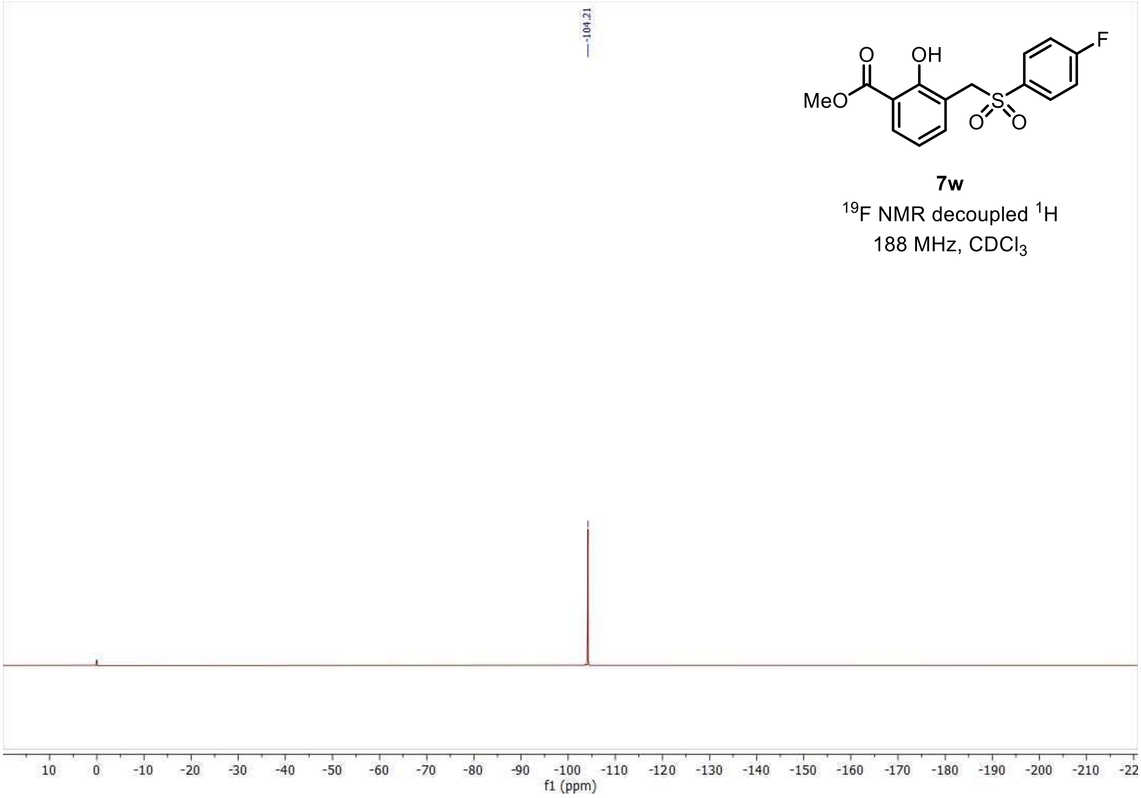
-104.21
O
OH
F
MeO
S
O
O
7w
^{19}F NMR decoupled ^{1}H
188 MHz, $CDCl_3$
10 0 -10 -20 -30 -40 -50 -60 -70 -80 -90 -100 -110 -120 -130 -140 -150 -160 -170 -180 -190 -200 -210 -22
f1 (ppm)

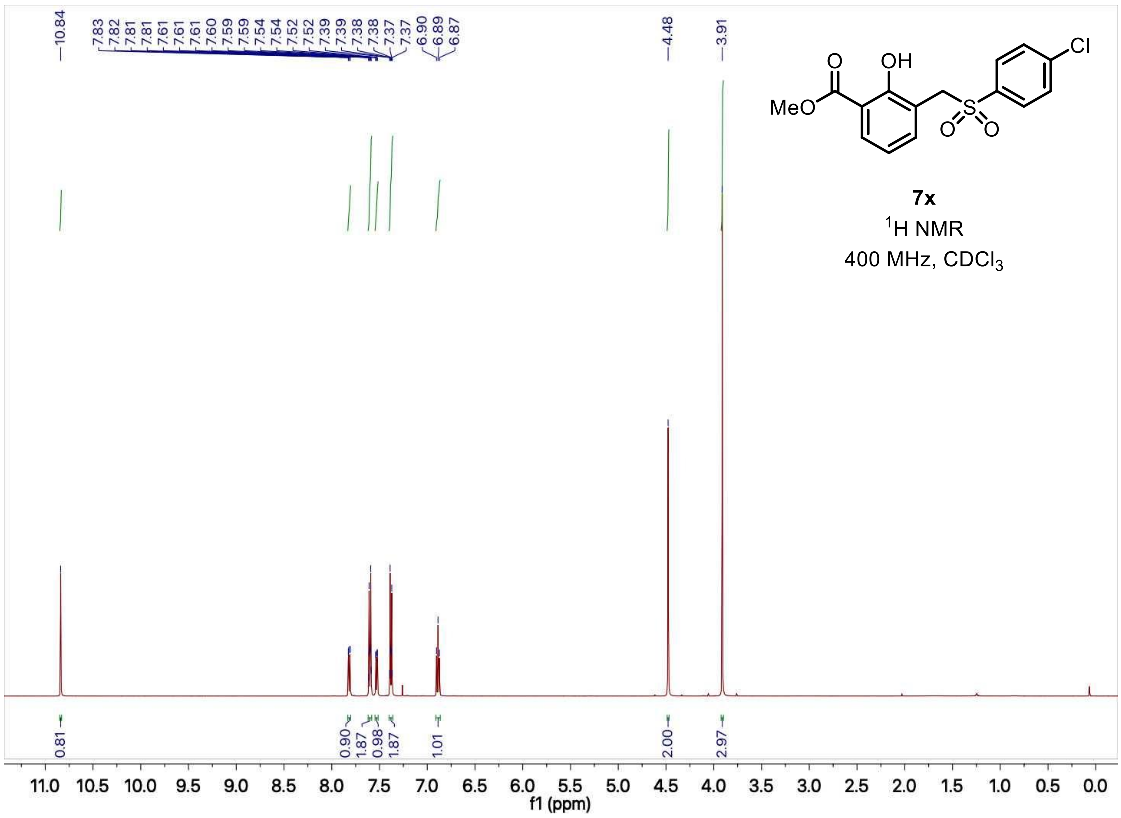

10.84
7.83
7.82
7.81
7.81
7.61
7.61
7.60
7.59
7.59
7.54
7.54
7.52
7.52
7.39
7.39
7.38
7.38
7.37
7.37
6.90
6.89
6.87
4.48
3.91
O
OH
Cl
MeO
S
O
O
7x
1H NMR
400 MHz, CDCl3
0.81
0.90
1.87
0.98
1.87
1.01
2.00
2.97
f1 (ppm)

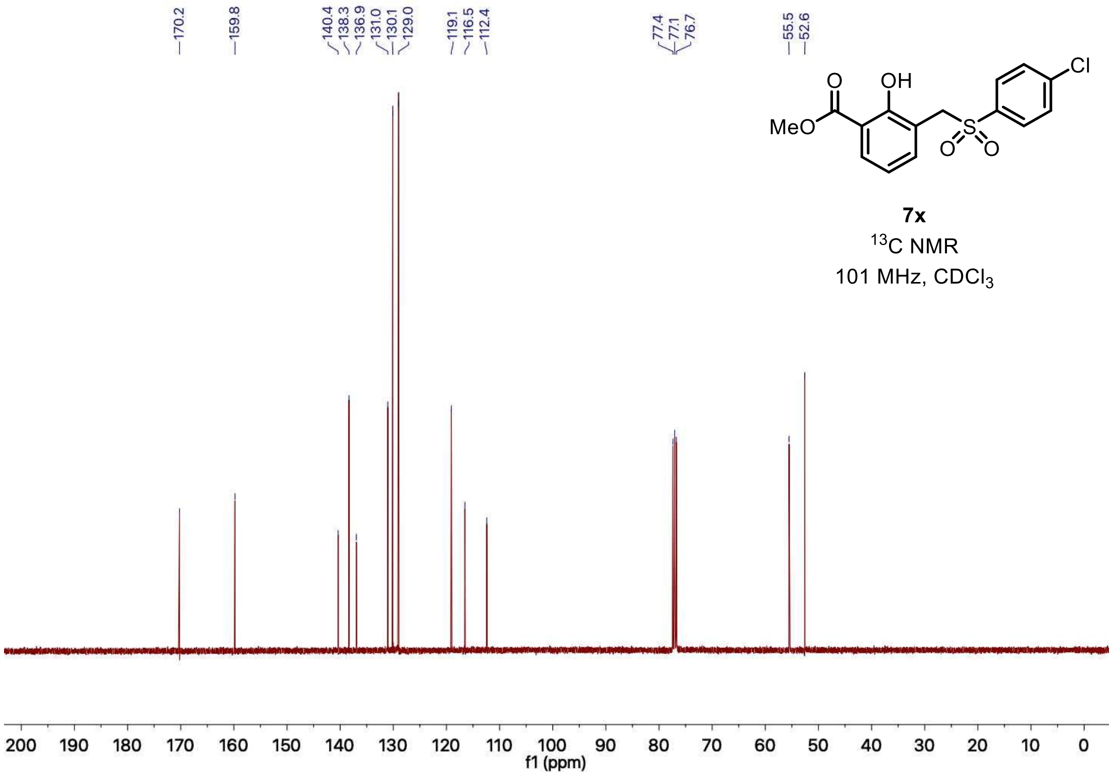

170.2
159.8
140.4
138.3
136.9
131.0
130.1
129.0
119.1
116.5
112.4
77.4
77.1
76.7
55.5
52.6
O
OH
Cl
MeO
S
O
O
7x
13C NMR
101 MHz, CDCl3
f1 (ppm)

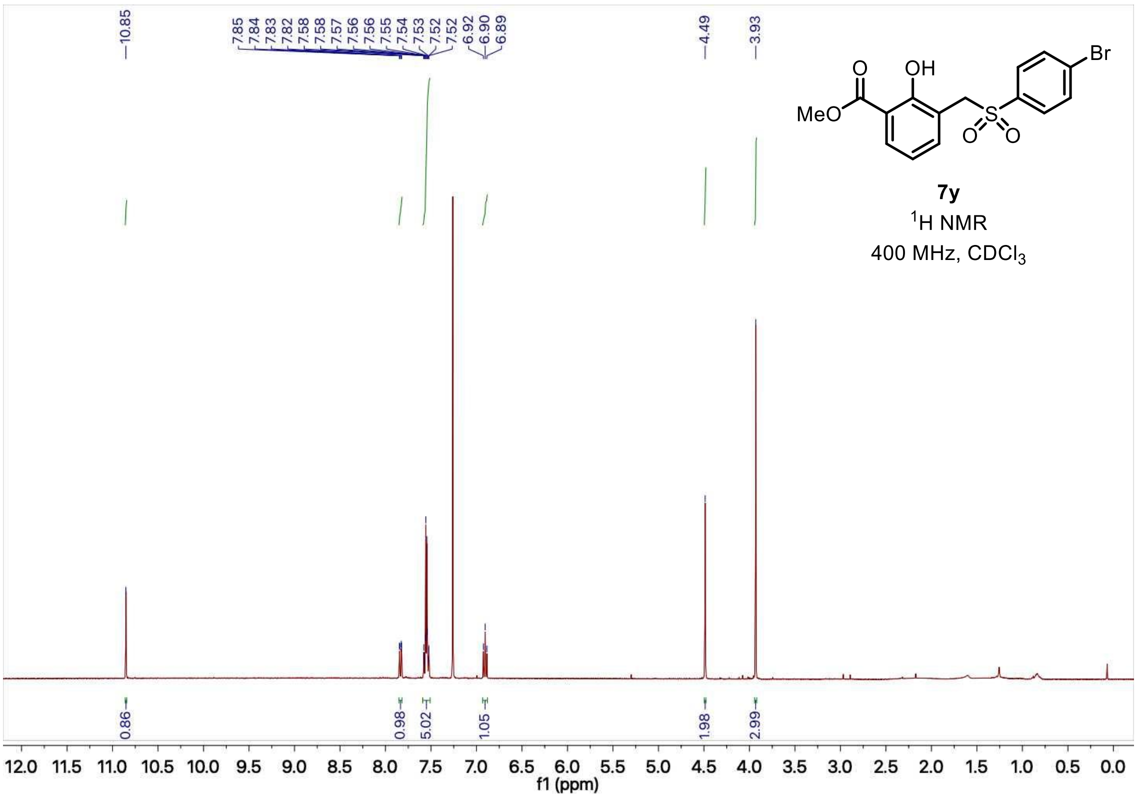
10.85
7.85
7.84
7.83
7.82
7.58
7.58
7.57
7.56
7.56
7.55
7.54
7.53
7.52
7.52
6.92
6.90
6.89
4.49
3.93
O
OH
MeO
S
O
O
Br
7y
^{1}H NMR
400 MHz, CDCl$_3$
0.86
0.98
5.02
1.05
1.98
2.99
12.0 11.5 11.0 10.5 10.0 9.5 9.0 8.5 8.0 7.5 7.0 6.5 6.0 5.5 5.0 4.5 4.0 3.5 3.0 2.5 2.0 1.5 1.0 0.5 0.0
f1 (ppm)

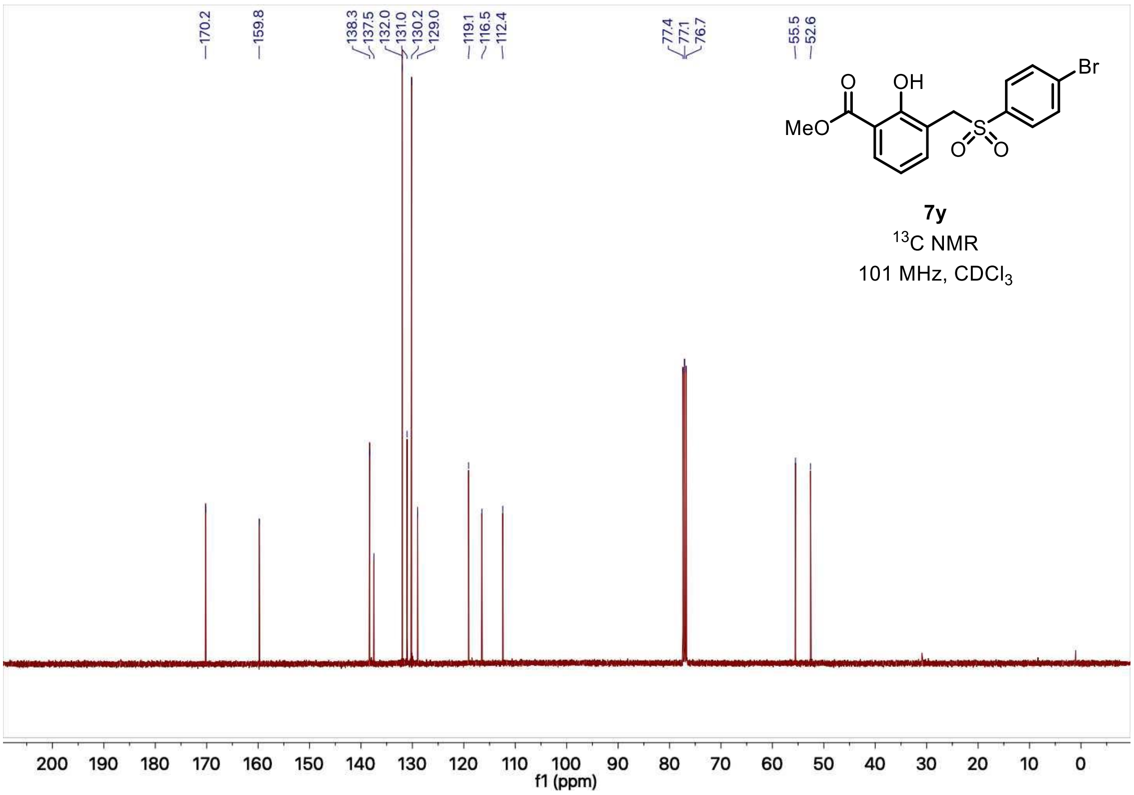
170.2
159.8
138.3
137.5
132.0
131.0
130.2
129.0
119.1
116.5
112.4
77.4
77.1
76.7
55.5
52.6
O
OH
MeO
S
O
O
Br
7y
^{13}C NMR
101 MHz, CDCl$_3$
200 190 180 170 160 150 140 130 120 110 100 90 80 70 60 50 40 30 20 10 0
f1 (ppm)

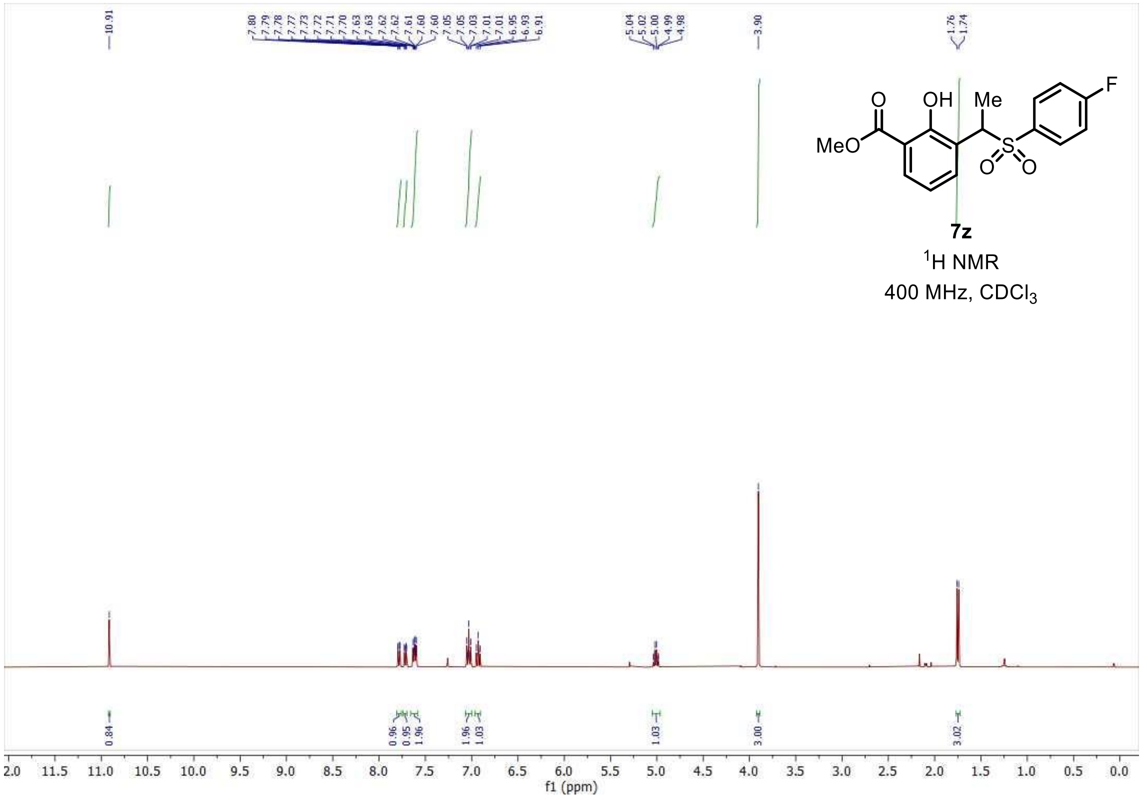
MeO
O
OH
Me
F
O
S
O
7z
1H NMR
400 MHz, CDCl3
f1 (ppm)

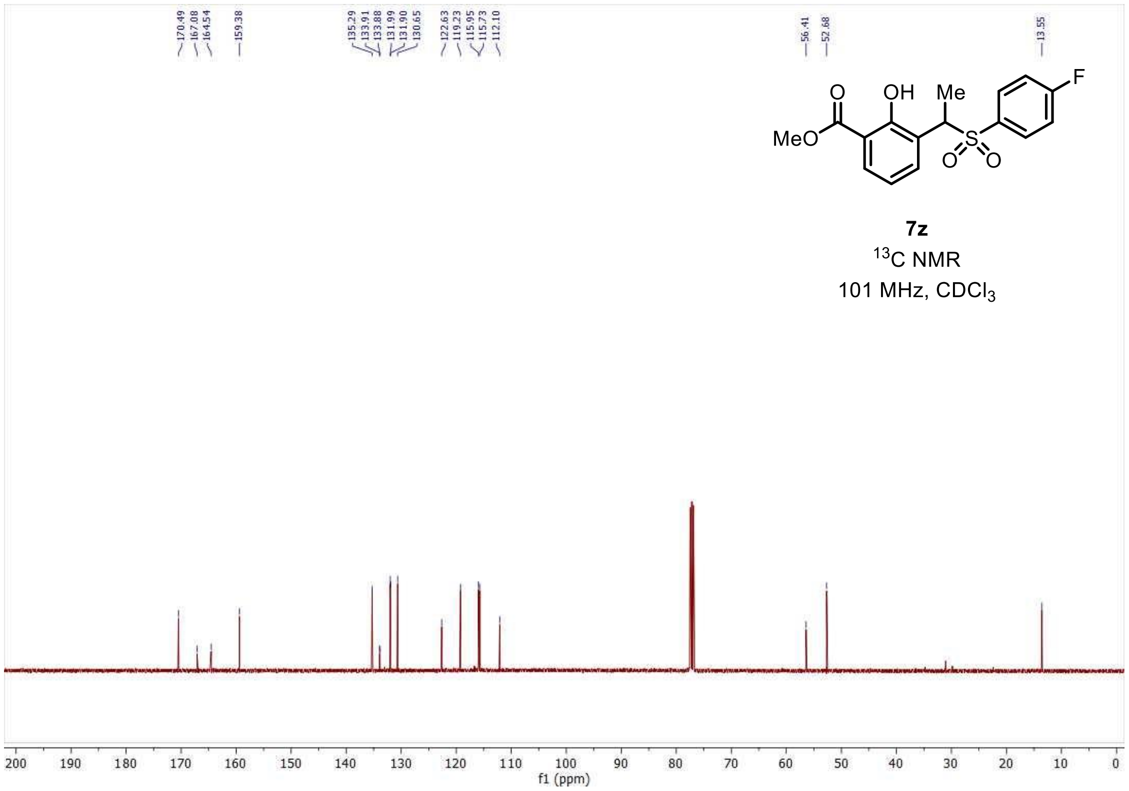
MeO
O
OH
Me
F
O
S
O
7z
13C NMR
101 MHz, CDCl3
f1 (ppm)

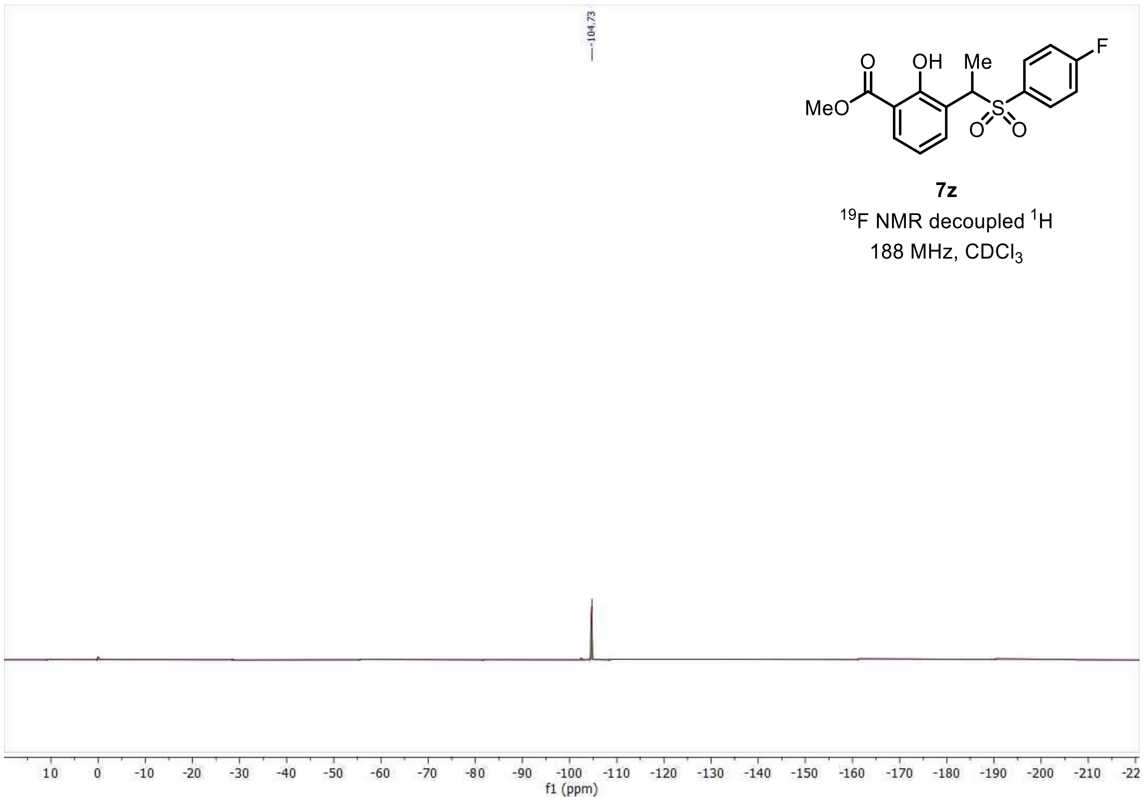
-104.73
7z
^{19}F NMR decoupled ^{1}H
188 MHz, $CDCl_3$
f1 (ppm)

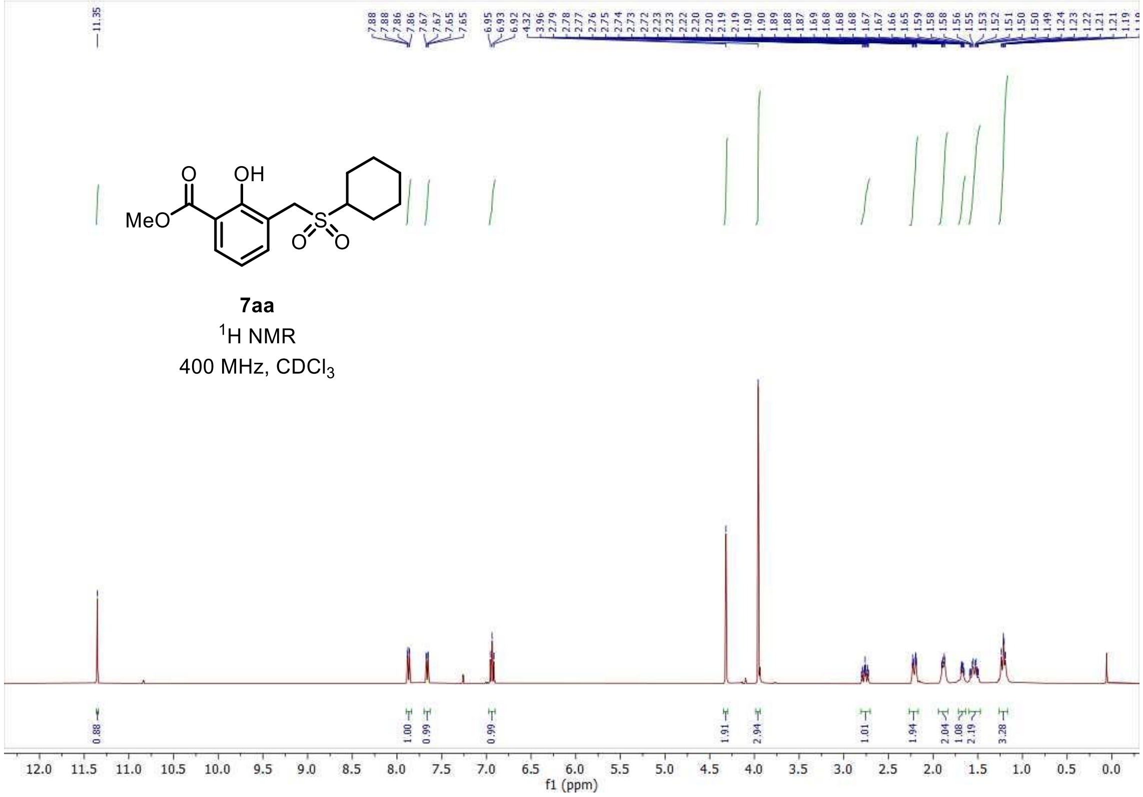
11.35
7aa
^{1}H NMR
400 MHz, $CDCl_3$
f1 (ppm)

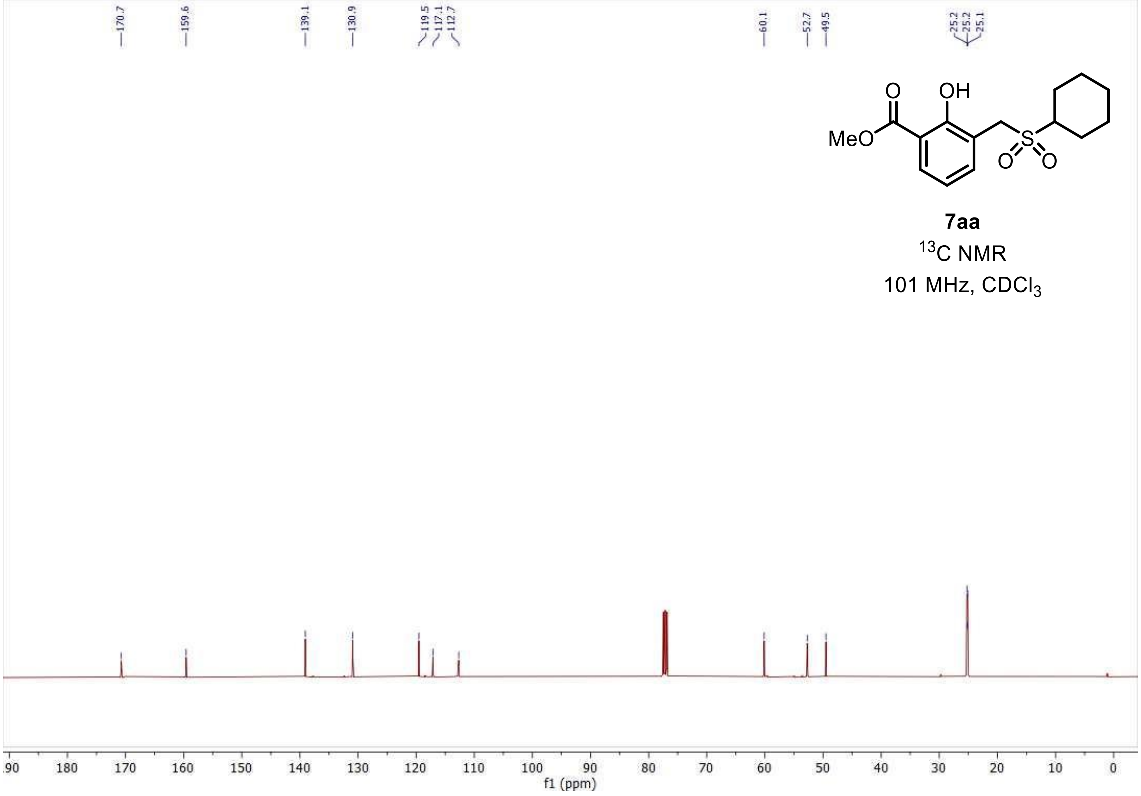
170.7
159.6
139.1
130.9
119.5
117.1
112.7
60.1
52.7
49.5
25.2
25.2
25.1
O
OH
MeO
S
O
O
7aa
^{13}C NMR
101 MHz, $CDCl_3$
f1 (ppm)

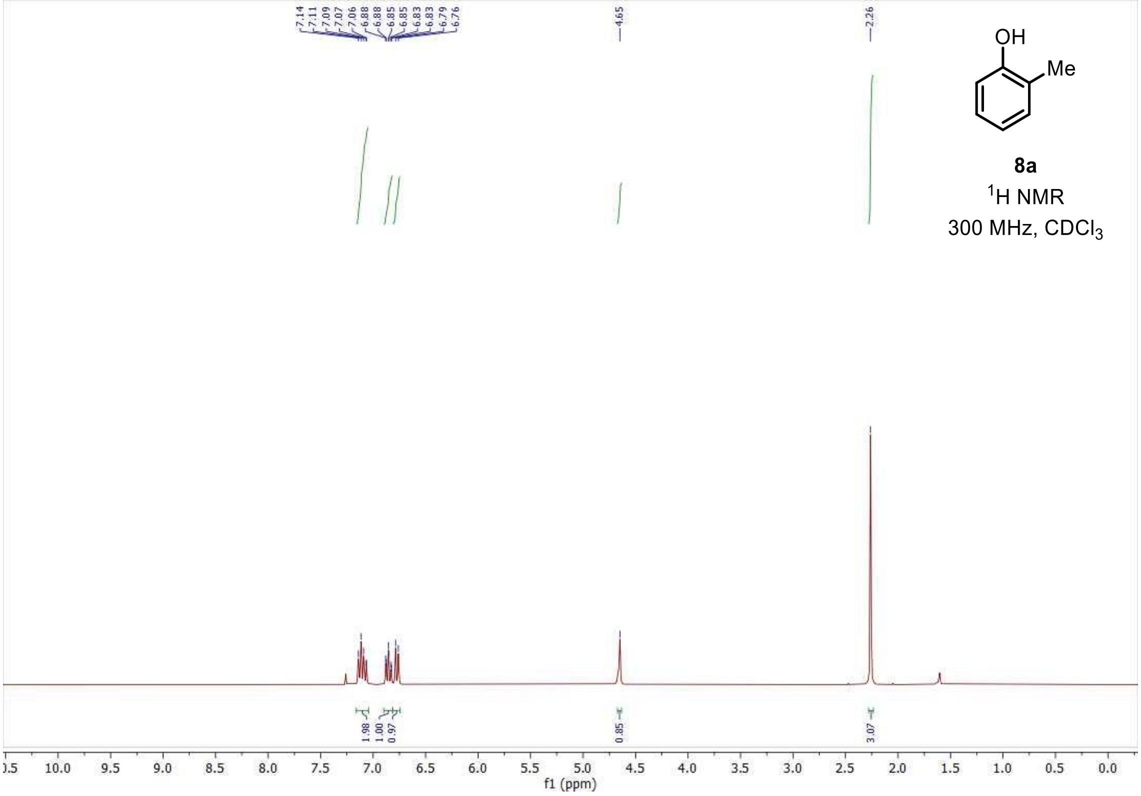
7.14
7.11
7.09
7.07
7.06
6.88
6.85
6.85
6.83
6.83
6.79
6.76
4.65
2.26
OH
Me
8a
^{1}H NMR
300 MHz, $CDCl_3$
1.98
1.00
0.97
0.85
3.07
f1 (ppm)

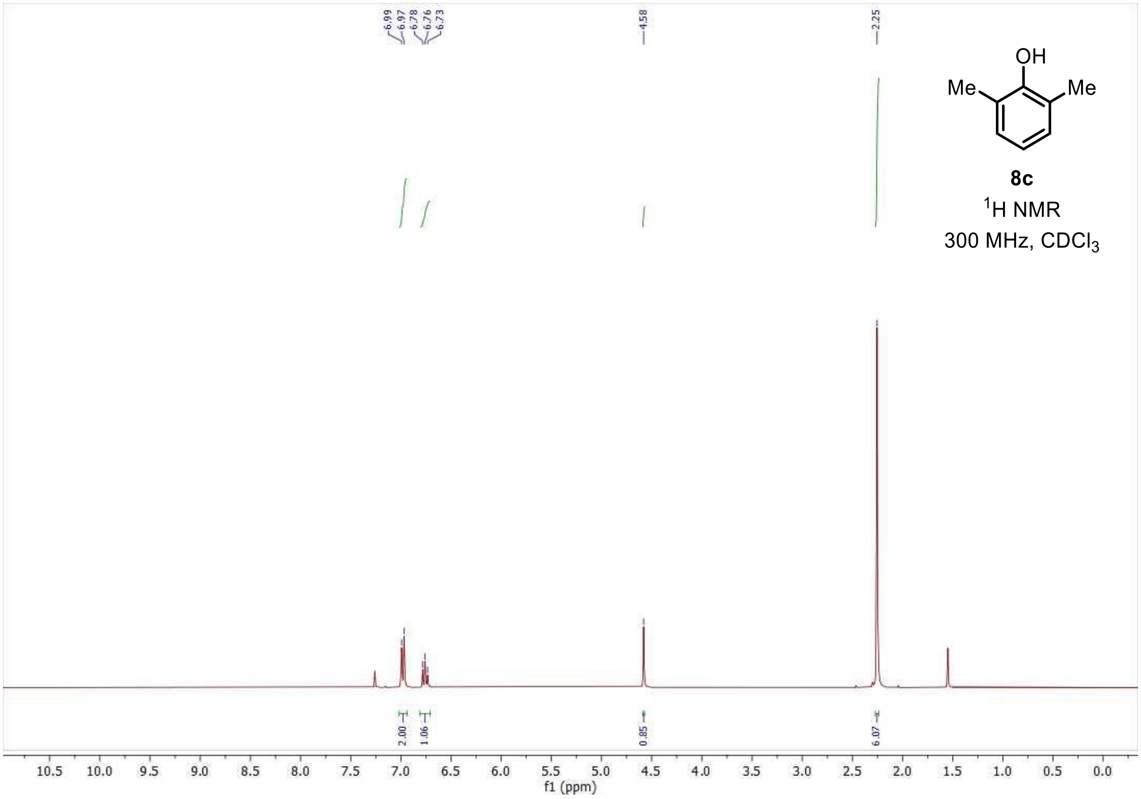
6.99
6.97
6.78
6.76
6.73
4.58
2.25
OH
Me
Me
8c
^{1}H NMR
300 MHz, $CDCl_3$
2.00
1.06
0.85
6.07
10.5 10.0 9.5 9.0 8.5 8.0 7.5 7.0 6.5 6.0 5.5 5.0 4.5 4.0 3.5 3.0 2.5 2.0 1.5 1.0 0.5 0.0
f1 (ppm)

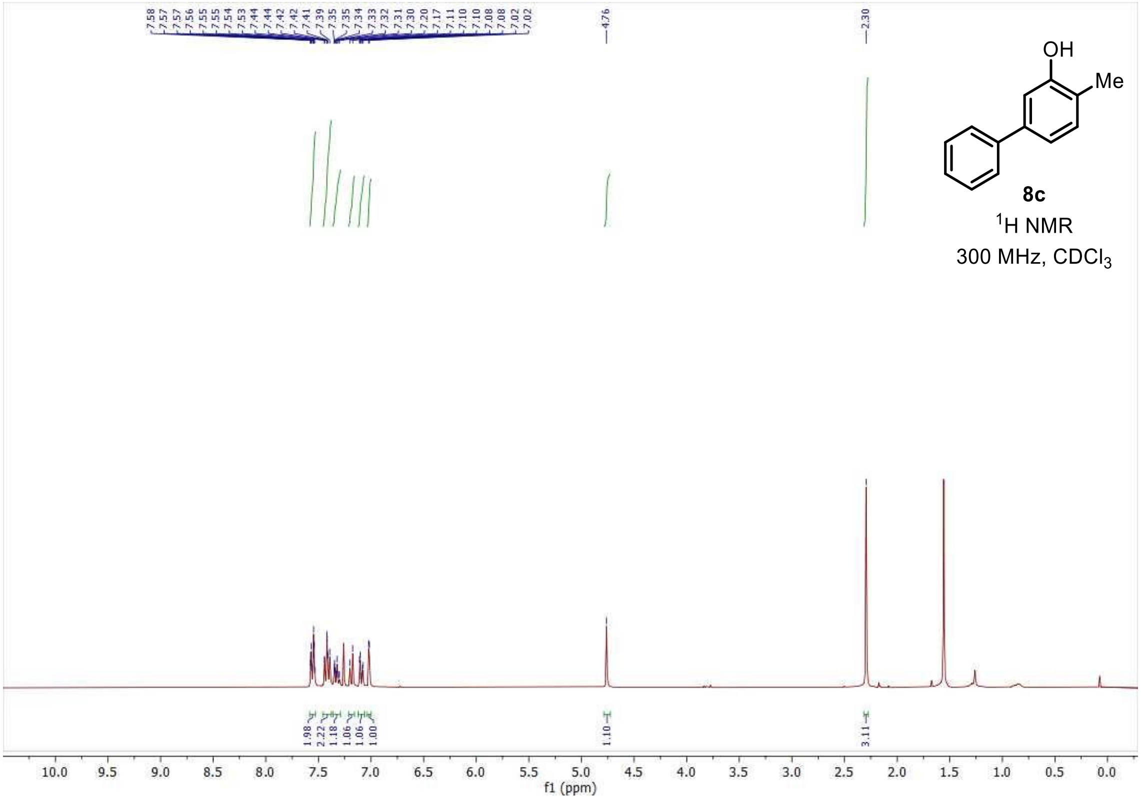
7.58
7.57
7.56
7.55
7.55
7.54
7.53
7.44
7.42
7.42
7.41
7.39
7.35
7.35
7.34
7.33
7.32
7.31
7.30
7.20
7.17
7.11
7.10
7.10
7.08
7.02
7.02
4.76
2.30
OH
Me
8c
^{1}H NMR
300 MHz, $CDCl_3$
1.98
2.22
1.18
1.06
1.06
1.00
1.10
3.11
10.0 9.5 9.0 8.5 8.0 7.5 7.0 6.5 6.0 5.5 5.0 4.5 4.0 3.5 3.0 2.5 2.0 1.5 1.0 0.5 0.0
f1 (ppm)

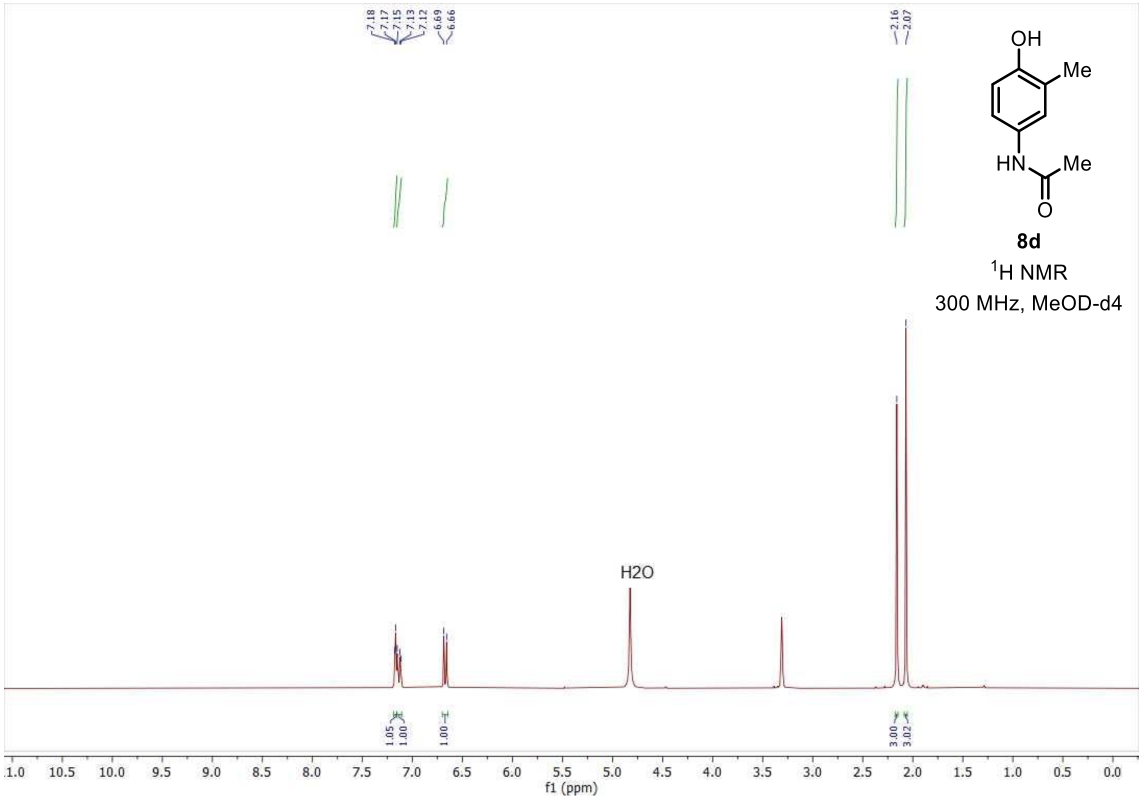
7.18
7.17
7.15
7.13
7.12
6.69
6.66
2.16
2.07
OH
Me
HN
Me
O
8d
1H NMR
300 MHz, MeOD-d4
H2O
1.05
1.00
1.00
3.00
3.02
f1 (ppm)
